# Plant-derived extracts or compounds for Helicobacter-associated gastritis: a systematic review of their anti-Helicobacter activity and anti-inflammatory effect in animal experiments

**DOI:** 10.1186/s13020-025-01093-2

**Published:** 2025-04-22

**Authors:** Danni Chen, Wenlai Wang, Xiangyun Chen, Ning Liang, Jiawang Li, Wei Ding, Hongrui Zhang, Zhen Yang, Hongxia Zhao, Zhenhong Liu

**Affiliations:** 1 Dongzhimen Hospital, Beijing University of Chinese Medicine, No. 5 Haiyuncang, Dongcheng District, Beijing, 100700 China; 2 College of Traditional Chinese Medicine, Beijing University of Chinese Medicine, No. 11 Bei San Huan Dong Lu, Chaoyang District, Beijing, 100029 China; 3 Institute for Brain Disorders, Beijing University of Chinese Medicine, Beijing, 100700 China; 4 Institute of Basic Theory for Chinese Medicine, China Academy of Chinese Medical Sciences, No. 16 Nanxiaojie, Dongzhimen Nei, Dongcheng District, Beijing, 100700 China; 5 Institute of Basic Research in Clinical Medicine, China Academy of Chinese Medical Sciences, Beijing, 100700 China

**Keywords:** Helicobacter-associated gastritis, Phytotherapy, Compound, Plant extract, Inflammation, Helicobacter

## Abstract

**Background:**

Helicobacter infection, which is the leading cause of gastritis and stomach cancer, has become common worldwide. Almost all Helicobacter-infected patients have chronic active gastritis, also known as Helicobacter-associated gastritis (HAG). However, the eradication rate of Helicobacter is decreasing due to the poor efficacy of current medications, which causes infection to recur, inflammation to persist, and stomach cancer to develop. Natural components have robust antibacterial activity and anti-inflammatory capacity, as confirmed by many studies of alternative natural medicines.

**Purpose:**

This article aimed to conduct a comprehensive search and meta-analysis to evaluate the efficacy of anti-Helicobacter and anti-inflammatory activities of plant-derived extracts or compounds that can treat HAG in animal experiments. We intended to provide detailed preclinical-research foundation including plant and compound information, as well as the mechanisms by which these plant-derived substances inhibit the progression of Helicobacter infection, gastritis and neoplasms for future study.

**Methods:**

The systematic review is aligned with the guidelines outlined in the Preferred Reporting Items for Systematic Reviews and Meta-Analyses (PRISMA) statement, and the protocol was registered in PROSPERO (CRD42024527889). An extensive search was performed across multiple databases, including PubMed, Scopus, Web of Science, Embase, China National Knowledge Infrastructure (CNKI), the Chinese Scientific Journal database (VIP), the Wanfang database, and the China biomedical literature service system (SinoMed), up until November 2023. Meta-analysis on Review Manager software (RevMan 5.4) estimating anti-Helicobacter and anti-inflammatory activity was performed. We used the Systematic Review Center for Laboratory Animal Experimentation (SYRCLE) risk of bias tool to evaluate the risk of bias of each study included.

**Results:**

Our study encompassed 61 researches, comprised 36 extracts and 37 compounds improving HAG by inhibiting Helicobacter infection, the inflammatory response, oxidative stress, and regulating apoptosis and proliferation. Sixteen families especially Asteraceae, Fabaceae and Rosaceae and nine classes including Terpenoids, Alkaloids, Phenols, and Flavonoids may be promising directions for valuable new drugs. The Meta-analyse demonstrated the plant-base substance treatments possess significant anti-Helicobacter and anti-inflammation activity comparing to control groups. The included plants and compounds confirmed that signaling pathways NF-κB, JAK2/STAT3, MAPK, TLR4/MyD88, PI3K/AKT, NLRP3/Caspase-1 and NRF2/HO-1 play a key role in the progression of HAG.

**Conclusion:**

Plant-derived extracts or compounds actively improve HAG by modulating relevant mechanisms and signaling pathways, particularly through the anti-Helicobacter and inflammatory regulation ways. Further researches to apply these treatments in humans are needed, which will provide direction for the future development of therapeutic drugs to increase eradication rate and alleviate gastritis.

**Supplementary Information:**

The online version contains supplementary material available at 10.1186/s13020-025-01093-2.

## Introduction

Helicobacter infection causes progressive damage to the gastric mucosa, which can cause numerous diseases, including Helicobacter-associated gastritis (HAG), gastric or duodenal peptic ulcer disease (PUD), gastric cancer, and gastric mucosa-associated lymphoid tissue (MALT) lymphoma [1]. Helicobacter infection is the most prevalent cause of chronic gastritis (so-called HAG). Helicobacter initiates gastric endothelial and myelocyte cell responses, resulting in oxidative stress, an inflammatory response, and abnormal cell apoptosis, cell growth, and differentiation. In addition, Helicobacter-induced gastritis and subsequent disease progression, such as precancerous lesions and neoplasms due to failed bacterial eradication and poor inflammatory control, are important issues. The International Agency for Research on Cancer (IARC) has defined Helicobacter as a class I carcinogen, and either eradication of Helicobacter or attenuation of mutagenic inflammation can prevent gastritis from developing into gastric cancer [2]. HAG is a representative type of “inflammation-carcinogenesis," which means that chronic gastritis will develop into gastric cancer through different regulatory mechanisms if efficacy measures are absent. As described by the Correa cascade, the human model of gastric carcinogenesis slowly progresses from the following series of pathologic changes: superficial gastritis, chronic gastritis, atrophy, intestinal metaplasia (IM), dysplasia, and cancer [3–5]. Helicobacter is a risk factor for gastric tumor development, and anti-Helicobacter therapy is compulsory for preventing or treating malignant and precancerous lesions in the stomach.

PPI-based triple therapies (PPI-TTs) are predominantly therapeutic approaches for Helicobacter infection. The PPI-TTs comprise a proton pump inhibitor (such as omeprazole or pantoprazole) and two antibiotics. With the wide application of PPI-TTs, antibiotic resistance problems appear, which lead to eradication failure. The resistance of the first-line antibiotics clarithromycin and metronidazole is the greatest challenge. Clarithromycin resistance has increased to 15–30% [6] worldwide, and clarithromycin, metronidazole, and levofloxacin have resistance rates of 25%, 30%, and 20%, respectively, in 2,852 Helicobacter-treatment-initial patients in Europe [7]. The recommended empirical first-line therapy, bismuth-based quadruple therapy (BiQT), combines antibiotics with bismuth, which diminishes resistance to clarithromycin and metronidazole.

Owing to the decreased effectiveness of antibiotics against Helicobacter, many researchers are focusing on discovering and developing potent plant-derived drugs that favor complete eradication or are advantageous for eradication. Many medicinal plants and isolated bioactive compounds have been validated for their antibacterial, anti-inflammatory activity, and preventive effect on abnormal cell growth and development both in vitro and in vivo. To date, no systematic review has been conducted on the utilization of phytopharmaceuticals in HAG treatment. Our objective is to perform a comprehensive analysis that highlights the in vivo efficacy of phytopharmaceuticals on HAG and elucidates the mechanisms by which these plant-derived substances inhibit the progression of infection, gastritis, and neoplasms. This systematic review is distinct from traditional reviews in that we adhere to a controlled research strategy, which minimizes potential sources of bias throughout the entire review process. This includes the objective, literature search, discovery of relevant literature, evaluation of the quality of relevant research, data summaries or analyses, and conclusions [8]. The dependability and correctness of conclusions are enhanced by the clear methodologies employed in systematic reviews, which serve to reduce bias [9]. These will provide a better understanding of HAG and serve as a reference for further drug research on this field.

The Correa cascade describes the disease process, which begins with Helicobacter infection and ends with gastric carcinogenesis. Atrophic gastritis, intestinal metaplasia, and dysplasia are three precancerous lesions that indicate disease severity and an increased risk of carcinogenicity [8]. Plant-derived extracts and compounds from different species fulfil a role at different stages of this process. We included 61 studies treating HAG and assigned a number from 001 to 061 for them. (Table 1, 2).

**Table 1:** Plant extract intervention for HAG

Refs.	Extraction solvent (Part)	Plant species	Family	Male/Female Animal; Model	Dose; Duration		Anti-Hecilobater activity
002 Lee JY et al. 2023 [10]	Ethanol (Leaf)	*Maclura tricuspidata*	Moraceae	M C57BL/6 mice; Hp (4 × 10^9^ CFU/mL), 100 μL orally, 3 times/w, for 2w	10, 20 mg/kg; 6w		1. Gastric CLO↓ 2. Stool Hp antigen△ 3. Serum anti-Hp IgG↓
003 Wu H et al. 2023 [11]	Chloroform (Dried tuber)	*Corydalis yanhusuo* (Y. H. Chou & Chun C. Hsu)	Papaveraceae	M mice; Hp (10^8^ CFU/mL), 0.2 mL orally, tri-weekly, for 4w	100, 200, 400 mg/kg; 2w		1. Gastric RUT↓ 2. Serum anti-Hp IgG↓
004 Yuan Y et al. 2023 [12]	UN (UN)	*Persicaria capitata* (Buch. -Ham. ex D. Don) H. Gross	Polygonaceae	Sprague–Dawley rats; pre-treated with (2 g/L sodium bicarbonate), Hp (10^11^ CFU/L), 1.5 mL orally, 5 times at 1d-interval	1.58 g/kg/d; 2w		1. Gastric Hp density (CFU)↓
005 Jin Z et al. 2022 [13]	Water, Ethanol (UN)	*Parnassia palustris* L	Celastraceae	M Kunming mice; Hp (1.2 × 10^9^ CFU/mL), 200 μL orally, 6 times at 1d-interval	20 mg/d; 5d		1. Gastric RUT↓
006 Lee TH et al 2022 [14]	Salt baked in bamboo barrel	(Bamboo)	UN	M C57BL/6 mice; Hp (10^8^ CFU), 200 μL orally, daily, for 8w	83.3, 166.6, 333.2 mg/kg; 5d		1. Gastric mRNA of *CagA* gene: T, ST, BST score 0; BS↓
007 Song MY et al 2022 [15]	Ethanol (Korean Propolis)	(Korean Propolis)	UN	M C57BL/6 mice; pre-treated with 0.2 mL (5% sodium bicarbonate) 3d, Hp (5 × 10^9^ CFU/mL), orally, 4 times at 2d-interval, for 1w	0.2 mL/kg of 200 mg/kg, 3-times/w; 4w		1. Serum IgG↓ 2. Gastric CLO↓ 3. Gastric gene expression of Hp *16S rRNA*, *Ss1* strain, *SsA* strain, *UreA*, *NapA*↓ 4.Gastric CagA protein↓
008 Mayyas A et al. 2021 [16]	Ethanol (Peel)	*Punica granatum* L	Lythraceae	F Wistar rats; pre-treated with streptomycin (5 mg/mL) 3d, Hp (2.7 × 10^9^ CFU/mL), orally, twice/d at 4 h-interval, for 8d	50 mg/kg, twice daily; 8d		1. Urease activity↓ 2. Hp positive rats (HE-stained sections)↓
009 Park JM et al. 2021 [17]	No extract solvent. Shelled kernels pellet (Shelled kernels)	*Juglans regia* L	Juglandaceae	M C57BL/6 mice; pre-treated with pantoprazole (20 mg/kg) 3 times/w, Hp (10^8^ CFU/mL), 0.1 mL orally, then [CAG and cancer model: AIN-46A containing 7.5% NaCl pellet diet for 24w, 36w respectively]	100, 200 mg/kg; 24w or 36w		UN
011 Tripathi A et al. 2021 [18]	Ethanol (Leaf)	*Capparis zeylanica* L	Capparaceae	M Wistar rats; pre-treated with naproxen (30 mg/kg), Hp (10^8^ CFU/mL), orally 1 mL/d, for 7d	120, 240, 360, 480 O.D. mg/kg; 8w	1. Gastric RUT: 360, 480 mg/kg eradicated Hp 2. Gastric Hp *HrgA*, *16S rRNA* gene: 360, 480 mg/kg eradicated	
014 Park JU et al. 2020 [19]	Ethanol (Unripe fruit)	*Rubus crataegifolius* Bunge	Rosaceae	M Balb/c mice; Hp (2 × 10^8^ CFU), 0.5 mL orally	100 mg/kg BW/d; 8w	1. Average number of the viable bacteria in gastric (CFU)↓	
014 Park JU et al. 2020 [19]	Ethanol (Stem bark)	*Ulmus macrocarpa* Hance	Ulmaceae	M Balb/c mice; Hp (2 × 10^8^ CFU), 0.5 mL orally	100 mg/kg BW/d; 8w	1. Average number of the viable bacteria in gastric (CFU)↓	
016 Lee HA et al. 2018 [20]	Ethanol (Root)	*Allium hookeri*	Amaryllidaceae	M C57BL/6 mice; Hp (10^9^ CFU), orally, 3 times at 3d-interval	25, 50, 100 mg/kg/d; 4w	1. CLO score, CLO positive rate dose dependently↓	
017 Sandhya S et al. 2018 [21]	Methanol, Chloroform (Root)	*Tephrosia maxima.* L	Fabaceae	Albino Swiss mice; Pre-treated with Naproxen (30 mg/kg) 3d, Hp (10^8^ CFU/mL), 1 mL orally, for 7d	120 mg/kg/d; 4, 7, 10w	1. Gastric CFU↓, RUT↓, *16S rRNA* of Hp△	
020 Ik LY et al. 2019 [22]	Ethanol/Hot water (Unripe ruit)	*Rubus crataegifolius* Bunge	Rosaceae	M C57BL/6 mice; Hp (2 × 10^8^ CFU), orally, 3 times	150 mg/kg; 4w	1. Log_10_CFU/stomach↓, no significance	
020 Ik LY et al. 2019 [22]	Ethanol/Hot water (Stem bark)	*Ulmus macrocarpa* Hance	Ulmaceae	M C57BL/6 mice; Hp (2 × 10^8^ CFU), orally, 3 times	150 mg/kg; 4w	1. Log_10_CFU/stomach↓, no significance	
020 Ik LY et al. 2019 [22]	Ethanol/Hot water (Ripe ruit)	*Gardenia jasminoides* J. Ellis	Rubiaceae	M C57BL/6 mice; Hp (2 × 10^8^ CFU), orally, 3 times	150 mg/kg; 4w	1. Log_10_CFU/stomach↓, no significance	
024 Kim A et al. 2016 [23]	Dried powder (UN)	*Angelica keiskei*	Apiaceae	C57BL/6 mice; Hp (10^9^ /mL) orally, thrice for a 3-day period, for 7w, AIN-76A diet for 7w	3% NAC, 8% AK; 7w	1. Gastric Hp *16S rRNA*↓	
025 Kim AY et al. 2016 [24]	A. Freeze-dried powder (Root); B. L-ascorbic acid catalyze a myrosinase reaction to get a new powder (Root)	*Brassica rapa* L	Brassicaceae	F C57BL/6 mice; Hp (1109 CFU), orally, 3 times at 2d-interval, for 1w	A: 200 mg/kg/d, B: 100, 200 mg/kg/d; 4w	1. CFU/g stomach↓ 2. Gastric mucosa IHC for Hp colonization↓ 3. Gastric urease activity↓ 4. Serum anti-Hp IgG: only high dose of HY3↑	
026 Ye H et al. 2015 [25]	Volatile oil of plant (UN)	*Chenopodium ambrosioides* L. */Dysphania ambrosioides* (L.)	Amaranthaceae	M Kunming mice; Hp (1.2 × 10^9^ CFU/mL), 0.4 mL orally, 5 times at 1d-interval	49.32 mg/kg; 4w	1. Gastric RUT↓	
027 Zhang S et al. 2015 [26]	Ethanol (Whole plant)	*Polygonum capitatum*	Polygonaceae	M/F (1:1) C57BL/6 mice; Hp (10^8^ CFU/mL), 0.1 mL orally, 3 alternate days, for 9d	32, 64, 128 μg; 2w	1. Eradication rate of gastric Hp (CFU): (CLA + AMX + OMZ)- 100%; PCE- 89%; PCE + AMX- 93% 2. Gastric mRNA of *CagA*△	
029 Park JM et al. 2014 [27]	Ethanol (UN)	(Licorice)	Fabaceae	M B6.129P2-IL10^tm1Cgn/J^; Hp (10^9^ CFU/mL), 0.1 mL orally, 4 times, for 1w then [cancer model: pellet diet AIN76 (7.5% NaCl) for 28w]	25, 50, 100 mg/kg; 24w	UN	
030 Yamada T et al. 2014 [28]	Freeze-dried products A. RB1 (glucoraphanin + glucoraphenin) B. RB2 (glucoraphanin)	× *Raphanobrassica karpechenkoi*	UN	M Mongolian gerbils; Hp (10^8^ CFU), 1 mL orally	2%; 11w (week2—12)	UN	
031 Brown JC et al. 2011 [29]	Powder (Skin)	*Vitis rotundifolia* Michx	Vitaceae	F C57BL/6 mice; Pre-treated with MGS powder, Hp (10^7^–10^8^ CFU/mouse), 0.25 mL orally, 3 times at 2-d-interval, for 5d	5%, 10%, 0.5 mg/mouse, daily; 1w	1. Gastric log CFU /g↓ but no significant difference with infected untreated mice	
033 Pastene E et al. 2010 [30]	Absorber resin Sepabeads SP-850 (Ripe fruits Peel)	*Malus domestica* cv Granny Smith */Malus domestica* (Suckow) Borkh	Rosaceae	C57BL6/J mice; Hp (10^9^ CFU/mL), 0.3 mL orally, 4 times at 2-d-interval	200 μL of 150, 300 mg/kg/d; 20d	1. Gastric Hp *16S rRNA* (lg Hp g/stomach): 0	
034 Gu L et al. 2007 [31]	Freeze-dried powder (Garlic bulb)	(Garlic)	UN	M Gerbillinae; Hp (10^9^ CFU/mL), 0.4 mL/4d orally, 5 times	1 mL/100 g/d; 4w	UN	
035 Paraschos S et al. 2007 [32]	Ethylacetate + Methanol (Resin/gum)	*Pistacia lentiscus* var. chia Poir */Pistacia lentiscus* L	Anacardiaceae	F C57BL/6 mice; Hp (10^8^ CFU), 100 μL orally, 3 times within a week	0.75 mg/d; 3 m, [an extra group pre-treated]	1. Serum anti-Hp IgG: - 2. Gastric tissue Hp (log CFU/g)↓ 3. Gastric tissue Hp colonization grades (Histopathology)↓	
036 Murakami M et al. 2005 [33]	Special process (Rice)	(Aqueous rice)	UN	M Mongolian gerbils; Hp (10^9^ CFU/mL), 0.8 mL orally	UN; 10w	1. Gastric CFU of Hp↓ no significant difference with infected untreated mice 2. Serum anti-Hp IgG↓	
037 Otsuka T et al. 2005[34]	Water (Fruit)	*Prunus mume* Sieb. et Zucc	Rosaceae	M Mongolian gerbils; Hp (10^8^ CFU/mL), orally	1%, 3%; 9w	1. Stomach Hp *ureaseA* gene↓ 2. Serum anti-Hp IgG△	
038 Souza Mdo et al. 2009 [35]	Hydro-Ethanolic (Stem bark), Dichloromethanic fraction (Stem bark)	*Calophyllum brasiliense* Cambess	Calophyllaceae	M Wistar albino rats/ Swiss-Webster Mice; Pre-treated with acetic acid (0.03 mL, 20%), Hp (6 × 10^8^ CFU/mL), 1 mL orally	50, 100, 200 mg/kg HEECb, 100, 200 mg/kg DCMF; UN	1. Negative rate of RUT↑	
039 Ruggiero P et al. 2009 [36]	Fermentation process (UN)	(Grape)	UN	BALB/c mice; Hp 10, 50 μg, two oral each other day	5 mL/d; UN	1. CFU/stomach↓ no significant difference with untreated infected mice 2. Gastric Hp or VacA localization (IHC): (Red Wine + Green Tea)↓	
039 Ruggiero P et al. 2009 [36]	Distilled Water (Leaf)	(Green tea)	UN	BALB/c mice; Hp 10, 50 μg, two oral each other day	5 mL/d; UN	1. CFU/stomach↓ no significant difference with untreated infected mice 2. Gastric Hp or VacA localization (IHC): (Red Wine + Green Tea)↓	
041 Jeong M et al. 2015 [37]	Water, Ethanol (UN)	*Artemisia capillaris* Thunb	Asteraceae	F C57BL/6 mice; Pre-treated with Pantoprazole (20 mg/kg), 3 times, Hp (10^9^ CFU/mL), 0.1 mL orally, 4 times, for 1w, then [cancer model: pellet diet AIN76 (7.5% NaCl) for 24, 36w]	75 mg/kg, UN	UN	
041 Jeong M et al. 2015 [37]	Water, Ethanol (Leaf)	*Camellia sinensis* L */Inversodicraea tanzaniensis* Cheek	Podostemaceae	Same as the previous line	75 mg/kg, UN	UN	
042 Stoicov C et al. 2009 [38]	Ethanol (Leaf)	(Green tea)	UN	M C57BL/6 J mice; Hp (5 × 10^7^ CFU), 500μL orally, 3 times at 2d-interval	①pre-treated GT/infection /GT ②infection/GT.1%; 8w	1. Stomach H.felis *flaB* gene: A, B score 0, C↓	
043 Limuro M et al. 2002 [39]	Water- ethanol mixture (Clove)	*Allium sativum* L	Amaryllidaceae	M Mongolian gerbils; Hp (2:0 £ 10^8^ CFU), 0.5 mL orally	1%, 2%, 4%; 6w Or 4%; 4w	1. Stomach colonies of Hp (CFU): -	
054 Ishizone S et al. 2007 [40]	Distilled water, enzyme-catalyzed reaction (Rice)	*Oryza sativa* L	Poaceae	M Mongolian gerbils; Hp (10 CFU), 0.8 mL orally	12w	1. Gastric Hp ( +) incidence (IHC)↓; CFU△ 2. Serum anti-Hp IgG↓ 3. Gastric immunostainging slice of Hp↓	
058 Ma X et al. 2020 [41]	Ethylacetate (Rhizome)	*Alpinia officinarum* Hance	Zingiberaceae	M BALB/c mice; Pretreated with mixed antibiotic solution (0.3 mL/d, for 3d), Hp (10^9^ CFU/mL), orally 0.3 mL/2d, for 14d	0.09, 0.18, 0.36 g/kg/d; 3w	1. RUT↓ 2. Gastric juice PH↓	
059 Zhao X 2006 [42]	Ethanol (Resin /Gum)	(Mastic)	UN	Gerbillinae; Hp (10^9^ CFU), 0.5 mL orally, 2 times at 6 h-interval	3.75, 7.5, 15 mg/200μL; 0.2 mL/d, 2w	1. Hp clearance rate (Hp *16S rDNA*)↑

**Table 2:** Plant-derived compound intervention for HAG

**Refs**	**Compound**	**Origin species**	**Alternative name**	**Family**	**Male/Female Animal; Model**	**Dose; Duration**	**Anti-Hecilobater** **activity**
001 [43] Gobert AP et al. 2023	2-hydroxybenzylamine	(Buckwheat)	Buckwheat	Polygonaceae	FVB/N INS-GAS mice; Hp (10^9^) , 0.2 mL orally, 2 times/2d	1, 3 mg/mL; 7w	1. Stomach CFU -
010 [44] Shi M et al. 2021	*Chaenomeles* *speciosa *total triterpenoids	*Chaenomeles speciosa *(Sweet) Nakai	Mugua, Quince	Rosaceae	M C57BL/6J mice; Pretreated with mixed antibiotic solution (0.3 mL/d, for 7d), Hp (10^9^CFU/mL), 0.30 mL orally, at 1d-interval, for 14d	20 mL/kg of 50, 100 mg/kg, 1 times/d; 4w	1. Gastric CLO↓
012 [45] Jung DH et al. 2020	H-002119-00-001/β-caryophyllene	*Syzygium aromaticum*	Cloves	Myrtaceae	M C57BL/6 mice; Hp (5x10^9^CFU/mL), 200 μL orally, 3 times at 2d-interval	5 mL/kg of 100, 200, 500 mg/kg; 2w or 4w	1. Gastric CLO ↓ 2. Gastric Hp *16S rRNA*↓
013 [46] Kim SE et al. 2020	Phytoncide	*Pinus koraiensis*	Pinecones	Pinaceae	M C57BL/6 mice; Pre-treated with 5% NaHCO_3_ (0.2 mL, for 3d), Hp (5x10^9^CFU/mL), 0.2 mL orally, 3 times at 12 h-interval, for 2d	100, 200, 400 mg/kg/d; 2w	1. Blood anti-Hp IgG↓ 2. CLO↓ 3. *CagA* gene expression↓
015 [47] Chen ME et al. 2018	①Baicalin, ②Baicalein	*Scutellaria baicalensis *Georgi	Huangqin	Lamiaceae	M C57BL/6 mice; Hp (10^9^CFU/mL), orally, totally 3 doses, on alternate days	0.2 mL of 80 μM [or+ 0.2 mL/d of LR-JB3 (2.5x10^7^ CFU/mL)]	1. Gastric Hp numbers↓ 2. Gastric *VacA* mRNA↓ 3. Serum IgA , IgM of Hp↓
018 [48] Yang JS et al. 2018	Eudesmin	*Fatsia polycarpa *Hayata		Araliaceae	M C57BL/6 mice; Hp (10^9^CFU), orally, totally 3 doses, on alternate days	5, 10, 20, 40 μM, 0.2 mL/d; 3d	1. Stomach Hp* 16S rRNA*↓
019 [49] Chang C et al. 2017	①Geniposide, ②Genipin	*Gardenia jasminoides *J. Ellis	Zhizi	Rubiaceae	M C57BL/6 mice; Hp (10^9^CFU), orally, totally 3 doses, on alternate days	①0.2 mL/d of 31, 62 mg/kg/d; 3d ②0.2 mL/d of 18, 36 mg/kg/d; 3d	1. Gastric Hp *VacA* mRNA↓, Hp *CagA *mRNA -
021 [50] Yanaka A et al. 2017	Sulforaphane	*Brassica oleracea *L.	Broccoli	Brassicaceae	*Nrf2(+/+)*, *Nrf2(-/-)* F C57BL/6 mice; Hp (5x10^7^CFU), high-salt diet (7.5% NaCl) for 2m	3 μmol/d of SGS [or+ homogenized Broccoli Sprouts]	1. Gastric Hp CFU ↓
022 [51] Zhang S et al. 2017	Quercetin	*Polygonum capitatum*		Polygonaceae	M Kunming mice; Hp (3x10^8^CFU/mL), diet mixed with Hp at 1mL/kg concentration fed for 1d	64 mg/kg; 3d	UN
023 [52] Cao D et al. 2016	18β-Glycyrrhetinic Acid (GRA)	*Glycyrrhiza glabra* L.	Liquorice, Gancao	Fabaceae	M Mongolian gerbils; Hp (10^8^CFU), 0.8 mL orally, 3 times at 2d-interval	0.1%; 10w	1. Gastric pH Value caused by Hp infection↓ 2. Gastric Hp colonization rate -
028 [53] Liu C et al. 2019	Epimedium polysaccharide (EPS), Trollius chinensispolysaccharide (TCPS), Siberian solomonseal rhizome polysaccharide (SSRPS), Astragalus polysaccharides (APS)	UN		Berberidaceae, Ranunculaceae, Liliaceae, Fabaceae	F BALB/c mice; Hp antigen (10 μg OVA or 5 μg rUreB), immunized intranasally, 3 times at 2w-interval	50 ug/mouse	Immunization with rUreB alone or (rUreB+PPSs) before Hp challenged orally: 1. Stomach *16S rDNA* of Hp: PPSs+ rUreB↓ 2. Serum IgG antibodies: PPSs+ rUreB no difference with rUreB alone 3. Stomach homogenate IgA: rUreB + SRPS/APS↑ 4. Intestinal lavage fluid IgA: rUreB+ TCPS/APS↑
031 [29] Brown JC et al. 2011	Quercetin /3, 3′, 4′, 5, 6- pentahydroxyflavone	*Vitis rotundifolia *Michx.	Muscadine grape	Vitaceae	F C57BL/6 mice; Pre-treated with NaHCO_3_(0.1 mL of 0.5 mol), Hp (10^7-8^CFU), 0.25 mL orally, 3 times at 2d-interval, for 5d	5%, 10% MGS powder Or 0.5 mg of 25 mg/kg quercetin; 11w (all fed 1w before infection)	1. Gastric log CFU /g↓ no significant difference with infected untreated mice
032 [54] Suk KT et al. 2011	2-4 polymer urushiol	*Rhus verniciflua *Stokes*/* *Toxicodendron vernicifluum *(Stokes) F. A. Barkley	Lacquer Tree	Anacardiaceae	F C57BL/6 mice; Pre-treated with NaHCO_3_(200 μL of 0.2 N), Hp (10^9^CFU), 500 μL/d orally, a total of 19 inoculations (3 times a week), for 1w	0.128 mg/mL/d; 1w	1. Hp eradication rate: urushiol 33%, Triple therapy 75%, (Triple therapy + urushiol) 100%
040 [55] Toyoda T et al. 2007	Nordihydroguaiaretic acid (NDGA)	*Larrea tridentata *DC Coville	Creosote Bush	Zygophyllaceae	M Mongolian gerbils; Hp (10^8^CFU/mL), 0.8 mL, 1 mL orally, [chemical carcinogen, N-methyl-N-nitrosourea, 20w]	0.01%, 0.05%, 0.25%; + AIN93G diet; 44w	1. Stomach *Urease A* gene△ 2. Serum anti-Hp IgG-
040 [55] Toyoda T et al. 2007	Arctigenin	*Arctium * *lappa *L	Niubangzi	Asteraceae	M Mongolian gerbils; Hp (10^8^CFU/mL), 0.8 mL, 1mL orally, [chemical carcinogen, N-methyl-N-nitrosourea]	0.1%; + AIN93G diet; 44w	1. Stomach *Urease A* gene△ 2. Serum anti-Hp IgG-
044 [56] Wu HM et al. 2024	Epiberberine (EPI)	*Coptis chinensis *Franch.	Huanglian	Ranunculaceae	F C57BL/6J mice; Hp (10^10^CFU), 0.4 mL orally, totally 4 times every 2 days	50, 100, 200 mg/kg; 2w	1. Stool antigen test and RUT: Hp clearance rates: (OMZ+CLA+AMX) -100%; low, medium and high-dose of EPI 33.3%, 50%, 66.7%
045 [57] Li G et al. 2023	Neutral corn protein hydrolysate (CPN)	*Zea mays *L.	Corn gluten meal	Poaceae	M KunMing mice; Hp (10^9^CFU/mL), 0.4 mL orally, on alternate days, 7 times in total	200, 400, 600 mg/kg·bw; Pre-treated for 2w before infection	1. Gastric CFU/g ↓
046 [58] Tang Q et al. 2023	Coptisine	*Coptis chinensis *Franch.	Huanglian	Ranunculaceae	F C57BL/6J mice; Hp (10^9^CFU), 0.2 mL orally, 4 times at 2d-interval	50, 100, 150 mg/kg/d; 2w	Clearance rate: 1. Hp Stool Antigen: quadruple therapy (QT), Cop-M, Cop-H 83.33%, 83.33%, 100% 2. Stomach tissue CFU: QT, Cop-M, Cop-H all 100% 3. Stomach tissue staining: QT, Cop-M, Cop-H 100%, 83.33%, 100%
047 [59] Dai YY et al. 2022	Linolenic Acid- Metronidazole (Lla-MTZ)	UN	Flax, Soybeans, Rapeseed	UN	C57BL/6 mice; Hp (10^9^CFU), 0.5 mL orally, every other day on 5 times	24 mg/kg; 1 times/d for 3d	1. Gastric mucosa of Hp CFU: OMZ+ Lla-MTZ did best
048 [60] Su T et al. 2019	①Artemisinin (ART) ②Artesunate(ARTS) ③Dihydroartemisinin (DHA)	*Artemisia annua *L	Artemisiae Annuae, Qinghao	Asteraceae	C57BL/6 mice; Pre-treated with MNU (240 ppm), 3 times/w for 6 cycles, Hp (10^9^CFU), 0.2 mL orally, 3 times/w for 3w	60 mg/kg; 36w	UN
049 [61] Toyoda T et al. 2016	Curcumin	*Curcuma longa *L.	Turmeric	Zingiberaceae	M Mongolian gerbils; Hp (10^8^CFU/mL), 1mL orally	5000 ppm	1. *UreA *mRNA△
	Capsaicin	*Capsicum *spp.	Chili peppers	Solanaceae		100 ppm	
	Piperine	*Piper nigrum *L.	Black peppers	Piperaceae		100 ppm	
050 [62] Zhang X et al. 2015	Resveratrol	UN	Berries, Nuts, Peanuts, Grape skin	UN	M Kunming mice; Hp (10^8^CFU) orally, 3 times	100 mg/kg/d; 6w	1. Gastric Hp CFU -
051 [63] Kundu P et al. 2011	Curcumin /Diferuloylmethane	*Curcuma longa *L.	Turmeric, Jianghuang	Zingiberaceae	C57BL/6 mice; Hp (10^8^CFU) orally, twice during 3d	25, 50 mg/kg; 7d	1. Urease test: gastric Hp colonization all score 0 2. Gastric Hp gene *UreB*, *NapA*: all score 0
052 [64] De R et al. 2009	Curcumin /Diferuloylmethane	*Curcuma longa *L.	Turmeric, Jianghuang	Zingiberaceae	C57BL/6 mice; Hp (10^8^CFU) orally, twice during 3d	25 mg/kg; 7d	1. Gastric Hp *VacA*△
053 [65] Toyoda T et al. 2009	Caffeic Acid Phenethyl Ester (CAPE)	UN	Propolis	UN	M C57BL/6 mice; Hp (10^8^CFU), 1mL orally	0.01%, 0.03%, 0.1%; 10w	1. Serum Anti-Hp IgG△
055 [66] Takeda K et al. 2007	Auraptene (AUR)	*Citrus × aurantium *f.* aurantium*	Natsumikan /Citrus × natsudaidai Hayata	Rutaceae	M Mongolian gerbils; Hp (2x10^8^CFU), orally, twice during 2w	100, 500 ppm	1. Serum Anti-Hp IgG- 2. Gastric Hp colonization- 3. Stomach *urease A* gene: high dose group↓
056 [67] Liu B et al. 2003	Total secondary carotenoids	*Chlorococcum* Sp(microalgae)		Chlorococcaceae	F BALB/c mice; Hp (10^9^CFU), 0.15 mL orally, 3 times at 2d-interval	100 mg/kg/d; 2w	1. Gastric Hp CFU△
057 [68] Takagi A et al. 2000	Plaunotol	*Croton stellatopilosus *H. Ohba	Plaunoi	Euphorbiaceae	M BALB/c mice; Hp (10^9^CFU), orally, 3d	25-100 mg/kg/d; 4w	1. Serum Anti-Hp IgG↓
060 [69] Tian A et al. 2014	*Sophora alopecuroides *L. total alkaloids (TASA) [sophoridine (SR), matrine (MA), sophocarpine (SC), and lemannine (LMN)]	*Sophora alopecuroides *L.	555	Fabaceae	F BALB/c mice; Pre-treated with omeprazole (0.4 mg), Hp (2x10^8^CFU), 0.4 mL orally, twice in a day	2, 4, 5 mg/d	1. Gastric mucosa Hp *16S rDNA*↓
061 [70] Chen X et al. 2020	Palmatine	*Coptis chinensis *Franch.	Huanglian	Ranunculaceae	M Sprague-Dawley rats; Hp (1.5x10^8^CFU/mL), orally, at 1d-interval	10, 20, 40 mg/kg/ d	UN

*Tables 1 and 2 “↓ or ↑” indicates that the value is statistically significant compared to the control group; “△” indicates that there is an upward or downward change compared to the control group but is not statistically significant; “-” indicates that there is no change in the indicator or that the value presented is abnormal. “UN” means that the information is not described in the article

## Materials and methods

The present investigation was conducted in accordance with the guidelines outlined in the Preferred Reporting Items for Systematic Reviews and Meta-Analyses (PRISMA) statement. The study protocol has been registered on the PROSPERO website (CRD42024527889).

### Search strategy

To collect relevant data, an extensive search was performed across multiple databases, including PubMed, Scopus, Web of Science, Embase, China National Knowledge Infrastructure (CNKI), the Chinese Scientific Journal database (VIP), the Wanfang database, and the China biomedical literature service system (SinoMed), up until November 2023. Using the retrieval of the PubMed database as an example, Medical subject headings (MeSH) terms and Free-text phrases from the PubMed database were used. The text terms included: (“Helicobacter Infections” [Mesh] OR “Infections, Helicobacter” OR “Helicobacter Infection” OR “Infection, Helicobacter”) AND (“Plants” [Mesh] OR “Plants” OR “Plant” OR “Plants, Medicinal” [Mesh] OR “Medicinal Plant” OR “Plant, Medicinal” OR “Medicinal Plants” OR “Herbs, Medicinal” OR “Medicinal Herbs” OR “Herb, Medicinal” OR “Medicinal Herb” OR “Pharmaceutical Plants” OR “Pharmaceutical Plant” OR “Plant, Pharmaceutical” OR “Plants, Pharmaceutical” OR “Healing Plants” OR “Healing Plant” OR “Plant, Healing” OR “Plants, Healing”). Only publications in Chinese and English were included. The detailed search strategies of the above databases are attached in the Additional file 1.

### Eligibility criteria

The inclusion criteria for relevant articles are listed below: (1) Studies that meet the PICOS condition are included: P (Animals) refers to “Helicobacter-infected animals," and I (Interventions) refers to “Plant-derived compounds or plant extracts," C (Comparators) refers to “Comparative control group," O (Outcomes) refers to “Outcomes of anti-Helicobacter and anti-inflammatory activities," and S (Study designs) refers to “Controlled studies with separate treatment groups." (2) The primary outcomes of the study include anti-Helicobacter and anti-inflammatory effect simultaneously.

Studies that met the following criteria were excluded: (1) Editorials, reviews, clinical studies, theoretical researches, case reports, conferences, book chapters, and letters. (2) Articles that did not meet the PICOS criteria. (3) Treatment was not a single extract or a monomer compound. (4) Papers solely focused on in vitro or ex vivo studies.

### Study selection

Endnote X9 software was utilized to arrange the search results. Reviewers (including W. W. and X. C.) evaluated the literature separately after eliminating duplicates, taking into account the abstract and title. The whole texts of the studies would be retrieved, and their eligibility would be assessed using the established inclusion and exclusion criteria, if deemed pertinent. The study authors were contacted if more information was required. All disputes or disagreements among study selection were settled with the third (Z. L.).

### Data extraction

Two reviewers (N. L. and J. L.) independently extracted relevant data of the eligible studies using a standard Excel, respectively. Any controversy or disagreement among data extraction was reconciled with the third (Z. Y.). The relevant data was abstracted from eligible articles: the first author’s name, publication year, extraction solvent, part of the plant for extraction, plant species, family, compound name, anti-Helicobacter potency outcome, gastric histopathology, characteristic parameters of HAG, indicators of further exacerbations of HAG.

### Meta-analysis

The reviewer (D. C.) performed quantitative analysis by meta-analysis on Review Manager software (RevMan 5.4), considering anti-Helicobacter and anti-inflammatory activity, including CLO and RUT positive rates, IL-1β, and TNF-α protein levels. The WebPlotDigitizer software was used to extract data from images. Forest plots present data including events and total number of groups, mean, standard deviation, and effect size as study weight, risk ratio, or standardized mean difference with 95% confidence intervals (95% CI). Due to the heterogeneity across studies, we chose a random effect model for all the analysis. The funnel plots reflected the publication bias.

### Methodological quality assessment

Two reviewers (W. D. and H. Z.) independently assessed the methodological quality of the included studies using the Systematic Review Center for Laboratory Animal Experimentation (SYRCLE) risk of bias tool [9]. This tool includes ten items, each with a high, unclear, or low risk of bias. Any discrepancies were resolved by a senior member of the research (Z. L.).

## Results

### Study inclusion

**Fig. 1:**
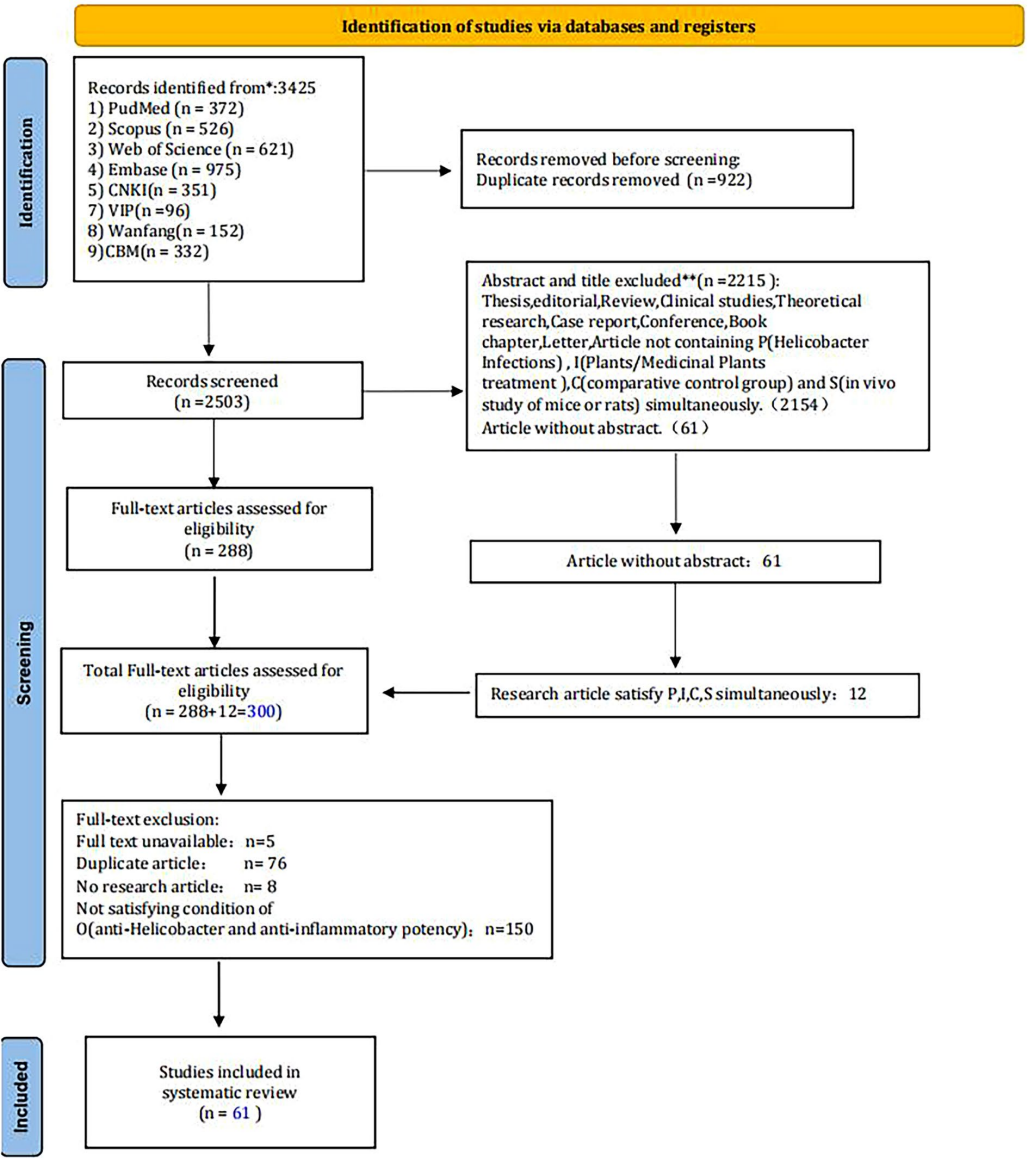
PRISMA flow diagram for the systematic review

**Fig. 2:**
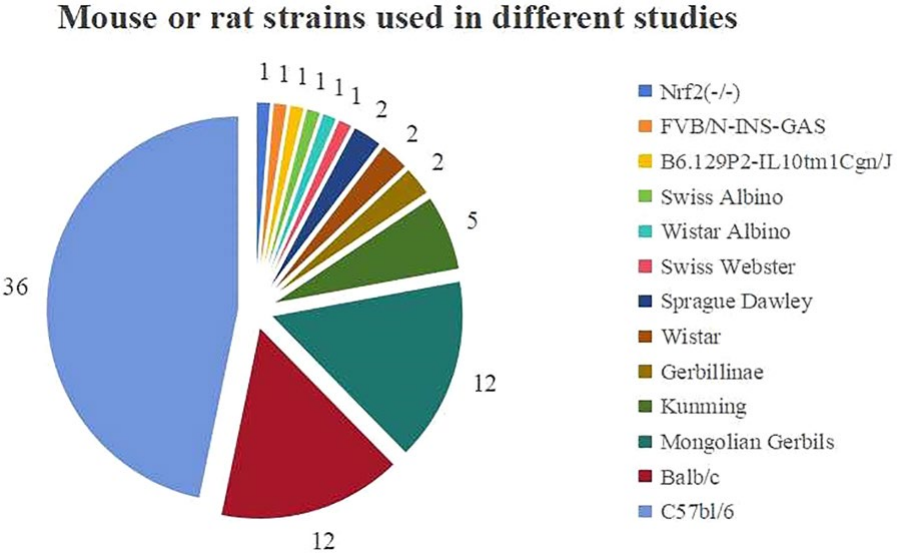
Mouse or rat strains used in different studies

After screening 2503 records, we identified 61 publications satisfying the inclusion criteria. Figure 1 presents the comprehensive and well-structured PRISMA flowchart. All 61 investigations that were implemented on mice or rats were conducted between 2003 and 2023. Thirteen animal strains were utilized to create the HAG models and treated with plant extracts or plant-derived compounds. The most frequently used strain was C57BL/6 mice (36 studies), and the top 2 and top 3 were BALB/c mice (12 studies) and Mongolian gerbils (12 studies), respectively (Figs. 1, 2).

### Assessment of risk of bias

**Fig. 3:**
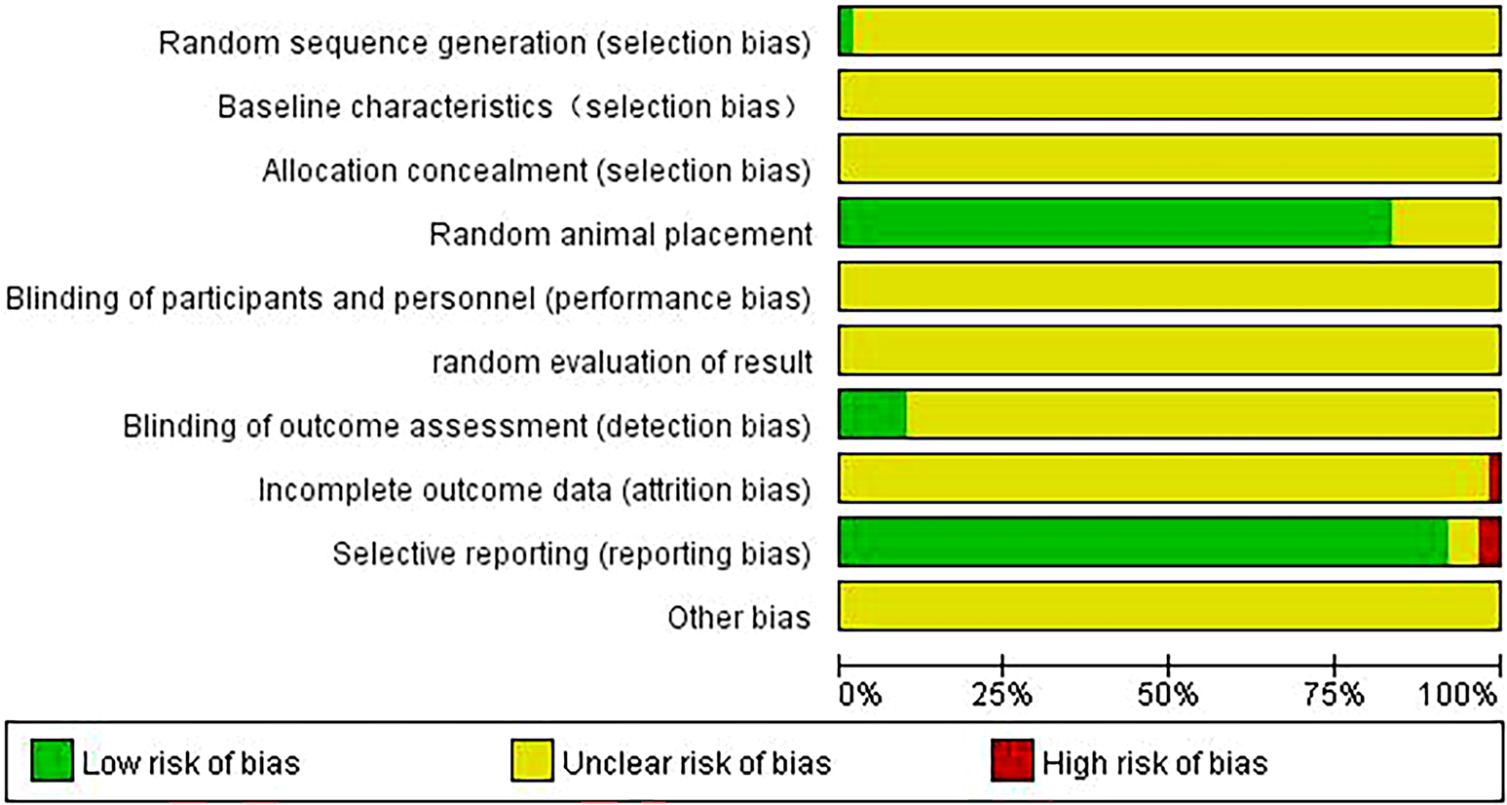
Risk of bias graph

**Fig. 4:**
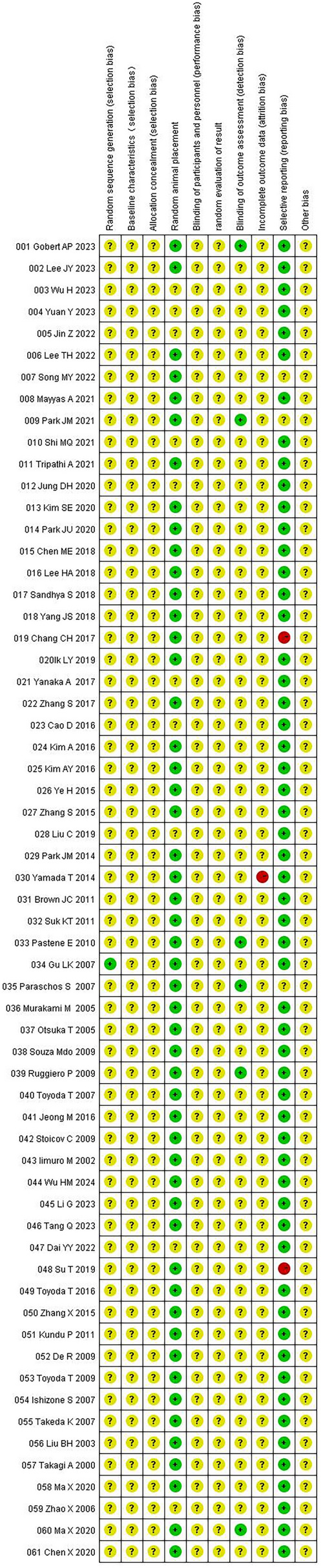
Risk of bias summary

One study used a random number table to randomize the animals into groups, and sixty studies did not report their randomization methods and were thus marked "unclear." Fifty-one included studies reported random animal placement, and the remaining studies did not mention such placement. Six studies provided elaborate “blinding of outcome assessment” information, whereas other studies were not known. One study incompletely reported outcome data, and the remaining studies did not have sufficient information to determine whether there was any loss of outcome data. Two studies did not fully report the expected results, three studies were uncertain, and the remaining studies fully reported the expected outcomes. In all included studies, information about “baseline characteristics," “allocation concealment," “blinding of participants and personnel," “random evaluation of result,” and “incomplete outcome data” was not available. Other causes, such as differences in modeling methodology, heterogeneity of the interventions, and variations in animal characteristics, may lead to evidence that is non-generalizable (Figs. 3, 4).

### Meta-analysis of anti-Helicobacter and anti-inflammatory effects

**Fig. 5:**
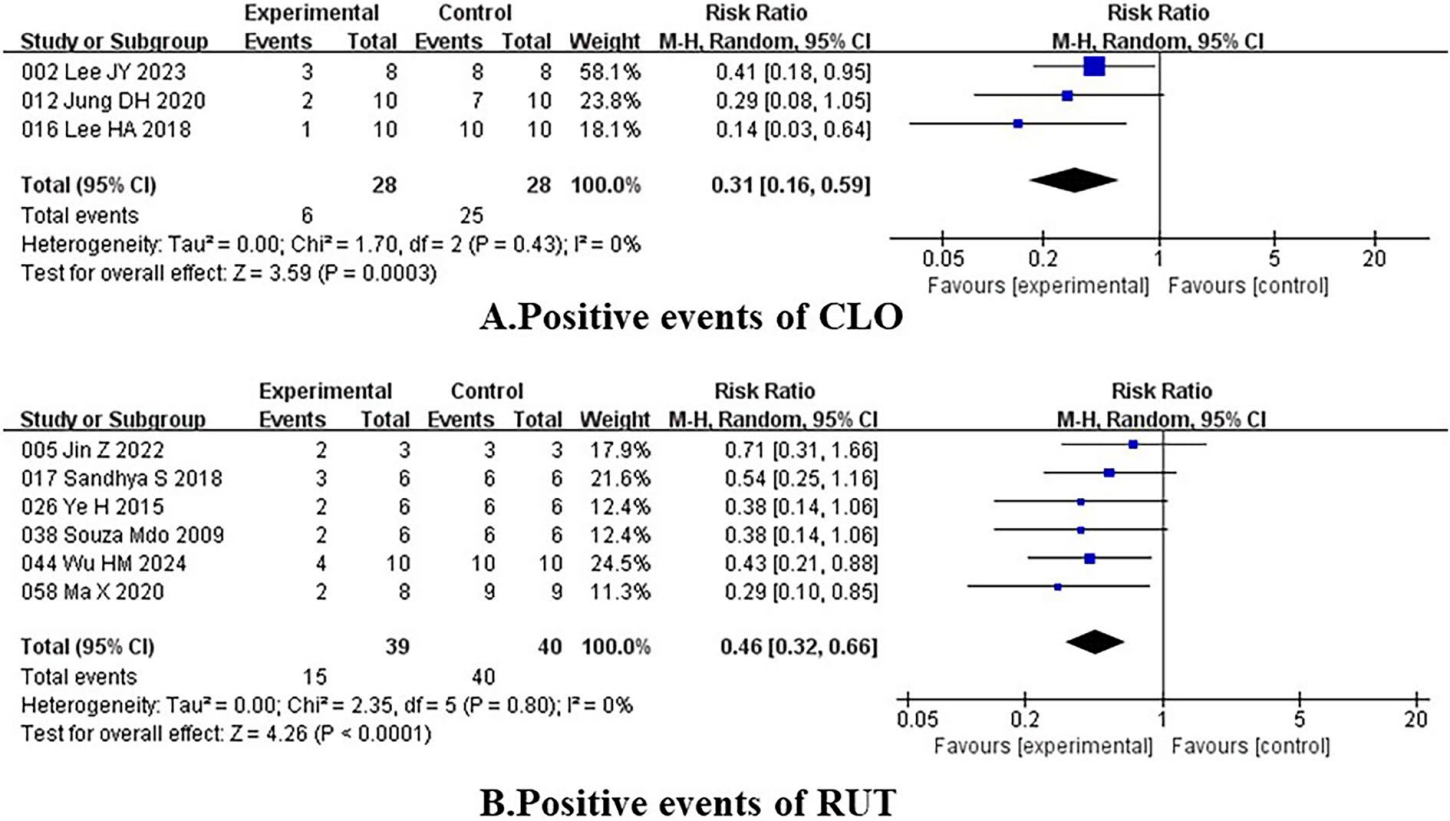
Forest plots of anti-Helicobacter activity. [**A** Positive events of CLO; **B** Positive events of RUT]

In order to examine phytomedicines’ curative effects of anti-Helicobacter and anti-inflammatory, meta-analysis were conducted, and the results supported our perspective. Among the 61 studies, three studies gave the Helicobacter positive events data after plant extracts or compounds treatment by the Campylobacter-Like Organism (CLO) test, and six studies gave the relevant data by the Rapid Urease Test (RUT). The study results presented in Fig. 5 demonstrate experiment groups in which gavaged animals plant extracts or compounds significantly reduced the Helicobacter positive rate of CLO (RR = 0.31, 95% CI 0.16 to 0.59, I^2^ = 0%, p = 0.0003) and RUT (RR = 0.46, 95% CI 0.32 to 0.66, I^2^ = 0%, p < 0.0001) (Fig. 5).

**Fig. 6:**
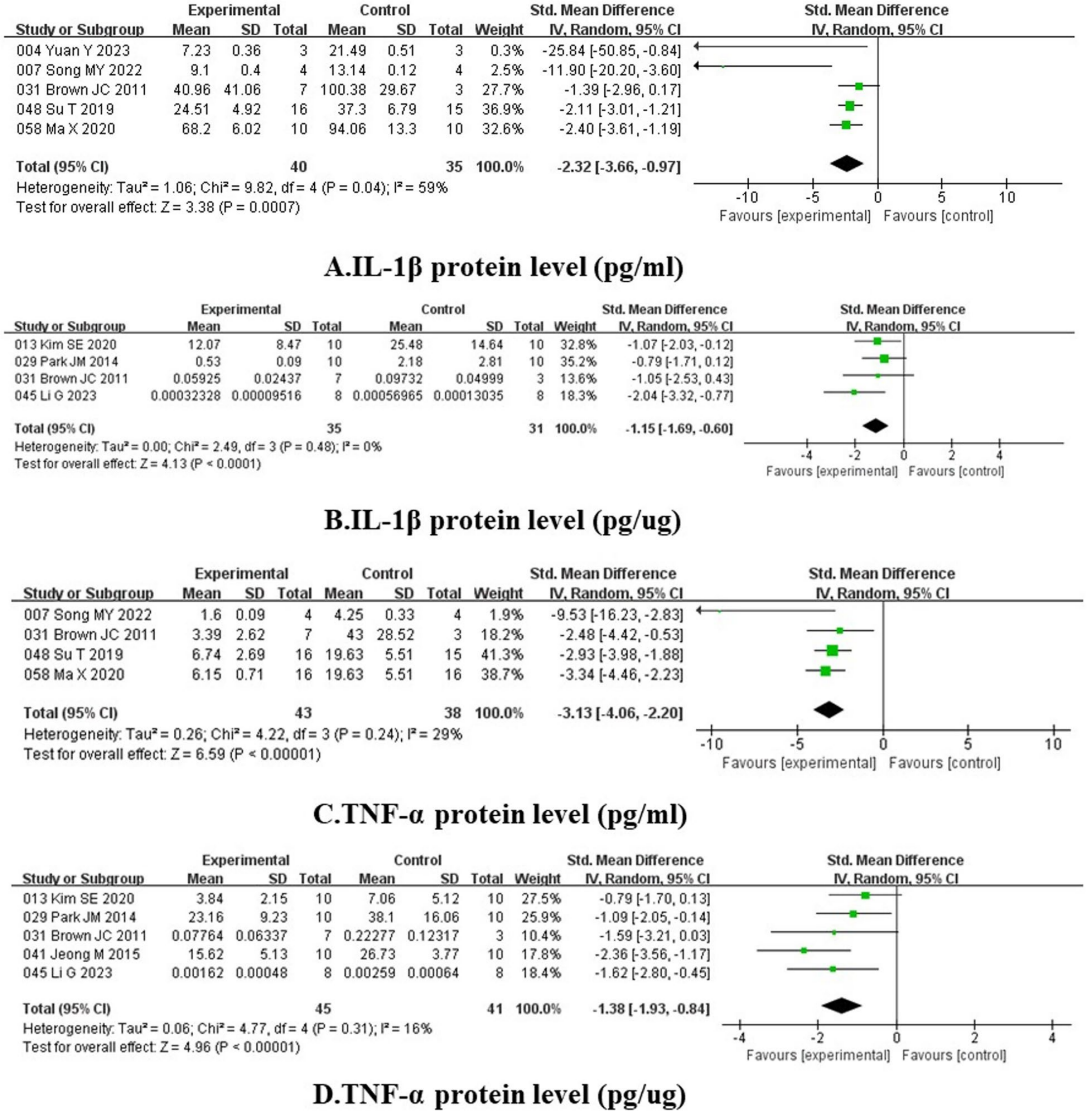
Forest plots of anti-inflammatory activity. [**A** IL-1β protein level (pg/mL), **B** IL-1β protein level (pg/ug), **C** TNF-α protein level (pg/mL), **D** TNF-α protein level (pg/ug)]

Interleukin-1β (IL-1β) and tumor necrosis factor-α (TNF-α) are major inflammatory mediators on HAG. Among the analyzed studies in Fig. 6, five substances significantly decreased IL-1β protein level (pg/mL) (SMD = − 2.32, 95% CI − 3.66 to − 0.97, I^2^ = 59%, p = 0.0007), four elements also declined IL-1β production (pg/ug) (SMD = − 1.15, 95% CI − 1.69 to − 0.60, I^2^ = 0%, p < 0.0001). The forest plots also reveal that plant origin substances groups have good potency on refraining TNF-α, and the data manifest TNF-α (pg/mL) (SMD = − 3.13, 95% CI − 4.06 to − 2.20, I^2^ = 29%, p < 0.00001), TNF-α (pg/ug) (SMD = − 1.38, 95% CI −1.93 to −0.84, I^2^ = 16%, p < 0.00001) (Fig. 6).

**Fig. 7:**
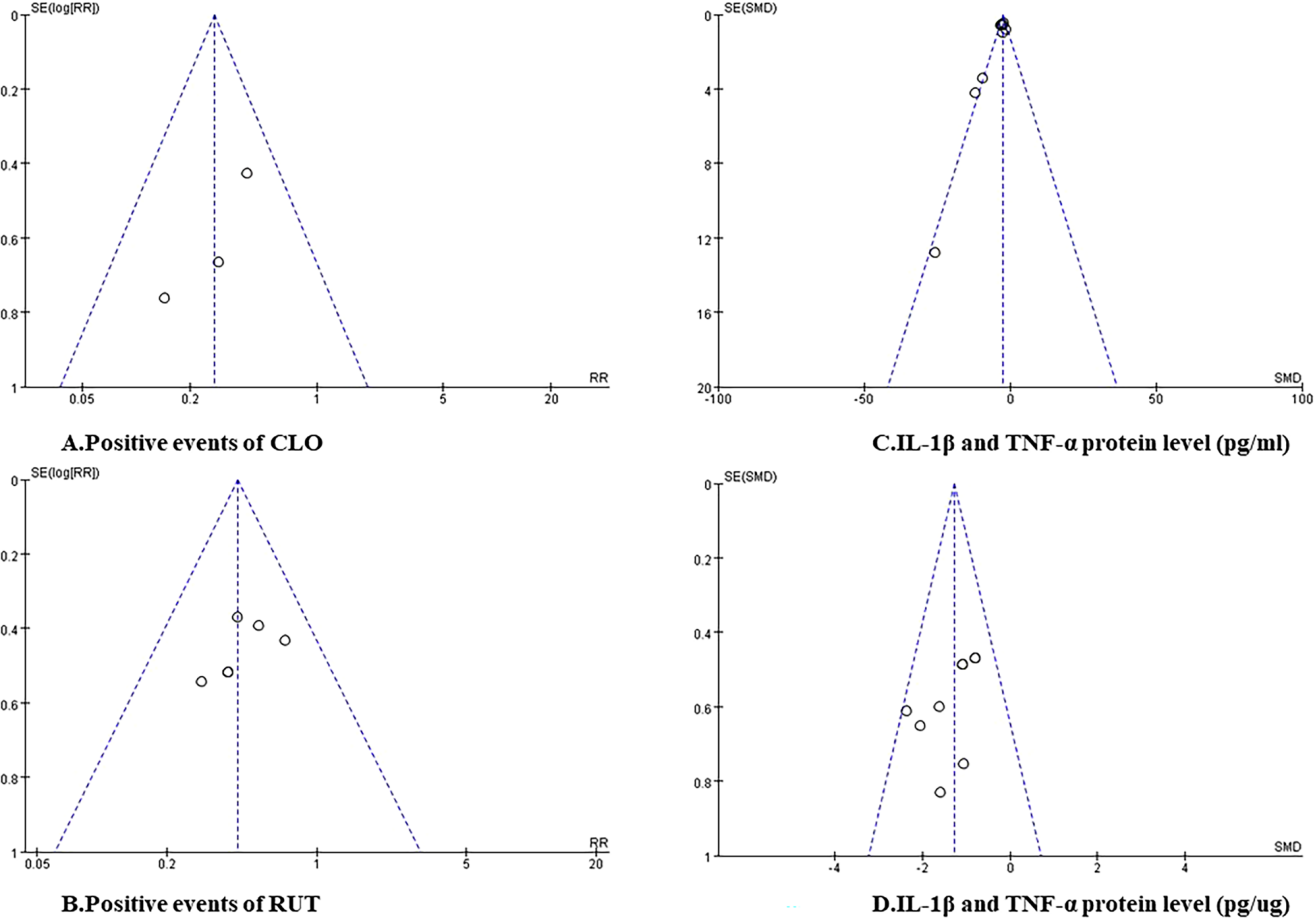
Funnel plots of different studies. [(**A** Positive events of CLO; **B** Positive events of RUT; **C** IL-1β and TNF-α protein levels (pg/mL); **D** IL-1β and TNF-α protein levels (pg/ug)]

We obtained funnel plots by including studies that employed parameters using the same unity. Outcomes reveal a mild asymmetrical distribution, which suggests there may exist publication bias (Fig. 7).

### Anti-Helicobacter activity of plant extracts and compounds

In this systematic review, plant-derived extracts or compounds that have good efficiency on HAG are summarized by tabling. Tables 1, 2, 3, 4 and 5 present fundamental knowledge and pharmacological facts on the anti-Helicobacter and anti-inflammatory activity of plant extracts and compounds in the treatment of HAG.

**Table 3:** Method of Helicobacter detection inanimals

Name	Abbreviation	Method
Campylobacter-Like Organism test OR Rapid Urease Test	CLO OR RUT	1. Test stomach tissue by CLO/RUT kit 2. Rationale: Helicobacter urease 3. Score by color change
Helicobacter urease activity	/	1. Mixes stomach tissue with urease activity test solution and read the absorbance 2. Rationale: Helicobacter urease
Helicobacter Stool Antigen	HPSA	1. Test animals’ stool antigen of Helicobacter
Helicobacter Colony-Forming-Unit	CFU	1. Homogenize stomach tissue and plate onto Medium after dilution, incubate at 37℃ for 48 h under microaerophilic conditions, finally count the colonies
Anti-Helicobacter IgG/A/M (Helicobacter IgG/A/M antibodies)	IgG, IgA, IgM	1. Test serum, blood samples from eye, tail vein by ELISA assay kit
Helicobacter gene detection	*CagA*, *VacA*, *Ss1*, *UreA*, *SsA*, *NapA*, *16S DNA* and so on	1. Collect gastric tissue and test by qRT-PCR 2. Rationale: Extract Helicobacter gene for PCR test
Histopathological analysis	/	1. Giemsa stain of stomach paraffin slices, and confirm Helicobacter density or count bacteria under microscope

**Table 4:** HAG-related regulatory mechanism of plant extract

Refs.	Plant species	Gastric Histopathology	Infammatory Biomarkers	Oxidative Stress Biomarkers	Apoptosis OR Proliferation Biomarkers	Pathway Signature Molecule
[10]	*Maclura tricuspidata*	ulcers, erosion, inflammation↓	IL-8↓			
[11]	*Corydalis yanhusuo* (Y.H.Chou & Chun C.Hsu)	hyperemia, epithelial cell loss↓				
[12]	*Persicaria capitata* (Buch.-Ham. ex D.Don) H.Gross	inflammatory cell, disordered arrangement of glands↓	IL-1β, IL-18, pro-IL-1β↓, NLRP3, pro-caspase-1↓			NF-kB↓, AKT, p-AKT↑
[13]	*Parnassia palustris* L	erosion, ulceration, hemorrhage, congestion↓; mucosa thickness repairability↑	IL-8, TNF-α↓, IL-2↑			
[14]	(Bamboo salt)	gastric damage and inflammation ↓	IL-1β, TNF-α↓			
[15]	(Korean Propolis)	gastric epithelium damage, inflammatory cell, sub-mucosal edema↓; PAS + cells↑	IL-1β, IL-8, TNF-α↓, NO, iNOS↓, A20, A1a, c-Myc↓		,	p-IκBα, p-p65↓
[17]	*Juglans regia *L	24w: gastric edema, erythema, inflammation, erosions, ulcer, atrophy↓ 36w: gastric tumor, ulcer, pale and thin mucosa, edema↓	IL-6↓, COX-2/COX-1, PGE_2_↓, c-Fos, c-Jun↓, SOCS-1↑, 15-PGDH↑	Keap1↓, NRF2, HO-1↑	c-Myc↓, Ki-67↓	NF-κB, p-p65↓, STAT3, pSTAT3↓
[18]	*Capparis zeylanica* L	gastric ulcer, petechial marks, hemorrhages, erosion, inflammation↓				
[19]	*Rubus crataegifolius* Bunge, *Ulmus macrocarpa* Hance	gastric inflammation↓				
[20]	*Allium hookeri*	gastric lesions score↓				
[22]	*Rubus crataegifolius* Bunge, *Ulmus macrocarpa* Hance, *Gardenia jasminoides* J. Ellis	gastric inflammation↓	iNOS, COX-2↓			
[23]	*Angelica keiskei*	gastric inflammatory lesions↓	MPO, IFN-γ↓, iNOS, COX-2↓	LPO↓		NF-κB activated B cells↓, IκBα↑
[24]	*Brassica rapa* L	gastric infiltration of eosinophils↓				
[25]	*Chenopodium ambrosioides* L	gastric inflammation↓				
[26]	*Polygonum capitatum*	pathological score↓	IFN-γ↓, IL-4↑		G cells, D cells in MALT↓	
[27]	(Licorice)	gastric erosion/ulcer, inflammation↓; dysplasia, tumor/adenoma↓	IL-1β, IL-6, IL-8, TNF-α↓, iNOS, COX-2, PGE_2_↓, bFGF, FcrRIIB, ICAM-1, Lungkine, Thymus-CK1, TRANCE, TROY↓	MDA↓	BrdU + cells↓	p-STAT3, pJAK2↓
[28]	× *Raphanobrassica karpechenkoi*	gastric mononuclear cells, heterotopic proliferation glands, intestinal metaplasia↓		8-OHdG↓	Ki-67 + cells↓	
[29]	*Vitis rotundifolia* Michx	gastric inflammation↓	IL-1β, TNF-α, IFN-γ↓			
[30]	*Malus domestica* cv Granny Smith	gastritis score↓		MDA↓		
[31]	(Garlic)	chronic atrophic gastritis, low-grade dysplasia, gastric intraepithelial neoplasia↓				
[32]	*Pistacia lentiscus* var. chia Poir	activity of chronic gastritis: gastric neutrophil infiltration, Sydney system score ↓				
[33]	(Aqueous rice)	stomach neutrophils, mucosa thickness↓			BrdU labeling index↓	
[34]	*Prunus mume* Sieb. et Zucc	stomach inflammatory cells, hemorrhagic erosion, mucosa hyperplasia↓				
[35]	*Calophyllum brasiliense* Cambess	stomach ulcer area, gastric inflammation, erosion↓				
[36]	(Grape, Green tea)	gastritis score↓				
[37]	*Artemisia capillaris* Thunb	24w: 1.gastric erosions, erythematous gastric mucosa, nodular mucosal changes, protuberant foci↓ 2.pathology score of CAG (loss of parietal cells, monocytes, lymphocytes, macrophages replacing gastric glands, and erosive mucosal changes)↓ 36 weeks: 1.gastric nodular mucosal changes, thinned gastric mucosa, adenomatous polyps, tumorous lesion with central ulcerations↓ 2.severe CAG, gastric ulcer, gastritis cystica profunda, adenoma, gastric adenocarcinoma↓	IL-1β, IL-6, TNF-α↓, COX-2, PGE_2_↓, Gastric F4/80 protein↓, 15-PGDH↑, HSP70↑	MDA↓		p-p65↓, pSTAT3↓
[38]	(Green tea)	inflammation, hyperplasia, dysplasia↓				
[39]	*Allium sativum* L	gastric edema, hemorrhage, hemorrhagic spots, gastric mucosa thickening↓				
[40]	*Oryza sativa* L	gastric neutrophils, mono cell↓			BrdU + cells↓	
[41]	*Alpinia officinarum* Hance	gastric inflammatory cell↓	IL-1β, IL-17, TNF-α↓			p-ERK1/2, p-JNK, p-p38↓
[42]	(Mastic)	gastric neutrophils, atropy↓				

CLO or RUT are test kits that could determine Helicobacter infection score by color. Three extracts of *Maclura tricuspidata*, Korean Propolis, *Allium hookeri*; and three compounds, *Chaenomeles speciosa* total triterpenoids, β-caryophyllene, and Phytoncide decreased CLO score. Seven extracts of *Corydalis yanhusuo* (Y.H.Chou & Chun C.Hsu), *Parnassia palustris* L., *Capparis zeylanica* L., *Tephrosia maxima.* L., *Chenopodium ambrosioides* L., *Calophyllum brasiliense* Cambess*.*, and *Alpinia officinarum* Hance; and one compound Epiberberine declined score of RUT.

Helicobacter Colony-Forming Unit (CFU) is a common way to quantitatively measure the severity of infection using stomach tissue. Seven extracts of *Persicaria capitata*, *Rubus crataegifolius* Bunge, *Ulmus macrocarpa* Hance, *Tephrosia maxima.* L., *Brassica rapa* L*.*, *Polygonum capitatum*, and *Pistacia lentiscus* L.; and five compounds, Baicalin, Sulforaphane, Neutral corn protein hydrolysate, Coptisine, and Linolenic Acid-Metronidazole significantly reduced the number value of CFU in experiment groups.

Urease is an important virulence factor that is essential for bacterial survival. Three extracts of *Punica granatum* L., *Brassica rapa* L., and *Prunus mume* Sieb. et Zucc. and two componds, Curcumin and Auraptene made urease levels decrease, thereby ameliorating infection.

Multiple plants improved infection conditions because they relieved Helicobacter gene expression in the host body. Two extracts of Bamboo salt, and Korean Propolis and one compound Phytoncide markedly down-regulated gene expression of the cytotoxin-associated gene A (*CagA*), which is the representative pathogenic factor. Two extracts of Red Wine, and Green Tea and two compounds, Baicalin and Geniposide, declined another key virulence gene vacuolating cytotoxin A (*VacA*) expression. Further, Korean Propolis extracted by ethanol attenuated gene expression of several Helicobacter pathogenic agents in tissues including *16S rRNA*, Sydney strain 1 (*Ss1*), encoding urease A subunit (*UreA*), surface antigen gene (*SsA*), and neutrophil-activating protein A (*NapA*). The gastric neuregulin 1 (*HrgA*) and *16S rRNA* gene expression were dampened by *Capparis zeylanica* L.. Curcumin could remove gastric tissue encoding urease B subunit (*UreB*) and *NapA* gene expression. Mastic extraction and *Sophora alopecuroides* L. total alkaloids significant reduced Helicobacter *16S rDNA* expression. In addition, *Angelica keiskei*, *Malus domestica* cv. Granny Smith; two compounds, β-caryophyllene and Eudesmin decreased Helicobacter *16S rRNA.*

Anti-Helicobacter antibodies such as IgG, IgA, and IgM, whose concentrations changed in animal tissue samples, also indicated the therapeutic effects of plants. Extracts of *Maclura tricuspidata*, *Corydalis yanhusuo* (Y.H.Chou & Chun C.Hsu), Korean Propolis, Aqueous rice, and *Oryza sativa* L., as well as compounds Phytoncide, Baicalin, and Plaunotol, lead to a reduction in anti-Helicobacter antibodies (Tables 1, 2, 3).

A few studies did not report results for Helicobacter eradication (“UN” on the right-most column), although they indeed treated animals with Helicobacter suspension and verified infection and gastritis. While these studies have drawbacks, they have irreplaceable research value because they concentrate on dredging the "inflammation‒cancer transition" mechanism. The corresponding mechanisms are shown in Tables 4 and 5.

**Table 5:** HAG-related regulatory mechanism of compound

Refs.	Bioactive Compound	Plant species	Gastric Histopathology	Infammatory Biomarkers	Oxidative Stress Biomarkers	Apoptosis OR Proliferation Biomarkers	Pathway Signature Molecule
[43]	2-hydroxybenzylamine	(Buckwheat)	acute and chronic inflammation, intramucosal carcinoma, dysplasia and carcinoma↓	IL-1β, IL-17, TNF, INF-γ, CXCL1↓, NOS2↓	pH2AX + x↓		
[44]	*Chaenomeles speciosa* total triterpenoids	*Chaenomeles speciosa* (Sweet) Nakai	gastric mucosal damage, inflammatory cell infiltration, glandular atrophy↓	MPO, IL-1β, IL-6, IL-18, KC, TNF-α, MCP-1↓, pro-ILs-1β, −18↓, TXNIP, NLRP3, pro-caspase-1, caspase-1↓, IL-4, IL-10↑	ROS, LDH, MDA↓, SOD, GSH-Px, CAT↑	Bax, Bad↓, Bcl-2, Bcl-xl↑, Bcl-2/Bax, Bcl-xl /Bad↑, cytochrome C, Apaf-1, PARP-1, cleaved-PARP-1, cleaved- caspases-3, −9↓, pro-caspases-3, −9↑	p-IKKβ, p-IκBα, p65, p-IKKβ/IKKβ, p-IκBα/IκBα↓, TLR4, MyD88↓
[45]	β-caryophyllene	*Syzygium aromaticum*	damage of the surface epithelium, inflammatory cell, submucosal edema↓	Gastric F4/80 protein↓			
[46]	Phytoncide	*Pinus koraiensis*	inflammation, atrophic score↓	IL-1β, TNF-α↓			
[47]	Baicalin, Baicalein	*Scutellaria baicalensis* Georgi		IL-1β↓			
[48]	Eudesmin	*Fatsia polycarpa* Hayata		IL-1β, IgM↓			
[49]	Geniposide, Genipin	*Gardenia jasminoides* J. Ellis		IL-1β, IFN-γ↓, COX-2↓, IgA, IgM↓			
[50]	Sulforaphane	*Brassica oleracea* L	gastric inflammation↓				
[51]	Quercetin	*Polygonum capitatum*		IL-8↓		G0/G1, G2/M phase cells↓, S-phase cells↑, Bax↓, Bcl-2↑	p38 MAPK↓
[52]	18β- Glycyrrhetinic Acid	*Glycyrrhiza glabra* L	gastric neutrophils, mononuclear cells, hyperplasia, peptic ulcer↓	IL-1β, TNF-α↓, COX-2, iNOS↓			
[29]	Quercetin	*Vitis rotundifolia* Michx	gastric inflammation↓	IL-1β, TNF-α, IFN-γ↓			
[54]	2–4 polymer urushiol	*Rhus verniciflua* Stokes	gastritis mitigation↓	IL-1β↓, TNF-α↑			
[55]	Arctigenin	*Arctium lappa* L	gastric intestinal metaplasia, heterotopic proliferative glands, incidences of glandular stomach adenocarcinoma↓		8-OHdG↓		
	Nordihydroguaiaretic acid	*Larrea tridentata* DC Coville					
[56]	Epiberberine	*Coptis chinensis* Franch	gastric tissue disturbances, cellular gaps, inflammatory cell↓				
[57]	Neutral corn protein hydrolysate	*Zea mays* L	gastric neutrophil, cell damage and swelling↓	MPO, IL-1β, IL-6, KC, TNF-α, MCP-1↓	MDA, LDH↓; SOD, GSH-Px↑		NF-κB↓, TLR4, MyD88↓
[58]	Coptisine	*Coptis chinensis* Franch		IL-2, IL-6↓			
[59]	Linolenic Acid-Metronidazole	(Flax, Soybeans, Rapeseed)	inflammatory cell infiltration↓				
[60]	Artemisinin, Artesunate, Dihydroartemisinin	*Artemisia annua* L	incidence and size of tumor nodules↓	IL-1β, IL-6, TNF-α↓, COX-2↓			p-IκBα ↓, IκBα↑
[61]	Curcumin	*Curcuma longa* L	gastric neutrophils, mononuclear cells, heterotopic proliferative glands↓	KC, IL-10↓			p-IκBα↓
	Capsaicin	*Capsicum* spp.		KC, TNF-α↓			
	Piperine	*Pipernigrum* L		IL-1β, IL-6, IL-10, KC, IFN-γ, TNF-α↓, iNOS↓			
[62]	Resveratrol	(Berries, Nuts, Peanuts, Grape skin)	gastric inflammation score↓	MPO, IL-8↓, iNOS↓	LPO↓		
[64]	Curcumin	*Curcuma longa* L	epithelial, submucosal and muscularis mucosal layers damage, inflammation, glandular atrophy↓				
[65]	Caffeic Acid Phenethyl Ester	(Propolis)	gastric neutrophils, mononuclear cells↓ intestinal metaplasia, heterotopic proliferative glands, hyperplasia↓	IL-2, IL-6, KC, TNF-α, IFN-γ↓, iNOS↓		BrdU + cells↓	p50, p-IκBα↓
[67]	Total secondary carotenoids	*Chlorococcum* Sp (microalgae)	gastric inflammation↓	IFN-γ↓, IL-4↑			
[68]	Plaunotol	*Croton stellatopilosus* H.Ohba	gastric inflammation↓				
[69]	*Sophora alopecuroides* L. total alkaloids	*Sophora alopecuroides* L	gastric inflammatory cell and proliferated glands↓	IL-8↓, COX-2↓			NF-κB↓
[70]	Palmatine	*Coptis chinensis* Franch	gastric inflammation↓	IL-8, ADAM17, HB-EGF, p-EGFR/EGFR, MMP-10, CXCL16, CD8 + T↓, Reg3a↑			

*Table 4 and 5 “↓ or ↑” indicates that the value is statistically significant compared with the control group

### Anti-inflammatory activity of plant extracts and compounds

Interleukin level is an important indicator against gastric inflammation. Overall, seven extracts derived from *Persicaria capitata,* Bamboo salt, Korean Propolis, Licorice, *Vitis rotundifolia* Michx.*, Artemisia capillaris* Thunb*.*, and *Alpinia officinarum* Hance; and sixteen compounds, 2-hydroxybenzylamine, *Chaenomeles speciosa* total triterpenoids, Phytoncide, Baicalin, Baicalein, Eudesmin, Geniposide, Genipin, 18β-Glycyrrhetinic Acid, Quercetin, 2–4 polymer urushiol, Neutral corn protein hydrolysate, Artemisinin, Artesunate, Dihydroartemisinin, and Piperine all reduced **IL-1β**. *Juglans regia* L., Licorice, *Artemisia capillaris* Thunb*.*, *Chaenomeles speciosa* total triterpenoids, Neutral corn protein hydrolysate, Coptisine, Artemisinin, Artesunate, Dihydroartemisinin, Piperine, and Caffeic Acid Phenethyl Ester alleviated the generation of **IL-6**. *Maclura tricuspidata*, *Parnassia palustris* L*.*, Korean Propolis, Licorice, *Chaenomeles speciosa* total triterpenoids, Quercetin, Neutral corn protein hydrolysate, Curcumin, Capsaicin, Piperine, Resveratrol, Caffeic Acid Phenethyl Ester, *Sophora alopecuroides* L*.* total alkaloids, and Palmatine revealed their capability of **IL-8** reduction in vivo. *Alpinia officinarum* Hance and 2-hydroxybenzylamine ameliorated the generation of **IL-17**, while *Persicaria capitata* and *Chaenomeles speciosa* total triterpenoids mitigated **IL-18** production. Some substances boosted IL levels, for example *Polygonum capitatum*, *Chaenomeles speciosa* total triterpenoids and Total secondary carotenoids upgraded **IL-4** levels in vivo. *Parnassia palustris* L. elevated **IL-2** content, whereas Coptisine and Caffeic Acid Phenethyl Ester reduced it. *Chaenomeles speciosa* total triterpenoids enhanced **IL-10** level, but Curcumin and Piperine significantly declined the production.

Seven plant-based extracts of *Parnassia palustris* L., Bamboo salt, Korean Propolis, Licorice, *Vitis rotundifolia* Michx., *Artemisia capillaris* Thunb., and *Alpinia officinarum* Hance, and eleven compounds *Chaenomeles speciosa* total triterpenoids, Phytoncide, 18β-Glycyrrhetinic Acid, Quercetin, Neutral corn protein hydrolysate, Artemisinin, Artesunate, Dihydroartemisinin, Capsaicin, Piperine, and Caffeic Acid Phenethyl Ester markedly diminished the levels of **TNF-α**. Moreover, researchers found that the following three extracts and six compounds decreased the interferon-gamma (**IFN-γ)** concentration in vivo: *Angelica keiskei*, *Polygonum capitatum*, *Vitis rotundifolia* Michx., 2-hydroxybenzylamine, Geniposide, Genipin, Piperine, Caffeic Acid Phenethyl Ester, and Total secondary carotenoids.

Multiple plant-derived medicines reduced isoform of nitric oxide synthase (**iNOS**)**/NO** containing Korean Propolis, *Rubus crataegifolius* Bunge, *Ulmus macrocarpa* Hance, *Gardenia jasminoides* J. Ellis, *Angelica keiskei*, Licorice, 2-hydroxybenzylamine, 18β-Glycyrrhetinic Acid, Piperine, Resveratrol, and Caffeic Acid Phenethyl Ester. As for another group of key inflammation-associated enzymes, cyclooxygenase-2/prostaglandin E_2_ (**COX-2**/**PGE**_**2**_), a variety of elements involving extracts of *Juglans regia* L., *Rubus crataegifolius* Bunge, *Ulmus macrocarpa* Hance, *Gardenia jasminoides* J. Ellis, *Angelica keiskei*, Licorice, *Artemisia capillaris* Thunb., Geniposide, Genipin, 18β-Glycyrrhetinic Acid, Artemisinin, Artesunate, Dihydroartemisinin, and *Sophora alopecuroides* L. total alkaloids have good efficacy on restraining **COX-2**/**PGE**_**2**_ generation in animal models. (Table 4, 5).

Table 4 and 5 summarize the mechanisms of plant products regulating HAG from four aspects: anti-inflammatory, anti-oxidative, anti-apoptosis and an-tiproliferation effects.

### Other findinds

**Fig. 8:**
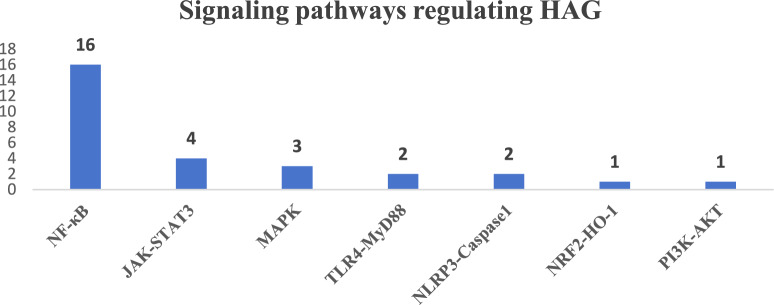
Signaling pathways regulating HAG

Major pathways regulating the HAG process have been exhibited on Fig. 8. The seven main signaling pathways regulating HAG are the nuclear factor kappaB (NF-κB), janus kinase-signal transducer and activator of transcription 3 (JAK-STAT3), mitogen-activated protein kinase (MAPK), toll-like receptor 4-myeloid differentiation factor 88 (TLR4-MyD88), NOD-, LRR- and pyrin domain-containing protein 3-caspase 1 (NLRP3-Caspase1), nuclear factor erythroid-2-related factor 2-heme oxygenase 1 (NRF2-HO-1), and phosphoinositide 3-kinase-protein kinase B (PI3K-AKT) pathways, which are critical mechanisms of these plant-derived substances. The NF-κB signaling pathway (16 elements) is the most thoroughly researched pathway and may be the most relevant one in HAG. (Fig. 8).

**Fig. 9:**
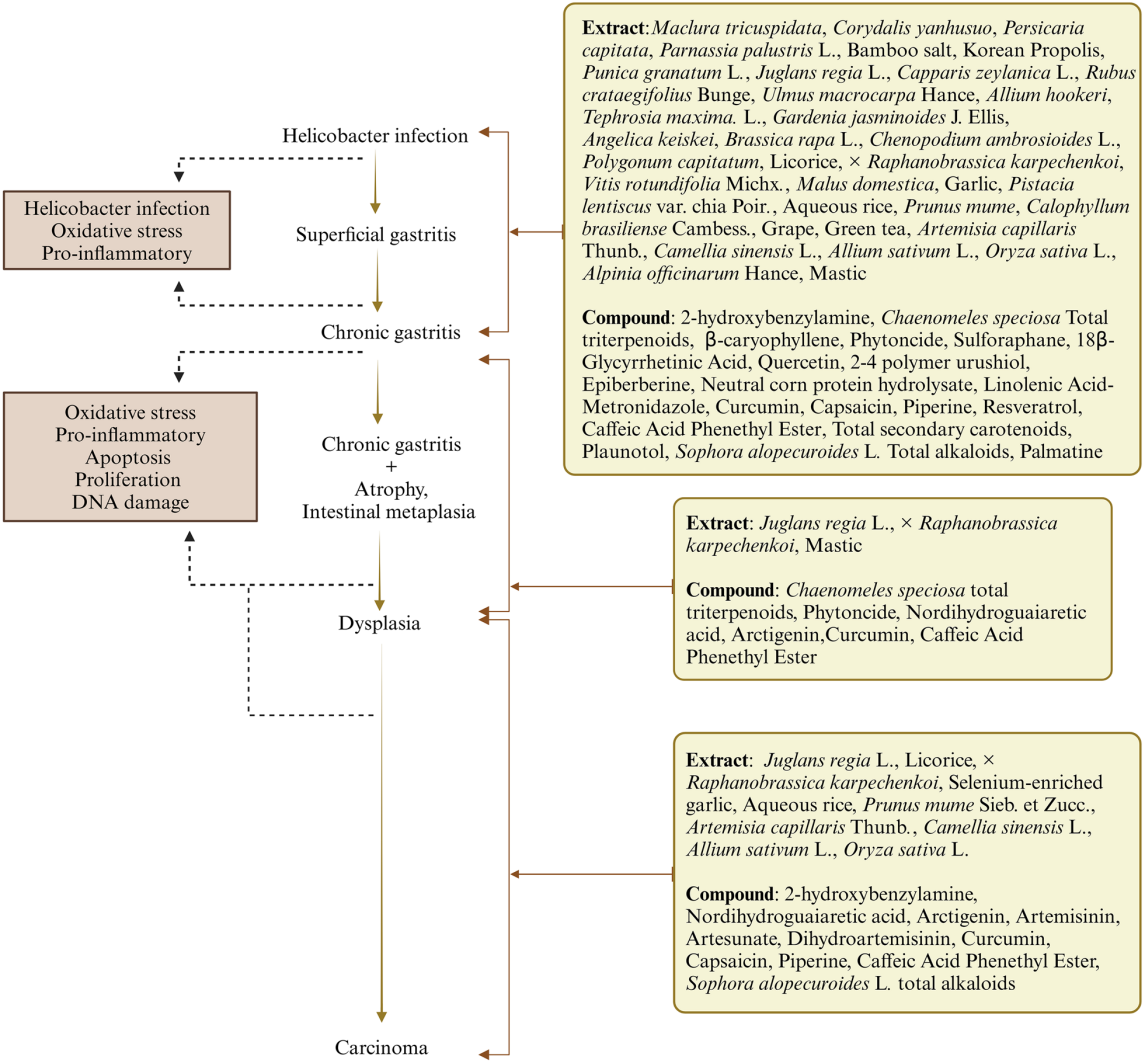
Phytomedicines act on Correa cascade. ( Created in BioRender. https://BioRender.com/daegq0t. Agreement number: OK28AFSX59)

In addition, several studies poked into the subsequent progress of HAG. As shown in Fig. 9, three extracts [17, 28, 42] and six compounds [44, 46, 55, 64, 65] effectively inhibit precancerous lesions. Ten extracts [17, 27, 28, 31, 33, 34, 37, 39, 40] and eleven compounds [43, 55, 60, 61, 65, 69] are significantly efficacious in suppressing stomach cancer. These plants or compounds unfold promising research prospects for treating advanced HAG lesions (Fig. 9).

**Fig. 10:**
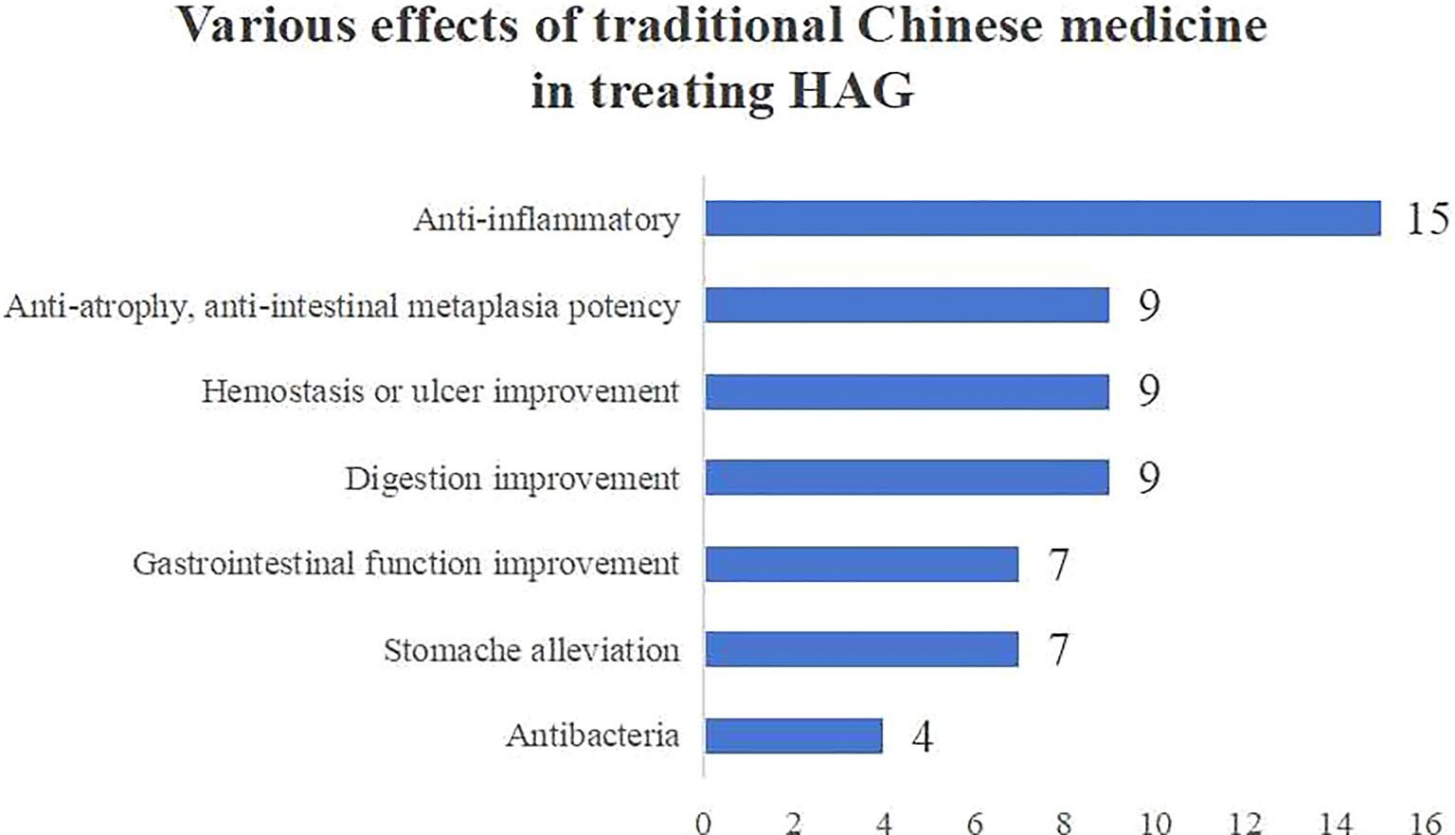
Various effects of traditional Chinese medicine in treating HAG

Aside from that, we discover that many of the plant medicines from the included literature are recorded in the Chinese Pharmacopoeia. Table 6 reveals their compound name, plant species, Chinese Pinyin, and the traditional function of Traditional Chinese Medicine (TCM) (Fig. 10 and Table 6).

**Table 6:** Traditional Chinese Medicine treating HAG

Refs	Compound OR Extract	Plant Species	Family	Chinese Pharmacopoeia Pinyin	Traditional Efficacy (TCM)
[26]	Extract	*Polygonum capitatum*	Polygonaceae	Huzhang (POLYGONI CUSPIDATI RHIZOMA ET RADIX)	Drain dampness and subdue yellowing, Clear heat and remove toxins, Transform stasis and alleviate pain, Stop coughing and transform phlegm
[51]	Quercetin				
[62]	Resveratrol				
[56]	Epiberberine	*Coptis chinensis* Franch	Ranunculaceae	Huanglian (COPTIDIS RHIZOMA)	Clear heat and drain dampness, Reduce fire and remove toxins
[58]	Coptisine				
[70]	Palmatine				
[22]	Extract	*Gardenia jasminoides* J. Ellis	Rubiaceae	Zhizi (GARDENIAE FRUCTUS)	Reduce fire and alleviate vexation, Clear heat and drain dampness, Cool blood and remove toxins
[49]	Geniposide, Genipin				
[27]	Extract	(Licorice)	Fabaceae	Gancao (GLYCYRRHIZAE RADIX ET RHIZOMA)	Tonify lung and supplement qi, Clear heat and remove toxins, Resolving phlegm and stop coughing, Resolving convulsion and alleviate pain
[52]	18β-Glycyrrhetinic Acid	*Glycyrrhiza glabra* L	Fabaceae		
[31]	Freeze-dried powder	(Garlic)	Amaryllidaceae	Dasuan (ALLII SATIVI BULBUS)	Remove toxins and resolve swelling, Kill parasites, Stop diarrhoea
[39]	Extract	*Allium sativum* L			
[61] [63] [64]	Curcumin	*Curcuma longa* L	Zingiberaceae	Jianghuang (CURCUMAE LONGAE RHIZOM)	Circulate blood and qi, Unblock meridians and relieve pain
[60]	Artemisinin, Artesunate, Dihydroartemisinin	*Artemisia annua* L	Asteraceae	Qinghao (ARTEMISIAE ANNUAE HERBA)	Clear deficiency heat, Clear bone-steaming tidal fever, Clear summer heat, Stop Malaria, Relieve yellowing
[47]	Baicalin, Baicalein	*Scutellaria baicalensis* Georgi	Lamiaceae	Huangqin (SCUTELLARIAE RADIX)	Clear heat and drain dampness, Reduce fire and remove toxins, Stop bleeding, Quiet the fetus
[55]	Arctigenin	*Arctium lappa* L	Asteraceae	Niubangzi (ARCTII FRUCTUS)	Release the exterior with pungent-cool, Disperse the lung and promote skin eruption, Remove toxins and clear the throat
[11]	Extract	*Corydalis yanhusuo*	Papaveraceae	Yanhusuo (CORYDALIS RHIZOMA)	Circulate blood, Resolve stagnation, Alleviate pain
[16]	Extract	*Punica granatum* L	Lythraceae	Shiliupi (GRANATI PERICARPIUM)	Astringe the intestine and stop diarrhoea, Stop bleeding, Repel parasitic worms
[17]	Shelled kernels pellet	*Juglans regia* L	Juglandaceae	Hetaoren (JUGLANDIS SEMEN)	Tonify the kidney, Warm the lung, Moisten the intestine
[34]	Extract	*Prunus mume* Sieb.et Zucc	Rosaceae	Wumei (MUME FRUCTUS)	Astringing lung and intestine, Generate fluids, Repel roundworms
[37]	Extract	*Artemisia capillaris* Thunb	Asteraceae	Yinchen (ARTEMISIAE SCOPARIAE HERBA)	Relieve dampness and heat, Promote bile flow and relieve yellowing
[40]	Distilled and enzyme-catalyzed product	*Oryza sativa* L	Poaceae	Daoya (ORYZAE FRUCTUS GERMINATUS)	Promote digestion and harmonize the middle jiao, Strengthen the spleen
[41]	Extract	*Alpinia officinarum* Hance	Zingiberaceae	Gaoliangjiang (ALPINIAE OFFICINARUM RHIZOMA)	Warm the stomach and stop vomiting, Dissipate cold and alleviate pain
[44]	*Chaenomeles* *speciosa* total triterpenoids	*Chaenomeles speciosa* (Sweet) Nakai	Rosaceae	Mugua (CHAENOMELIS FRUCTUS)	Relax tendons and harmonize meridians, Harmonize the middle jiao and transform dampness
[53]	Epimedium polysaccharide	UN	Berberidaceae	Yinyanghuo (EPIMEDII FOLIUM)	Warm kidney yang, Strengthen tendons and bones, Eliminate wind and resolve dampness
[53]	Astragalus polysaccharides	UN	Fabaceae	Huangqi (ASTRAGALI RADIX)	Tonify qi and promote yang energy, Secure the exterior and stop sweating, Induce urination and resolve oedema, Generate fluids and nourish blood, Resolve stagnation and unblock impediment, Remove toxin and drain the pus, Astringe wound and regenerate new tissues
[54]	2–4 polymer urushiol	*Toxicodendron vernicifluum* (Stokes) F. A. Barkley	Anacardiaceae	Ganqi (TOXICODENDRI RESINA)	Transform stasis and unblock meridians, Alleviate malnutrition and kill parasitic worms
[61]	Capsaicin	*Capsicum annuum* L	Solanaceae	Lajiao (CAPSICI FRUCTUS)	Warm the middle jiao and remove cold, Promote digestion
[61]	Piperine	*Piper nigrum* L	Piperaceae	Hujiao (PIPERIS FRUCTUS)	Warm the middle jiao and remove cold, Regulate qi, Transform phlegm
[65]	Caffeic Acid Phenethyl Ester	UN	UN	Fengjiao (PROPOLIS)	Invigorate the weak, Transform turbidity, Relieve wasting thirst disorder
[66]	Auraptene	*Citrus* × *aurantium* f. *aurantium*	Rutaceae	Zhishi (AURANTII FRUCTUS IMMATURUS); Zhiqiao (AURANTII FRUCTUS)	Zhishi: Circulate qi and resolve masses, Transform phlegm and resolve masses; Zhiqiao: Circulate qi and harmonize the stomach, Resolve stagnation and promote digestion

**Fig. 11:**
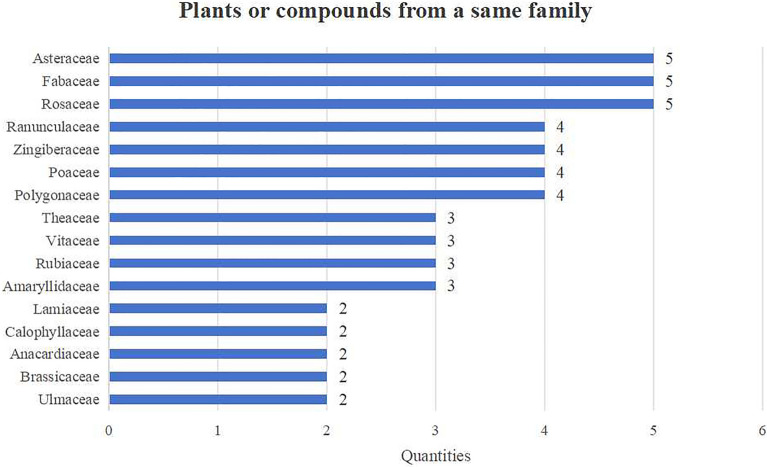
Plants or compounds from a same family

Many plant extracts or compounds belong to sixteen main families. The top three families with the highest frequency (five quantities) are Asteraceae, Fabaceae and Rosaceae (Fig. 11).

**Fig. 12:**
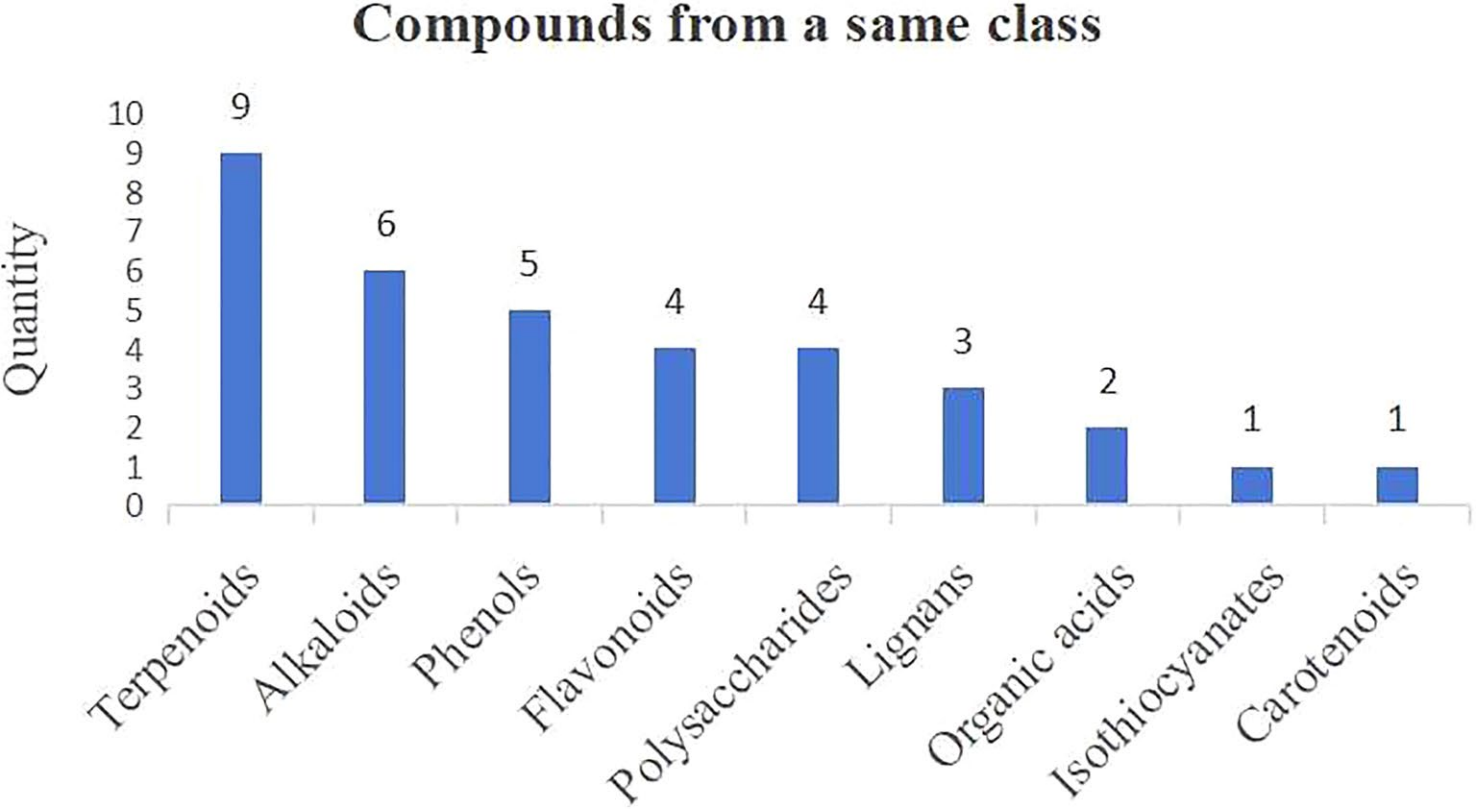
Compounds from a same class

In addition, many plant-derived compounds from the same classes, such as Terpenoids and Flavonoids. Figure 12. displays the frequency of each important class and their ranking. The compounds mainly attribute to nine classes, with Terpenoids being the largest class, which contains nine compounds, Alkaloids, and Phenols, each comprising six and five compounds, respectively (Fig. 12).

## Discussion

The meta-analysis shows that plant-derived extracts and compounds possess anti-Helicobacter and anti-inflammatory efficacy to treat HAG. Animal experiments illustrate that phytomedicine decreased Helicobacter positive rates of CLO and RUT while reducing levels of IL-1β and TNF-α. These phytomedicines improve HAG and block disease progression by regulating several mechanisms, including anti-Helicobacter, anti-inflammatory, anti-oxidative, anti-apoptotic, and anti-proliferative, through multiple signaling pathways including NF-κB, JAK2/STAT3, MAPK, TLR4/MyD88, PI3K/AKT, NLRP3/Caspase-1, and NRF2/HO-1. We also found that TCM demonstrates enormous potential for treating HAG because of its comprehensive and many-sided therapeutic effects on HAG, including anti-inflammatory, antibacterial, anti-atrophy, anti-intestinal metaplasia, hemostasis or ulcer improvement, digestion improvement, gastrointestinal function improvement, and stomach alleviation.

### Mechanisms of Helicobacter pathogenicity in HAG

The pathogenic mechanisms of Helicobacter are related to its colonization, survival, and virulence factors, which cause an inflammatory response, oxidative stress, and progressive epithelial lesions of the stomach.

**Fig. 13:**
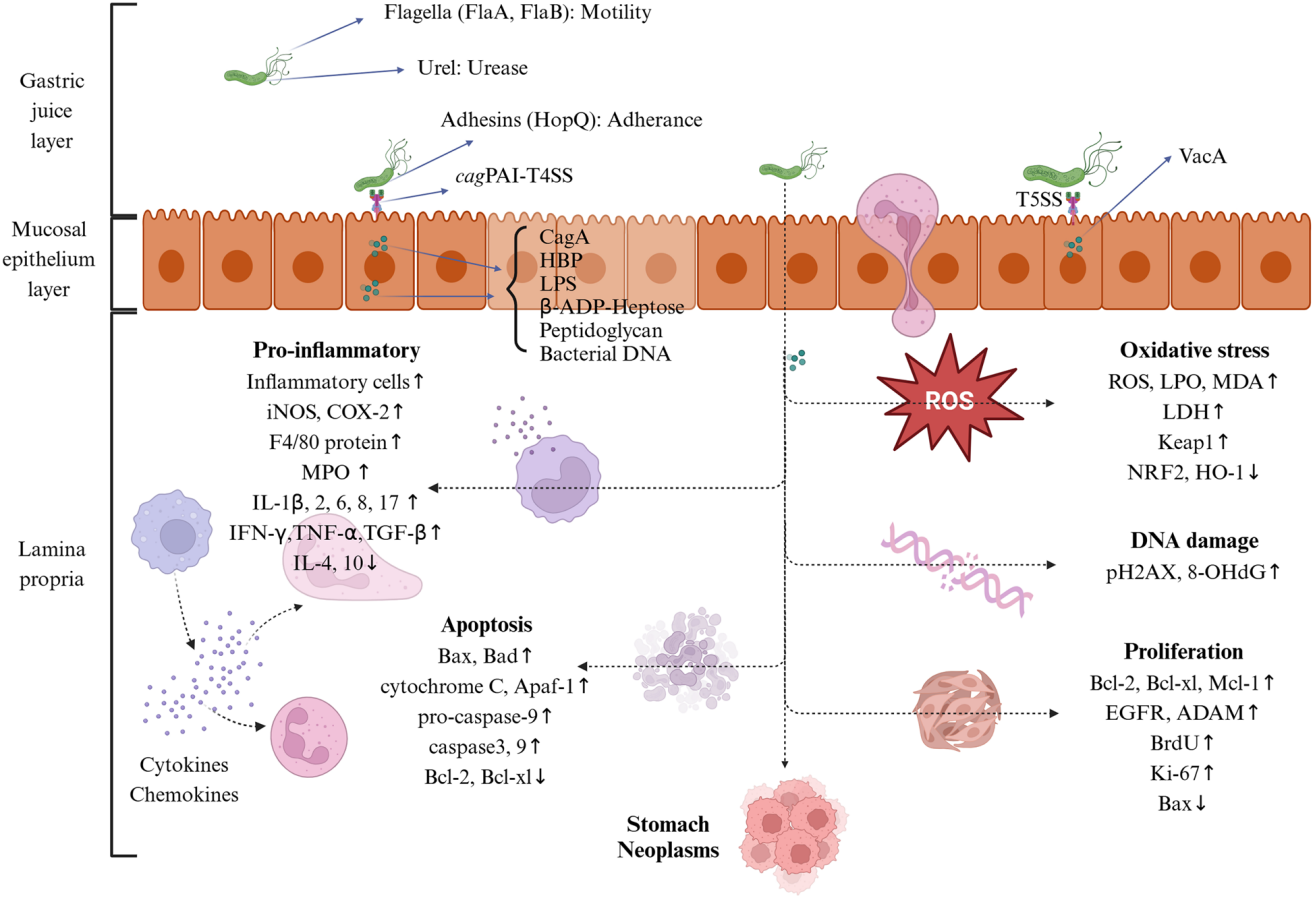
Mechanisms of HAG injuries. (Created in BioRender. https://BioRender.com/o86q708. Agreement number: GD27RQB2OM.) Helicobacter infects and survives in the stomach via various pathogenic factors. Helicobacter causes damage to the host and ultimately causes tumors via oxidative stress, inflammation, DNA damage, apoptosis, and proliferation ways. The order in which mechanisms are listed in the figure does not represent their order of occurrence in diseases. HopQ, Hop family adhesin HopQ; *cag*PAI, cytotoxin-associated gene pathogenicity island; T4SS, type IV secretion system; CagA, cytotoxin-associated gene A protein; HBP, heptose-1, 7-bisphosphate; T5SS, type V secretion system; VacA, vacuolating cytotoxin A; iNOS, isoform of nitric oxide synthase; COX-2, cyclooxygenase-2; MPO, myeloperoxidase; IL, interleukin; IFN-γ, interferon-gamma; TNF-α, tumor necrosis factor-α; TGF-β, transforming growth factor-β; Bax, Bcl-2-associated X protein; Bad, Bcl-2-associated agonist of cell death; Apaf-1, apoptotic protease activating factor-1; Bcl-2, B-cell lymphoma-2 protein; Bcl-xl, Bcl-2-like protein-1; ROS, reactive oxygen species; LPO, lipid peroxide; MDA, malondialdehyde; LDH, lactatedehydrogenase; Keap1, kelch-like ECH-associated protein 1; NRF2, nuclear factor erythroid-2-related factor 2; HO-1, heme oxygenase-1; pH2AX, phospho-histone H2A. X; 8-OHdG, 8-hydroxydeoxyguanosine; Mcl-1, myeloid cell leukemia protein 1; EGFR, epidermal growth factor receptor; ADAM, a disintegrin and metalloproteinase; BrdU, 5’-bromodeoxyuridine; Ki-67, antigen identified by monoclonal antibody Ki-67.

Motility, urease, and adhesion are three common Helicobacter pathogenic mechanisms. Helicobacter colonization ability relies on motility, urease activity, and adhesion. The **motility** of Helicobacter, owing to bacterial-sheathed flagella with filaments consisting of two flagellin subunits [71] (FlaA and FlaB), which prevent the activation of the host innate immune system via escape recognition by TLR5 [72, 73], is indispensable for bacterial entry into the mucus. The ability of bacteria to adapt chemotactically relies on the pH of the gastric mucus [74]. **Urease** is essential for Helicobacter colonization; it decomposes urea into ammonia and carbon dioxide, which enables bacteria to survive at very low pH values. The large amount of urease that Helicobacter produces is aided by Urel, which is an acid-stimulated inner membrane protein. **Adhesion** ability is inseparable from adhesins, proteins anchored on the bacterial outer membrane, which are encoded by members of the large hop superfamily of outer membrane protein-encoding genes. *SabA* [75], Hop family adhesin BabA (*BabA*) [76], Hop family adhesin AlpA (*HopC*), Hop family adhesin AlpB (*HopB*) [77], and Hop family adhesin HopQ (*HopQ*) are crucial gene for adhesins of Helicobacter. HopQ is a key adhesin that combines with human carcinoembryonic antigen-related cell adhesion molecules (CEACAMs), thereby translocating the major pathogenicity factor CagA into cells [78, 79]. Pathogenic feature genes of Helicobacter major are present in pathogenic island. The cytotoxin-associated gene A protein (CagA) and vacuolating cytotoxin A protein (VacA) are responsible for stomach tissue inflammation and damage by activating NF-κB [80, 81]. Helicobacter susceptibility and widespread prevalence are due to the acquisition of cytotoxin-associated gene pathogenicity island (c*ag*PAI), which encodes the type IV secretion system (T4SS). The T4SS, a protein complex spanning the bacterial cell envelope, can directly deliver various effector molecules, including the proinflammatory and oncogenic protein CagA [82], HBP (heptose-1, 7-bisphosphate, an essential intermediate metabolite of the lipopolysaccharide inner heptose core) [83, 84], peptidoglycan fragments [85], and bacterial DNA [86], into host cells after bacterial adherence. These bacterial substances interact with intracellular target molecules and have substantial effects on processes such as intracellular signaling, cell function, and even malignant transformation in the host [87, 88]. Multiple studies have confirmed that c*ag*PAI-positive strains trigger more inflammation than negative strains do [78, 83, 89]. In addition, the CagA protein, transcribed by the *CagA* gene, which includes two critical motifs, *EPIYA* and *CRPIA*, accounts for the high expression of proinflammatory cytokines (IFN-γ, IL-1β [90], and IL-8 [91]), DNA damage [92], gastric epithelial cell apoptosis, and gastric adenocarcinoma. VacA is released via the type V secretion system (T5SS) and enters host cells through endocytosis. Its transport to mitochondria results in cell apoptosis via mitochondrial transmembrane potential (ΔΨm) dissipation, cytochrome c release, and Bcl-2*-*associated X protein (Bax) activation [93]. (Fig. 13 and Table 1, 2).

### Mechanisms of inflammatory regulation in HAG

Host inflammation is the primary and most vital aspect of HAG. Once monocytes, macrophages, and epithelial cells identify damage-associated molecular patterns (DAMPs) or microbial-associated molecular patterns (MAMPs), inflammation ensues. These cells secrete proinflammatory cytokines and chemokines. The network that builds connections between cells and cytokines in the immune system facilitates responses to Helicobacter infection. The host inflammatory factors ILs-1β, −2, −4, −6, −8, −10, −17, and −18, IFN-γ, and TNF-α are closely associated with HAG and, even later, GC.

The **F4/80 protein** content in stomach tissue slices indicates the extent of macrophage infiltration. ***Artemisia capillaris* Thunb.** and **β-caryophyllene** decreased the levels of F4/80. In addition, myeloperoxidase **(MPO)** is a heme protein that neutrophils aggregate and release if stimulated. MPO content can indicate the activation and infiltration of neutrophils. In included studies, one extract (***Angelica keiskei****)* and three compounds (***Chaenomeles speciosa* total triterpenoids**, **Neutral corn protein hydrolysate**, and **Resveratrol**) decreased MPO levels.

#### Pro-inflammatory cytokines in HAG

Lipopolysaccharide (LPS) of Helicobacter induces **IL-1β** [94]. Comparing to *Il-1β (*+ */* +*)* mice, *Il-1β (-/-)* mice exhibited attenuated inflammatory cell recruitment, proliferation excess, and apoptotic deficiency to inhibit gastritis and carcinogenesis [95]. **ILs-6**, **−8**, **−11**, and **−17** are cytokines strongly linked with HAG-to-cancer progression. Strains expressing CagA strongly activate extracellular signal-regulated kinase 1/2 (ERK1/2), STAT3, and increase IL-6 and −11 levels, which results in the aggravating of HAG and gastric cancer [96]. IL-6 significantly elevates in GC patients and is positively correlated with C-reactive protein (CRP) level, tumor size, stage [97], invasion, lymph node, and hepatic metastasis [98], and survival time [99]. *Il-8* is the most selective and consistent gene in HAG patients [100], and it is the most up-regulated gene according to whole-genome profiling of Helicobacter-exposed gastric epithelial cells [101]. Helicobacter induces epithelial gastric cells or cancer-derived cell lines to generate elevated levels of IL-8 via the activation of activator protein 1 (AP-1), NF-κB [102, 103], or STAT3 [104]. Additionally, a high level of IL-8 is strongly related to venous and lymphatic invasion and invasion depth [105]. Keratoconus (KC) is a rodent homolog of human IL-8. Likewise, Helicobacter stimulates IL-17 and IFN-γ in mice and increases IL-23 and IL-12 in macrophages. Anti-IL-17 Ab-treated *Il-17 (-/-)* mice have a reduced bacterial load and gastric inflammation, whereas recombinant adenovirus, which encodes mouse IL-17, exacerbates gastritis [106].

The T-helper 1 (Th1)-mediated factor IFN-γ, which is secreted mainly by CD4 + and CD8 + T cells [107, 108], is closely correlated with the severity of Helicobacter-induced inflammation in the stomach [108–110]. After eradication therapy, the level of IFN-γ decreased to the same level as that in the uninfected group [111]. The level of TNF-α is always significantly increased with IFN-γ and IL-12 in HAG patients [112], mice and in vitro.

#### Anti-inflammatory cytokines in HAG

Some protective cytokines in HAG have anti-inflammatory functions. The tissue isolated from infected human stomach mucosa and mice showed a prevalence of IFN-γ-producing T cells, whereas IL-4-producing T cells were rare or absent [109, 112–114]. Infected *Il-4 (-/-)* mice had increased IFN-γ level and more severe gastritis. On the other hand, *Ifn-γ (-/-)* mice showed no inflammation but high IL-4.

Tiny amounts of IL-10 were detected when the T cells were stimulated with Helicobacter urease in vitro [107]. Another study revealed that live Helicobacter induced IL-12 and IFN-γ tens of times, whereas IL-10 slightly increased. Interestingly, compared with live Helicobacter, killed Helicobacter induced significantly more IL-10. It demonstrated that live Helicobacter induced Th1 cells, which produced IL-12 and IFN-γ, whereas oral vaccines may induce more IL-10 [115]. Therefore, IL-4 and IL-10 function as protective factors in gastritis.

In the included studies, three studies reported a significant increase in IL-4 level: ***Polygonum capitatum***, ***Chaenomeles speciosa* total triterpenoids**, **and Total secondary carotenoids of *Chlorococcum* Sp*.*** However, only one study showed a significant increase in IL-10: ***Chaenomeles speciosa* total triterpenoids**. IL-10 had no significant decrease change comparing to model groups after the following treatments: × ***Raphanobrassica karpechenkoi*** (↓no sig), ***Calophyllum brasiliense* Cambess.** (↓no sig), **Caffeic acid phenethyl ester** (↓no sig). **Curcumin** and **Piperine** decreased the concentration of IL-10 significantly due to a decrease in monocytes in the lamina propria during inflammatory rehabilitation.

#### Inflammation-related enzymes in HAG

Nitric oxide synthase (**NOS**), which is created by L-arginine in response to Helicobacter infection, consists of three distinct NOS isoforms. One of the isoforms, **iNOS**, a calcium-independent isoform, responds to bacterial LPS and proinflammatory cytokines. iNOS creates a significant amount of NO when injurious stimuli occur in cells. TNF-α, IFN-γ, IL-1β, and LPS attach to receptors on the cell membrane, activating NF-κB and STAT, which translocate into the nucleus and finally start *iNOS* gene transcription [116]. The levels of iNOS, COX-2, and NO [100] are greater in Helicobacter-positive gastritis patients, especially in the bacterial density of the gastric antrum [117].

iNOS and **COX-2** are associated with GC [118]. Tumor-associated macrophages (TAMs) with high COX-2 accumulate near GC tumor nests. COX-2 and iNOS catalyze the increase in **PGE**_**2**_ and **NO**, respectively, in gastric cancer. The long-lasting effects of NO and PGE_2_ lead to oxidative stress, DNA damage, and the overexpression of DNA methyltransferases [119].

The tumor suppressor enzyme 15-prostaglandin dehydrogenase (**15-PGDH**) is a critical PG catabolic enzyme. Early inactivation of 15-PGDH causes COX-2 activation and contributes to PGE_2_ overproduction, which leads to colon carcinogenesis. Hence, the loss of 15-PGDH increases PGE_2_ in gastric-intestine cancer [120, 121]. ***Juglans regia* L.** and ***Artemisia capillaris* Thunb*.*** effectively preserved 15-PGDH in infected mice with a decrease in COX-2/COX-1 and PGE_2_, which restrained the tumor on stomach. It explains how it blocks the “inflammation‒carcinoma” process. (Tables 4, 5).

### Antioxidative effects and mechanisms in HAG

The Helicobacter colonizing mucosa undergoes remarkable neutrophil infiltration and oxyradical formation, which cause damage, including erythema, ulcers, and hemorrhage. When the body's oxidative stress and antioxidant processes are out of balance, inflammation, overapoptosis, and overproliferation are promoted.

Reactive oxygen species (**ROS**), which produce oxygen with electrons**,** are crucial factors for polyunsaturated fatty acid peroxidation of the cell membrane. When injuries occur, ROS and reactive nitrogen species (RNS), which are generated by Helicobacter and activated neutrophils, serve as chemoattractants that attract more neutrophils and monocytes. Additionally, ROS and RNS cause DNA damage that fuels tumor growth [122]. ***Chaenomeles speciosa* total triterpenoids** significantly reduced ROS level in infected group.

Lipid peroxide (**LPO**) radicals are converted from lipid-free radicals generated by ROS-oxidizing polyunsaturated fatty acids. Hence, LPO is considered an index of oxidative membrane damage [23]. Superoxide dismutase (**SOD**), catalase (**CAT**), glutathione peroxidase (**GSH-Px**), glutathione (**GSH**), and Vit C/E can effectively eliminate ROS. These antioxidants guard the gastric mucosa against superoxide anion damage. The level of ROS is increased in the mucosa of HAG patients, resulting in GSH depletion [123]; however, the level of ROS decreases after anti-Helicobacter treatment [124]. ***Angelica keiskei*** and **Resveratrol** ameliorated the generation of LPO. **Neutral corn protein hydrolysate** elevated the production of SOD and GSH-Px, and ***Chaenomeles speciosa* total triterpenoids** increased SOD, GSH-Px and CAT.

Lactatedehydrogenase (**LDH**) releases when Helicobacter stimulates intracellular NADPH oxidase to generate endogenous stress factors that assault the cell membrane and cause lipid peroxidation, thereby destroying the membrane [57]. ***Chaenomeles speciosa*** **total triterpenoids** and **Neutral corn protein hydrolysate** alleviated the up-regulation of LDH.

Malondialdehyde (**MDA**) is a biomarker of oxidative stress [125], the excessive accumulation of which causes cell membrane dysfunction. MDA increased whereas SOD decreased in a gastric mucosal damage model [126, 127]. Three plant extracts: **Licorice**, ***Malus domestica* cv. Granny Smith**, and ***Artemisia capillaris* Thunb*.***, and two compounds, ***Chaenomeles speciosa* total triterpenoids** and **Neutral corn protein hydrolysate**, significantly declined the production of MDA.

Phospho-histone H2A. X (**pH2AX**) marker, which is linked to the generation of reactive aldehydes and DNA damage, increases the number of nuclei in gastric epithelial cells in Helicobacter-infected mice. The **2-hydroxybenzylamine** decreased the number of pH2AX-positive cells in the mice. The 8-hydroxydeoxyguanosine (**8-OHdG**) is a marker of oxidative DNA damage. × ***Raphanobrassica karpechenkoi*** reduced the level of 8-OHdG in the gastric mucosa, and two compounds **Nordihydroguaiaretic acid** and **Arctigenin**, decreased the level of 8-OHdG in the serum (Tables 4, 5).

### Anti-apoptotic effects and mechanisms in HAG

Helicobacter colonization can destroy gastric mucosal barrier function, causing apparent cell apoptosis. Some plant substances decrease the occurrence of cell apoptosis, which can cause gastric lesions or peptic ulcer diseases. Zhang S et al. [51] found that Helicobacter infection elicited cell cycle arrest at the G1/S transition. The number of G0/G1-phase (DNA/DNA synthesis) cells significantly increased, but the number of S-phase (DNA synthesis) cells decreased. **Quercetin** [51] reduced the number of G0/G1-phase cells and increased the number of S-phase cells, therefore protecting the gastric mucosa and maintaining the balance between the loss and regeneration of epithelial cells.

The B-cell lymphoma gene 2 *(Bcl-2*) family consists of two subfamilies: proliferation agonists (*Bcl-xl*, *Bcl-2*, *Bcl-w*, *Brag-1*, *Bfl-1*, and *Mcl-1*) and apoptosis agonists (*Bcl-xs*, *Bax*, *Bad*, *Bid*, *Bak*, and *Hrk*) [128]. Bcl-2, BH3 interacting domain death agonist (Bid), and Bax increased in Helicobacter-infected gastric adenocarcinoma [129]. Bax increased during gastric epithelial barrier injury [130]. Bax/Bcl-2, which belongs to the mitochondrial apoptotic pathway, is a crucial ratio that modulates the balance between apoptosis and proliferation. Helicobacter-associated apoptosis may contribute to cell proliferation or gastric atrophy, resulting in GC [131]. Helicobacter colonization activated the p38 MAPK pathway to induce apoptosis, but **Quercetin** reversed this harmful effect by attenuating p38, IL-8 production, and the declining Bax/Bcl-2 ratio. ***Chaenomeles speciosa* total triterpenoids** exhibited anti-apoptotic potency, increasing Bcl-2-like 1 (Bcl-xl), Bcl-2, Bcl-xl/Bad, and Bcl-2/Bax while decreasing Bad, Bax.

Pro-apoptotic proteins Bax and Bad and inflammatory stimulation disrupt the integrity of the mitochondrial membrane, causing a decrease in the mitochondrial membrane potential, leading to the release of cytochrome C from the mitochondria into the cytoplasm and the activation of Apoptotic protease activating factor-1 (Apaf-1). Apaf-1, cytochrome C and pro-caspase-9 form apoptotic vesicles. The apoptotic vesicles cleave pro-caspase-3 and pro-caspase-9 into cleaved caspase-3 and −9 and ultimately cause apoptotic cell death. In addition, the superoxide produced by Helicobacter infection in the gastric mucosa causes poly ADP‒ribose polymerase-1 (PARP-1) activation and promotes the release and development of mitochondrial apoptosis-inducing factor (AIF). ***Chaenomeles speciosa* total triterpenoids** act as anti-apoptotic agents by the manner mentioned above that relieved levels of cytochrome C, Apaf-1, pro-caspase-9, and cleaved caspase-3 and −9, as well as PARP-1. (Table 4, 5).

### Anti-proliferative effects and mechanisms in HAG

CagA up-regulates the pro-survival factors phospho-ERK and Myeloid cell leukemia protein-1 (Mcl-1) in infected mice, interfering with host cell survival and anti-apoptotic processes that overcome epithelial self-renewal and help sustain Helicobacter infection [132]. Helicobacter-associated GC is associated with Bcl-2 up-regulation and Bax decline, which induces overproliferation [118]. Epidermal growth factor receptor (EGFR), which regulates epithelial cell differentiation, proliferation, and apoptosis [133], plays a crucial role in gastric cancer [134]. **Palmatine** reduced Heparin-binding epidermal growth factor-like growth factor (HB-EGF) and p-EGFR/EGFR levels, suppressing HAG progression.

**Myc** proto-oncogene (*Myc/c-Myc)* is an active transcription factor that functions via transcriptional amplification of target genes to regulate cell differentiation and proliferation. Helicobacter-positive patients [135] and human gastric adenocarcinoma samples [136] have increased *Myc* expression. Two extracts of **Korean Propolis** and ***Juglans regia* L**. significantly decreased *c-Myc* among the included studies.

The cells that undergo DNA synthesis (in the S-phase of the cell cycle) during exposure to **BrdU** (5’-bromodeoxyuridine) in the stomach glands will be labeled with BrdU and counted. BrdU incorporation signifies that cellular proliferation occurs at positions such as the base of the gastric gland and the apoical portion. Four studies among the included studies revealed that **Caffeic acid phenethyl ester**, **Licorice**, **Aqueous rice**, and ***Oryza sativa* L.** alleviated inflammation and decreased the hyperplasia score (BrdU-positive cells) in animals. Moreover, **Ki-67**-positive cells, which are detected across the hyperplastic mucosa in Helicobacter-infected mice, are a marker of the proliferative index. × ***Raphanobrassica karpechenkoi*** and ***Juglans*** ***regia*** ***L.*** reduced the number of Ki-67-positive cells. (Table 4, 5).

### Signaling pathways modulate HAG

#### NF-κB

The NF-κB family members p50, p65 combine to form homodimers and heterodimers which are retained in the cytosol by interacting with inhibitors (IκBs). When stimuli such as oxidative stress and inflammation are present, IκB is phosphorylated by the IκB kinase (IKK) complex, which results in the IκB/p50/p65 complex isolating from IκB to translocate to the nucleus, bind to specific genes, and subsequently lead to the release of inflammatory factors. Peptidoglycan, which is encoded by *cag*PAI and recognized by nucleotide-binding oligomerization domain-containing protein 1 (NOD1), enters host cells via the T4SS and activates NF-κB [85]. The virulence factors CagA [90, 137] and VacA induce NF-κB activation, causing the release of proinflammatory cytokines [93]. Aside from the NOD1 sensor, HBP, ADP-heptose/ALPK1-TIFA/NF-κB initiates initial inflammation, which occurs even earlier than NOD1 activation [138].

**Fig. 14:**
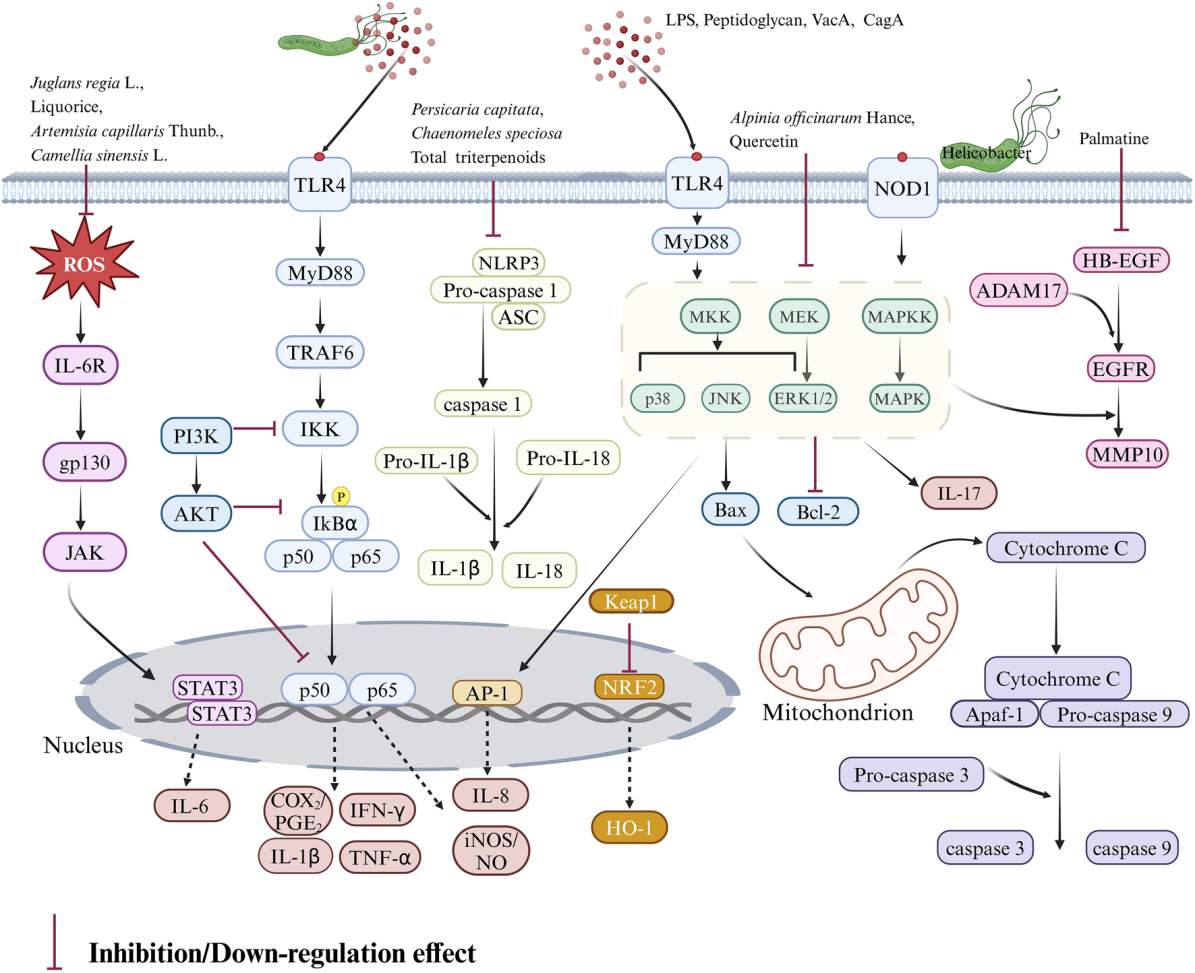
Signaling pathways regulating the HAG. (Created in BioRender. https://BioRender.com/m32b229. Agreement number: RT27ZDTNZS.) LPS, peptidoglycan, VacA, and CagA are bacterial fragments from Helicobacter, and these fragments cause the release of multiple factors that take part in inflammation, apoptosis, overproliferation, and tumors through different signaling pathways including NF-κB, JAK/STAT3, MAPK, TLR4/MyD88, PI3K/AKT, NLRP3/Caspase-1, and NRF2/HO-1. ROS, reactive oxygen species; IL-6R, interleukin 6 receptor; gp130, glycoprotein 130; JAK, janus kinase; STAT3, signal transducer and activator of transcription 3; PI3K, phosphoinositide 3-kinase; AKT, protein kinase B; TLR4, toll-like receptor 4; MyD88, myeloid differentiation primary response gene 88; TRAF6, tumor necrosis factor receptor-associated factor 6; IKK, IκB kinase; IκBα, inhibitor of kappa B; NLRP3, NOD-, LRR- and pyrin domain-containing protein 3; ASC, apoptosis associated speck-like protein containing a CARD; AP-1, activator protein 1; Keap1, kelch-like ECH-associated protein 1; NRF2, nuclear factor erythroid-2-related factor 2; HO-1, heme oxygenase-1; MKK, mitogen-activated protein kinase kinase; MEK, mitogen-activated extracellular signal-regulated kinase; MAPKK, MAP Kinase Kinase; MAPK, mitogen-activated protein kinase; ERK1/2, extracellular signal-regulated kinase 1/2; JNK, jun N-terminal kinase; LPS, lipopolysaccharides; CagA, cytotoxin-associated gene A protein; VacA, vacuolating cytotoxin A; NOD1, nucleotide-binding oligomerization domain-containing protein 1; HB-EGF, heparin-binding epidermal growth factor-like growth factor; ADAM17, a disintegrin and metalloproteinase 17; EGFR, epidermal growth factor receptor; MMP10, matrix metalloproteinase 10; Apaf-1, apoptotic protease activating factor-1; IL-1β, interleukin-1β; IL-6, interleukin-6; IL-8, interleukin-8; IL-17, interleukin-17; TNF-α, tumor necrosis factor α; IFN-γ, interferon γ; iNOS, inducible nitric oxide synthase; NO, nitric oxide; COX-2, cyclooxygenase-2; PGE_2_, prostaglandin E_2_.

***Persicaria capitata*** alleviated inflammatory cell infestation and improved the gland arrangement with IL-1β, IL-18, pro-IL-1, NLRP3, and pro-caspase-1 reduction through elevating PI3K/AKT but suppressing NF-κB. **Korean propolis** reduced p-IκBα and p-p65 levels, which decreased the levels of IL-1β, IL-8, TNF-α, iNOS, and NO. **Korean propolis** decreased tumor necrosis factor α-induced protein 3 (TNFAIP3 or A20), A1a, and *c-Myc*, which have a noticeable positive correlation with NF-κB [139, 140] and aggravate gastrointestinal inflammation. ***Juglans regia *L.** obviously improved HAG condition (edema, erythema, inflammation, ulcer) and subsequent disease deterioration (ulcer, atrophy, tumor, pale and thin mucosa), which accompanied lower production of IL-6, COX-2/COX-1, PGE_2_, c-Fos, c-Jun, c-Myc, p-p65, and pSTAT3. ***Angelica keiskei***, which has good anti-HAG ability, inhibits LPO, iNOS, COX-2, MPO, IFN-γ, and NF-κB. ***Artemisia capillaris* Thunb.** from Asteraceae revealed remarkable early-stage anti-gastritis ability and anti-chronic gastritis and anti-precancerous lesion capacity by down-regulating IL-1β, IL-6, TNF-α, COX-2, PGE_2_, gastric F4/80 protein, and MDA with low p-p65 and pSTAT3 expression. The nine compounds ***Chaenomeles speciosa* total triterpenoids**, **Neutral corn protein hydrolysate**, **Artemisinin**, **Artesunate**, **Dihydroartemisinin** (all from ***Artemisia annua* L.**), **Capsaicin**, **Piperine**, **Caffeic Acid Phenethyl Ester** and ***Sophora alopecuroides* L. total alkaloids** down-regulated NF-κB signaling because of their low expression in p-IKK, p-IκBα, p65, and p50, which always accompany down-regulation of cytokines such as ILs-1β, −6, and −8, TNF-α, IFN-γ, the enzymes iNOS and COX-2, and NO, PGE_2_, MPO, and MCP-1. (Fig. 14 and Table 4, 5, 7).

**Table 7:** Signaling pathway of phytomedicine for HAG

Serial Number	Signaling Pathway	Plant extract OR Compound
1 (16 types)	NF-κB	*Persicaria capitata*, Korean Propolis, *Juglans regia* L*.*, *Angelica keiskei*, *Artemisia capillaris* Thunb*.*, *Camellia sinensis* L*.*, *Chaenomeles speciosa* total triterpenoids, Neutral corn protein hydrolysate, Artemisinin, Artesunate, Dihydroartemisinin, Curcumin, Capsaicin, Piperine, Caffeic Acid Phenethyl Ester, *Sophora alopecuroides* L. total alkaloids
2 (4 types)	JAK/STAT3	*Juglans regia *L., Licorice, *Artemisia capillaris* Thunb*.*, *Camellia sinensis* L
3 (3 types)	MAPK	*Alpinia officinarum* Hance, Quercetin, Palmatine
4 (2 types)	TLR4/MyD88	*Chaenomeles speciosa* total triterpenoids, Neutral corn protein hydrolysate
5 (2 types)	NLRP3/Caspase-1	*Persicaria capitata*, *Chaenomeles speciosa* total triterpenoids
6 (1 type)	PI3K/AKT	*Persicaria capitata*
7 (1 type)	NRF2/HO-1	*Juglans regia *L
1 (3 pathways)	NF-κB, PI3K/AKT, NLRP3/Caspase-1	*Persicaria capitata*
	NF-κB, JAK/STAT3, NRF2/HO-1	*Juglans regia *L
	NF-κB, TLR4/MyD88, NLRP3/Caspase-1	*Chaenomeles speciosa* total triterpenoids
2 (2 pathways)	NF-κB, JAK/STAT3	*Artemisia capillaris* Thunb., *Camellia sinensis* L
	NF-κB, TLR4/MyD88	Neutral corn protein hydrolysate

#### STAT3

The CagA is an apparent substance that activates STAT3 signaling [141]. Activated JAK phosphorylated the receptor’s cytoplasmic domain to create a docking site for Src homology 2 domain tyrosine phosphatase (SHP2)-containing signaling protein. The phosphorylation of a critical tyrosine residue (*Tyr705*) triggers STAT3 dimerization by contacting the phosphotyrosine-SH2 domain, thus mediating activation of STAT3, which binds to DNA sequences to stimulate target genes. JAK1/STAT3 is upstream of IL-8 and NF-κB in Helicobacter-infected gastric epithelial cells [104], and p-STAT3 level is related to poor survival in gastric adenocarcinoma patients [142]. Dysregulated STAT3 activation leads to VEGF overproduction and increased angiogenic phenotype in GC [143]. *c-Myc*, which is overexpressed after Helicobacter infection, is a STAT3 target gene and can compensate for the role of STAT3, contributing to gastric epithelial cell proliferation [144].

Helicobacter infection promoted ROS generation, which elevated IL-6 production and subsequent STAT3 phosphorylation in AGS cells [145]. STAT3 activation and subsequent tumor development do not occur without the Glycoprotein 130 (gp130) receptor, which is a signaling element of the IL-6R‒gp130 complex, and the IL-6 family member IL-11 is a promotion element of GC by activating STAT3 to overexpress proliferative genes in mice [146, 147]. The gp130F759/F759 mice extended gp130-induced STAT3 activation, whereas indicated a negative regulation for SHP2 [148]. Another study also found phosphorylated CagA boosted SHP2/ERK1/2 activity, whereas unphosphorylated CagA was inclined to activate STAT3 [149]. Therefore, stomach epithelial STAT3 targeting or IL-6R-gp130 blocking may be therapeutic ways to prevent gastric carcinogenesis [150].

Helicobacter-induced and high salt diet WT mice were sacrificed after 24 weeks (a time to establish a CAG model) and 36 weeks (a time to establish a GC model), respectively, after which NF-κB (p-p65) and STAT3 (pSTAT3) were activated and inflammatory biomarkers IL-6, COX-2, and PGE_2_ were overexpressed. ***Juglans regia*** **L.** treatment alleviated pathological damage to the gastric mucosa, including inflammation, ulcers, atrophy, and adenoma, and ameliorated NF-κB, p-p65, STAT3, pSTAT3, IL-6, COX-2/COX-1, PGE_2_, c-Myc, and Ki-67, whereas increasing the levels of the defensive protein 15-PGDH and the JAK/STAT regulator suppressor of cytokine signaling 1 (SOCS-1) [17]. Jeong M et al. [37] generated CAG and GC mouse models in the same way and fed them ***Artemisia capillaris* Thunb.** or ***Camellia sinensis* L.**. The two plant-derived extracts decreased p-p65, pSTAT3, IL-1β, IL-6, TNF-α, COX-2, PGE_2_, and gastric F4/80 protein but preserved the protective protein 15-PGDH. Park JM et al. [27] treated *Il-10(− / −)* mice with a high salt diet for 24 weeks and reported intense gastric inflammation and nodular lesions with granular gastric mucosa. **Licorice** attenuated inflammation and tumorigenesis by suppressing p-JAK2 and p-STAT3 production, reducing ILs-1β, −6, and −8, TNF-α, iNOS, COX-2, PGE_2_, and other cytokine arrays for inflammation and tumorigenesis, including bFGF and FcrRIIB. The anti-HAG and GC potencies of ***Juglans regia*** **L.**, **Licorice**, ***Artemisia capillaris* Thunb*.***, and ***Camellia sinensis* L** may share the same mechanism: the IL-6R/gp130/JAK/STAT3 pathway. (Fig. 14 and Table 4, 5, 7).

#### MAPK

MAPKK and MAPKKK are key kinases involved in MAPK signaling, which regulates inflammatory response, cell growth, differentiation, proliferation, and apoptosis [151]. MAPKs include three primary members: extracellular-signal-regulated kinases (ERK), c-jun N-terminal kinases (JNK), and MAPK14 (p38α). IL-8 and IL-17 are the dominant cytokines that induce inflammation and carcinoma. IL-17 can induce cytokines such as TNF-α, IL-1β, and IL-8 [152]. ERK1/2, p38 MAPK, and AP-1 mediate IL-8 secretion during Helicobacter infection [153–155]. For example, peptidoglycan injected via the T4SS is recognized by NOD1, thereby eliciting NF-κB, MAPK, and AP-1 activation, which initiate the production of cytokines. LPS from Helicobacter causes monocytic lineage cells to release IL-8 through NF-κB and MAPK pathways [155]. ERK1/2 and NF-κB signaling are two ways in which IL-17 can increase IL-8 expression [156, 157]. In addition, c-Fos and c-Jun proteins, which are essential for cell proliferation, combine to form AP-1, which produces IL-8 with the help of activating NF-κB. Likewise, CagA interacts with ERK1/2 [158] and p38 MAPK. CagA translocates into human B lymphoid cells, where it interacts with SHP2, which is indispensable in p38 MAPK pathway activation, causing the up-regulation of Bcl-2 and Bcl-x [159].

***Alpinia officinarum* Hance** has good efficiency in suppressing gastric inflammation response by reducing p-ERK1/2, p-JNK, and p-p38 and ameliorating downstream production of IL-1β, IL-17, and TNF-α. **Quercetin** from the plant ***Polygonum capitatum*** restrained the MAPK pathway by reducing p38 MAPK and IL-8 levels. **Quercetin** presented remarkable capacity on regulating the equilibrium between apoptosis and proliferation that declined G0/G1, G2/M phase cells, and Bax but enhanced S-phase cells and Bcl-2.

It has been confirmed that **Palmatine**, a botanical isoquinoline alkaloid, was initially isolated from ***Coptis chinensis* Franch.**, suppressed ADAM17/EGFR signaling to inhibit MMP10 generation, thereby elevating the anti-inflammatory effect. On the other hand, **Palmatine** increased Reg3a levels but decreased CXCL16 production, therefore ameliorating pathological damage and improving the host's defensive ability. EGFR is responsible for the increase in MMP10. EGFR interacts with EGFR ligands such as HB-EGF, whose extracellular domain should be shed with the aid of ADAM17. **Palmatine** hampered the cleavage of HB-EGF by ADAM17 in Helicobacter infection rats. MMP10, which has a significant correlation with CagA-positive Helicobacter infection, can control cytokine-associated chemotaxis, which causes leukocytes to migrate to the infected site and aids in the evolution of inflammation. This might be a way by which Helicobacter infection induces hyperplastic polyps and gastric cancer [160]. Inhibitors targeting ERK1/2 and JNK impeded such MMP10 secretion, which indicated MAPK may be upstream of MMP10 [161]. (ADAM17-HB-EGF/EGFR/MMP10, MAPK/MMP10) (Fig. 14 and Table 4, 5, 7).

#### TLR4/MyD88

TLR4, a key Toll-like receptor in the HAG process, induced inflammation on the stomach through the complex of MyD88, IL-1R-associated protein kinase (IRAK), and tumor necrosis factor receptor-associated factor 6 (TRAF6), resulting in the upregulation of NF-κB, ERK, JNK, and p38 and the activation of proinflammatory cytokines such as IL-6. IL-1 and LPS induced the TLR4/MyD88 signaling pathway activation [162]. Two compounds from included studies, ***Chaenomeles speciosa* total triterpenoids** and **Neutral corn protein hydrolysate**, dampened TLR4 and MyD88 expression, which restrained NF-κB signaling as well and relieved mucosal damage, including swelling and inflammatory infiltration. ***Chaenomeles speciosa*** total triterpenoids significantly relieved inflammation by declining levels of MPO, NLRP3, and multiple cytokines (IL-1β, IL-6, IL-18, KC, TNF-α, and MCP-1). **Neutral corn protein hydrolysate** attenuated gastritis through reduction of MPO, IL-1β, IL-6, KC, TNF-α, and MCP-1.

TLR4/MyD88/NF-κB could regulate the cell balance between apoptosis and proliferation as well. Silencing high-mobility group protein B1 (HMGB1)/TLR4/MyD88 signaling, which inhibited the downstream NF-κB pathway, prevented overproliferation. The same study revealed that inhibiting HMGB1 down-regulated Bcl-2 and MMP-2 but up-regulated Bax in gastric cancer cells [163]. ***Chaenomeles speciosa* total triterpenoids** increased the levels of the pro-proliferative factors Bcl-2 and Bcl-xl but decreased the levels of the pro-apoptotic factor Bax, which inhibits over-apoptosis, possibly via the NF-κB pathway. This compound derived from ***Chaenomeles speciosa* (Sweet) Nakai** declined the formation of apoptotic vesicle, which is composed of Apaf-1, cytochrome C, and pro-caspase-9. (TLR4/MyD88/NF-κB) (Fig. 14 and Tables 4, 5, 7).

#### Others

***Persicaria capitata***, a plant from Polygonaceae, regulated NF-κB, PI3K/AKT, and NLRP3/Caspase-1 simultaneously to reduce Helicobacter density and relieve inflammatory cell infiltration and disordered arrangement of glands in SD rats gastric. ***Persicaria capitata*** is an inhibitor of NF-κB that alleviated HAG by reducing production of pro-IL-1β, IL-1β, IL-18, NLRP3, and pro-caspase-1, but elevating AKT and p-AKT levels. The interaction of *CagA* with activated Hepatocyte Growth Factor Receptor (Met) via its *CRPIA* motif is vital for downstream PI3K/AKT signaling stimulation and pleiotropic transcriptional responses, such as those involving β-catenin and NF-κB [164]. Inhibiting AKT helps NF-κB dissociate from IκB, further regulating downstream target gene expression, such as triggering the release of inflammatory factors. GES-1 cells and Helicobacter infection in vivo aggravate the inflammatory response by down-regulating AKT and increasing NF-κB, which induce NLRP3, pro-IL-1β, IL-1β, and IL-18. Furthermore, NLRP3 further induces increased levels of IL-1β and IL-18 [12]. (PI3K/AKT/NF-κB).

The NLRP3 inflammasome is a notable factor in host response to microbes and tissue lesions, which elicits inflammatory and apoptotic action. Helicobacter virulence factors such as T4SS and FlaA stimulate NF-κB and AP-1 by pattern-recognition receptors (PRRs), and subsequently NLRP3 oligomerizes and capsase-1 activates, which will cleave pro-IL-1β [165]. ***Persicaria capitata*** decreased IL-1β, IL-18, pro-IL-1β, NLRP3, and pro-caspase-1. CAG mice infected with Helicobacter were given ***Chaenomeles speciosa*** **total triterpenoids**, and their chronic gastritis and atrophy glands were improved by the reduction of thioredoxin-interacting protein (TXNIP), NLRP3, pro-caspase-1, and caspase-1 levels. (NLRP3/Caspase-1).

Nuclear factor erythroid-2-related factor 2 (NRF2) plays a vital role in cell autophagy in order to conflict oxidative stress and inflammation in Helicobacter infection cells and mice [166]. The inducible host defensive enzyme Heme Oxygenase-1 (HO-1), whose generation requires NRF2, regulates antioxidative stress processes to suppress CagA action [167]. ***Juglans regia* L.** lowered Kelch-like ECH-associated protein 1 (Keap1) levels while increasing NRF2 and HO-1 expression to prevent inflammation and oxidative stress in gastric mucosa. (NRF2/HO-1) (Fig. 14 and Table 4, 5, 7).

### Traditional Chinese Medicine treating HAG

TCM has rich experience in treating gastrointestinal diseases. Nine plants, including Zhizi (Ripe ruit extract, Geniposide, Genipin), Gancao (plant extract, 18β-Glycyrrhetinic Acid), Dasuan (Clove extract), Huzhang (whole plant extract, Quercetin), Huangqin (Baicalin, Baicalein), Huanglian (Epiberberine, Coptisine, Palmatine), Qinghao (Artemisinin, Artesunate, Dihydroartemisinin), Niubangzi (Arctigenin), Jianghuang (Curcumin) from TCM are promising plant medicines because these botanical medicines extracts or compounds derived from them are most frequently administrated in rats or mice for HAG of the included literatures and are expected for more in-depth studies. As Table 6 shows, 25 plants for HAG are commonly used medicines in TCM. Among these TCM medicines, four plants have notable anti-bacterial activities (Shiliupi, Dasuan, Wumei, and Ganqi) and fifteen plants have significant anti-inflammatory effects (Zhizi, Huzhang, Gancao, Dasuan, Yinchen, Mugua, Huangqin, Yinyanghuo, Huangqi, Niubangzi, Huanglian, Qinghao, Hujiao, Zhishi, and Zhiqiao), which indicate TCM has good advantages and prospects in the discovery of anti-HAG drugs. To discuss further, we found seven plants are conducive for stomachache (Yanhusuo, Huzhang, Gancao, Gaoliangjiang, Mugua, Yinyanghuo, Jianghuang), nine for flatulence (Yanhusuo, Daoya, Mugua, Ganqi, Jianghuang, Lajiao, Hujiao, Zhishi, Zhiqiao), and seven for gastrointestinal function improvement (Gancao, Daoya, Gaoliangjiang, Huangqi, Lajiao, Hujiao, Fengjiao). In addition, nine plants are beneficial for hyperemia, erythema, and hemorrhage in mucous membrane, ulcer, and are labeled “huo xue hua yu, po xue tong jing, yang xue zhi xue” (Chinese pinyin) in TCM (Yanhusuo, Shiliupi, Zhizi, Huzhang, Dasuan, Huangqin, Huangqi, Ganqi, Jianghuang), which deserved to investigate their potency for precancerous conditions such as atrophy, intestinal metaplasia, and carcinoma. (Fig. 10 and Table 6).

Numerous TCM formulas are commonly used in clinics for their more effective and versatile efficacy than a single plant medicine. Banxia Xiexin decoction, which contains berberine, palmatine, baicalein, and glycyrrhizin, is more instructive to HAG, gastric atrophy, and IM than control group patients [168]. Multiple classical ancient formulas from TCM, including Banxia Xiexin decoction [169], Qingwei San, Huanglian Wendan decoction [170], and Zuojin pill [171], which all contain Huanglian (*Coptis chinensis* Franch*.*) have superior anti-inflammatory activities and higher Helicobacter eradication rates. Jianghuang (*Curcuma longa* L.) and Gaoliangjiang (*Alpinia officinarum* Hance) and Shengjiang/Ganjiang (*Zingiber officinale* Roscoe) are three noteworthy herbs from Zingiberaceae curing inflammation, pain, and digestion disease on stomach in TCM, and they are common medicine used in decoctions for HAG, such as Banxia Xiexin decoction, Huangqi Jianzhong decoction, and Gancao Ganjiang decoction. Furthermore, several plants, including *Panax ginseng* C. A. Mey (Renshen), Rhei Radix Et Rhizoma (Dahuang), *Poria cocos* (Schw.) Wolf (Fuling), *Panax notoginseng* (Burk.) F. H. Chen (Sanqi), and *Pineilia ternata* (Thunb.) Breit (Banxia), are highly potential herbal medicines as well [170], though there are not detailed studies for their anti-HAG potency in animal models. (Fig. 10 and Table 6).

### Clinical studies of phytomedicine used in HAG

Among the phytomedicines we included, several well-studied plant extracts or plant-derived compounds have progressed to clinical trials. For example, randomized, double-blind, controlled trials have demonstrated that broccoli can alleviate inflammatory syndrome in patients [172] and prevent lipid peroxidation in the mucosa [173]. However, when administered alone, it is ineffective in eradicating Helicobacter. A randomized, placebo-controlled study indicated that administration of broccoli sprouts, which contain sulforaphane, an isothiocyanate with potent anti-inflammatory and antioxidant properties, has been shown to reduce levels of urease, Helicobacter stool antigen, and serum pepsinogens I and II [174]. Besides, *Glycyrrhiza glabra* (licorice) has also entered clinical trials in HAG, and randomized controlled clinical trials demonstrated licorice alleviated Helicobacter infection, chronic inflammation, and gastrointestinal symptoms in humans [175, 176]. A randomized double-blind, placebo-controlled study revealed that β-caryophyllene relieved Helicobacter-infected patients’ nausea and epigastric pain and decreased the serum IL-1β levels [177]. Additionally, berberine [178], mastic gum [179], Japanese apricot (*Prunus mume* Siebold et Zucc.) [180], Korean red ginseng [181], and Brazilian green propolis [182] have demonstrated positive effects on Helicobacter eradication in clinical randomized controlled trials. Further investigation into optimal dosages and co-administration strategies in human clinical trials is warranted. Moreover, a South Korean clinical study observed that the intake of total dietary carotenoids or specific carotenoid subclasses was inversely correlated with the risk of GC. This association was also evident in patients infected with Helicobacter [183]. Furthermore, a population-based study in China suggested that garlic consumption was inversely associated with Helicobacter infection and may have a preventive effect on GC [184]. However, two large prospective cohort studies in the United States found no significant association between garlic intake and the risk of Helicobacter infection or gastric cancer [185].

Moreover, after conducting an exhaustive search of the official websites of WHO's Tier 1 Clinical Trial Registries (including those of China, the United States, the European Union, Japan, and Iran), we found that berberine, when combined with antibiotics and acid inhibitors, constitutes a significant proportion of therapeutic regimens in ongoing or completed clinical trials for HAG. These protocols are anticipated to provide additional clinical observational evidence in the future. Their registration numbers are as follows: ChiCTR2300077074, ChiCTR-IOR-17013319 (for more information, visit http://www.chictr.org.cn), NCT06603688, NCT06514274, NCT05014334, NCT04697186, NCT03609892, NCT02633930, and NCT02296021 (for more information, visit http://clinicalTrials.gov/). In addition, the registration protocols for the clinical trials of Banxia Xiexin Decoction (ChiCTR2000034509, for more information, visit http://www.chictr.org.cn. NCT06340724, for more information, visit http://clinicalTrials.gov/) in the treatment of HAG merit particular attention. This is because Banxia Xiexin Decoction is extensively utilized in clinical practice for HAG treatment in China [186], and berberine, an important component derived from Huanglian (*Coptis chinensis* Franch.), plays a crucial role in this formula. Therefore, we conclude that the aforementioned plant extracts and compounds hold significant potential for clinical development.

## Conclusion and future prospect

This systematic review demonstrates that plant-derived extracts and compounds have favorable anti-Helicobacter and anti-inflammatory properties through modulating different mechanisms and signaling pathways including NF-κB, JAK2/STAT3, MAPK, TLR4/MyD88, PI3K/AKT, NLRP3/Caspase-1 and NRF2/HO-1. Further exploration of the application of plant extracts and compounds to humans is needed.

For HAG, bacterial infection and inflammation are the earliest lesions. This systematic review concentrates on the field of phytopharmaceuticals through a comprehensive search of databases, focusing on anti-Helicobacter and anti-inflammatory effects as essential indications to identify therapies that can curb HAG at an early stage. Both traditional alternative therapies and modern medicine agree on the importance of early treatment in reversing disease outcomes and improving patient prognosis [187]. According to the Correa cascade [3], infection and gastritis are the starting points for later atrophy, hyperplasia and cancer, so the active search for antimicrobial and anti-inflammatory [2] phytomedicines is highly valuable for the prevention of precancerous lesions and cancer. As presented in Fig. 9. (Phytomedicines act on Correa cascade), we identify plants or compounds based on their advantages at different stages, which will facilitate researchers in selecting a specific plant or compound corresponding with their study purpose.

Additionally, the ever-increasing antibiotic resistance has led to low efficiency in eradicating Helicobacter. Urgent requirements for novel drugs or new personalized combined therapies are challenging assignments for researchers. Aside from the inefficient bacteria elimination dilemma, the overuse of antibiotics elicits gut microbiota alterations, which could induce multiple gastrointestinal invalidities such as low digestive and absorptive function and inflammatory bowel disease [188]. This systematic review, which excavates therapeutic medicinal plants and bioactive phytochemicals, may alleviate the pressing need for antibiotic replacement and gastrointestinal microbiota-regulating drugs.

According to our comprehensive findings, sixteen families, especially Asteraceae, Fabaceae and Rosaceae , and compounds from Terpenoids, Alkaloids, Phenols, and Flavonoids that are potential candidates for new drugs treating HAG show promise for clinical trials. Terpenes, which could transform into Terpenoids and act as anti-oxidative active substances in bodies, are worth studying. Owing to the close relationship between Flavonoids’ structure and activity, these natural agents possess an outstanding anti-Helicobacter effect [189]. *Prunus mume* Sieb. et Zucc. (Wumei) and *Chaenomeles speciosa* (Sweet) Nakai (Mugua) from Rosaceae; *Curcuma longa* L. (Jianghuang) and *Alpinia officinarum* Hance (Gaoliangjiang) from Zingiberaceae; *Artemisia annua* L. (Qinghao), *Arctium lappa* L. (Niubangzi), and *Artemisia capillaris* Thunb*.* (Yinchen) from Asteraceae are promising sources for new drugs. We propose researchers prioritize plants or compounds belonging to these families or compound classes, as they may offer promising prospects for enhanced clinical outcomes and novel drug development within this domain. (Figs. 11 and 12).

Furthermore, we have summarized plant extracts and plant-derived compounds that hold significant potential for clinical development, thereby providing readers with valuable insights. Broccoli, licorice, *Prunus mume* Siebold et Zucc., mastic gum, Korean red ginseng, Brazilian green propolis, garlic, β-caryophyllene, berberine, and carotenoids have been evaluated in clinical trials, demonstrating their potential value in the treatment of HAG. Notably, berberine shows promise as a potential combination drug for triple or quadruple therapies, supported by a series of high-quality completed or ongoing clinical trials. The mechanisms of multiple components in Banxia Xiexin Decoction warrant further investigation, given its prominence as a traditional medicine for gastritis and the fact that berberine is derived from this decoction.

Nevertheless, quality of included studies in this systematic review was medium. For instance, some studies had incomplete randomization method and blinding information regarding the experimental procedure, which made it difficult to ensure the accuracy of evaluation using the SYRCLE tool [9]. Additionally, all studies were conducted on animal models due to their physiological similarities with humans; thus, it remains uncertain whether these plant-derived components can be effective in humans. Furthermore, diverse methods employed in animal models and drug administrations pose challenges when comparing therapies efficacy. Intervention of extracts or compounds is various; indicators and parameter units of anti-Helicobacter or anti-inflammatory activities are multiple, which lead to difficulty in data incorporation and cause a comprehensive meta-analysis or one specific intervention meta-analysis hard to achieve.

## Supplementary Information

Supplementary Material 1

## Data Availability

Not applicable.

## Abbreviations

HAG: Helicobacter-associated gastritis
Hp: Helicobacter
CagA: Cytotoxin-associated gene A protein
VacA: Vacuolating cytotoxin A
IL-1β: Interleukin-1β
TNF-α: Tumor necrosis factor α
IFN-γ: Interferon γ
iNOS: Inducible nitric oxide synthase
NO: Nitric oxide
COX-2: Cyclooxygenase-2
PGE_2_: Prostaglandin E_2_
ROS: Reactive oxygen species
LPS: Lipopolysaccharides
IKK: IκB kinase
IκBα: Inhibitor of kappa B
JAK: Janus kinase
STAT3: Signal transducer and activator of transcription 3
MEK: Mitogen-activated extracellular signal-regulated kinase
ERK1/2: Extracellular signal-regulated kinase 1/2
JNK: Jun N-terminal kinase
PI3K: Phosphoinositide 3-kinase
AKT: Protein kinase B
TLR4: Toll-like receptor 4
MyD88: Myeloid differentiation primary response gene 88
NLRP3: NOD-like receptor thermal protein domain associated protein 3
AP-1: Activator protein 1
Keap1: Kelch-like ECH-associated protein1
NRF2: Nuclear factor erythroid-2-related factor 2
HO-1: Heme oxygenase-1
