# Correction: Plant-derived extracts or compounds for Helicobacter-associated gastritis: a systematic review of their anti-Helicobacter activity and anti-inflammatory effect in animal experiments

**DOI:** 10.1186/s13020-025-01139-5

**Published:** 2025-08-08

**Authors:** Danni Chen, Wenlai Wang, Xiangyun Chen, Ning Liang, Jiawang Li, Wei Ding, Hongrui Zhang, Zhen Yang, Hongxia Zhao, Zhenhong Liu

**Affiliations:** 1 Dongzhimen Hospital, Beijing University of Chinese Medicine, No. 5 Haiyuncang, Dongcheng District, Beijing, 100700 China; 2 College of Traditional Chinese Medicine, Beijing University of Chinese Medicine, No. 11 Bei San Huan Dong Lu, Chaoyang District, Beijing, 100029 China; 3 Institute for Brain Disorders, Beijing University of Chinese Medicine, Beijing, 100700 China; 4 Institute of Basic Theory for Chinese Medicine, China Academy of Chinese Medical Sciences, No. 16 Nanxiaojie, Dongzhimen Nei, Dongcheng District, Beijing, 100700 China; 5 Institute of Basic Research in Clinical Medicine, China Academy of Chinese Medical Sciences, Beijing, 100700 China

**Correction: Chinese Medicine (2025) 20:53** 10.1186/s13020-025-01093-2

Following publication of the original article [1], the authors reported that Figs. 2, 9, 10, 11 and 12 needed to be updated.

The correct figures have been provided in this Correction.

The incorrect Fig. 2 is:

**Figure:**
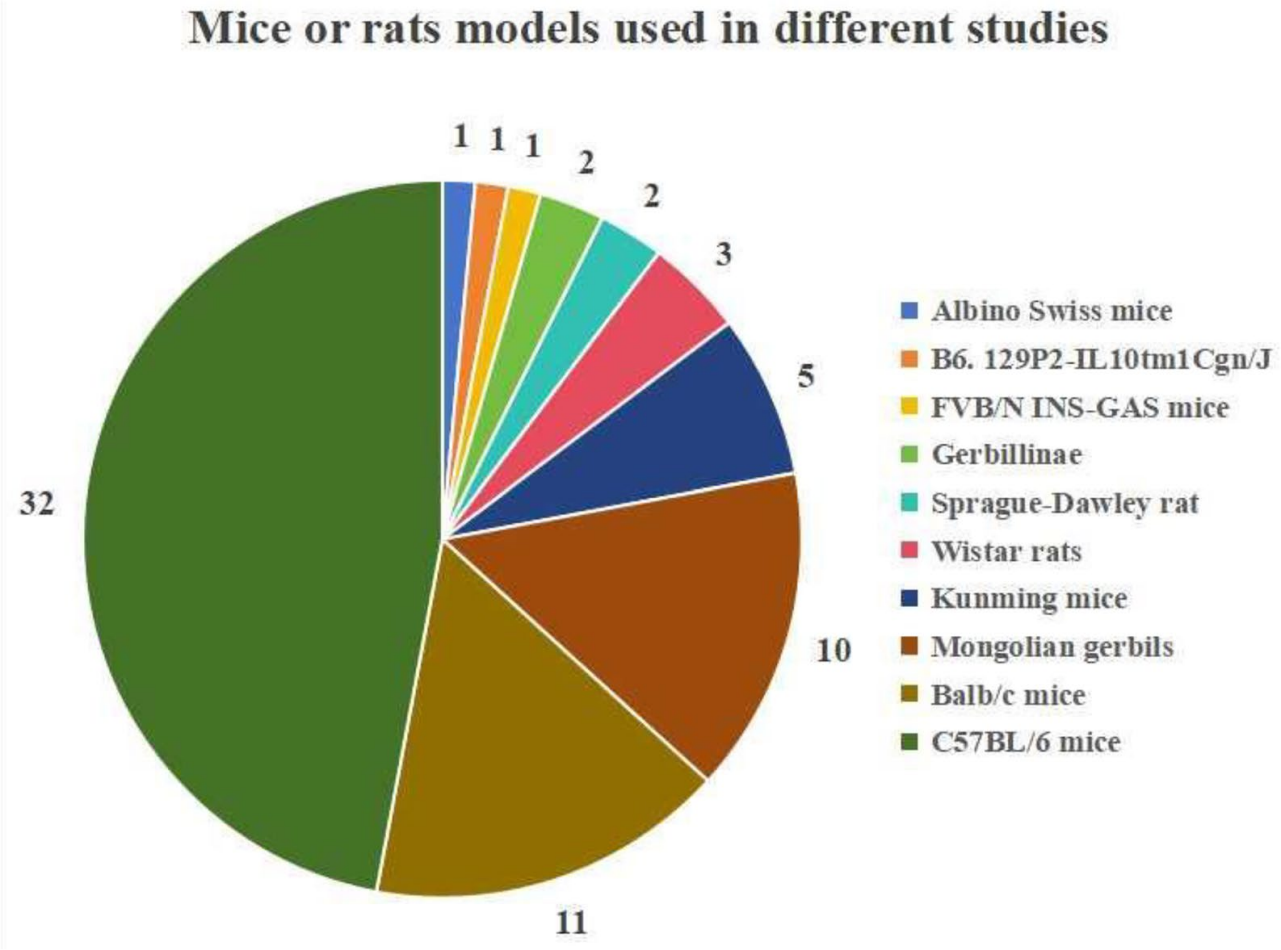
**Fig. 2.** Mouse or rat models used in different studies

The correct Fig. 2 is:

**Fig. 2:**
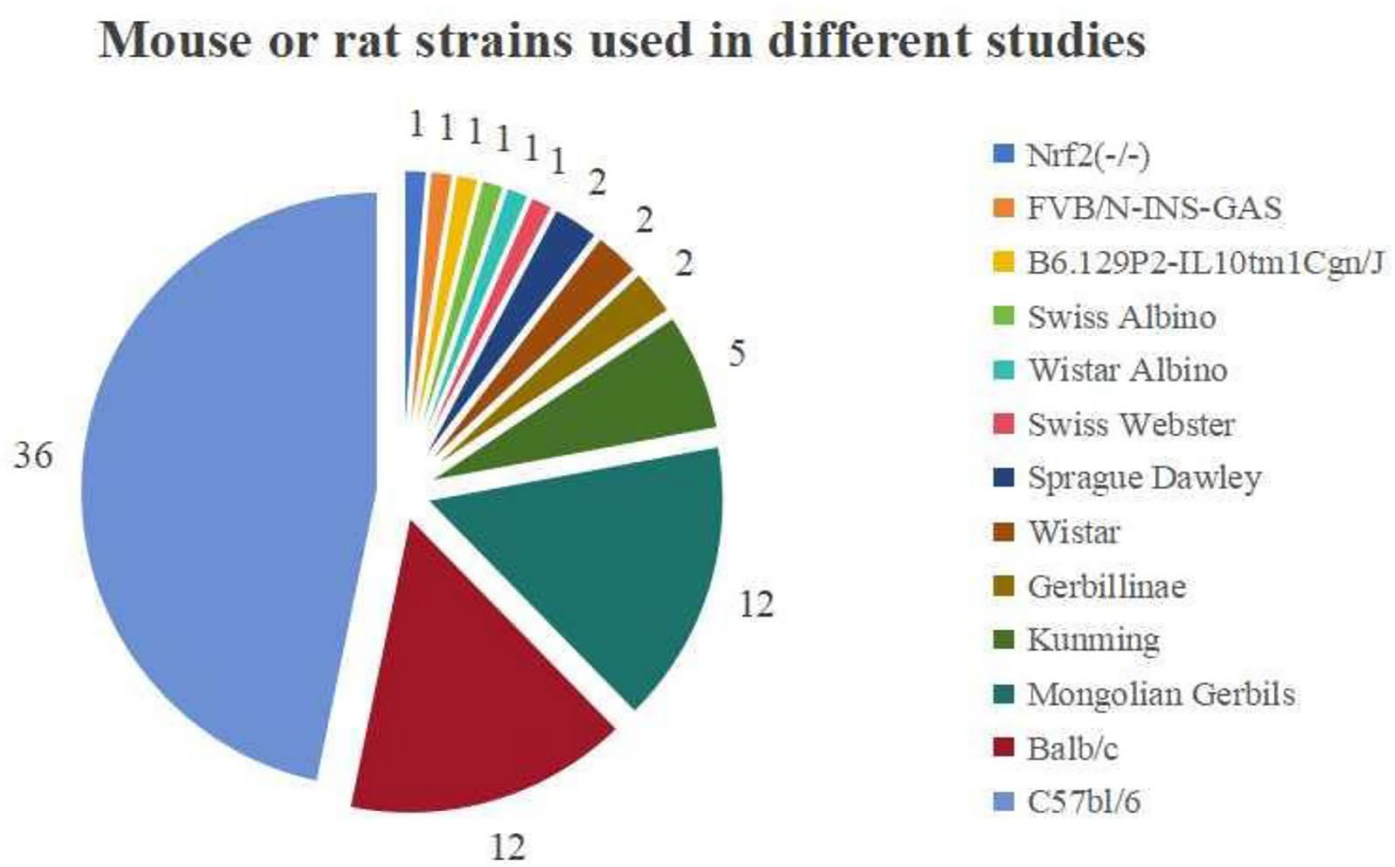
Mouse or rat strains used in different studies

The incorrect Fig. 9 is:

**Figure:**
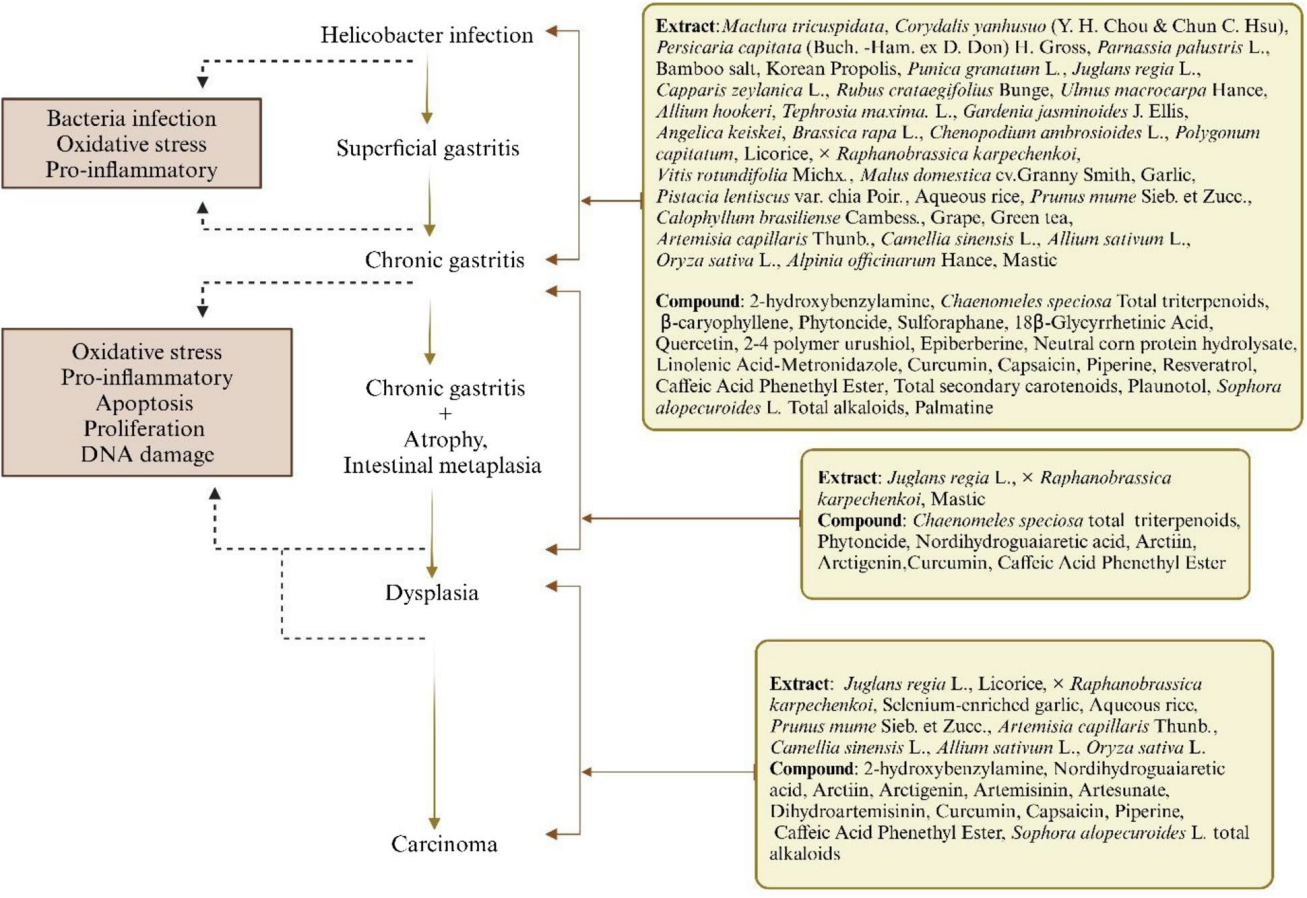
**Fig. 9.** Phytomedicines act on Correa cascade. (Created in BioRender. https://BioRender.com/daegq0t. Agreement number: YF2826GU41.)

The correct Fig. 9 is:

**Fig. 9:**
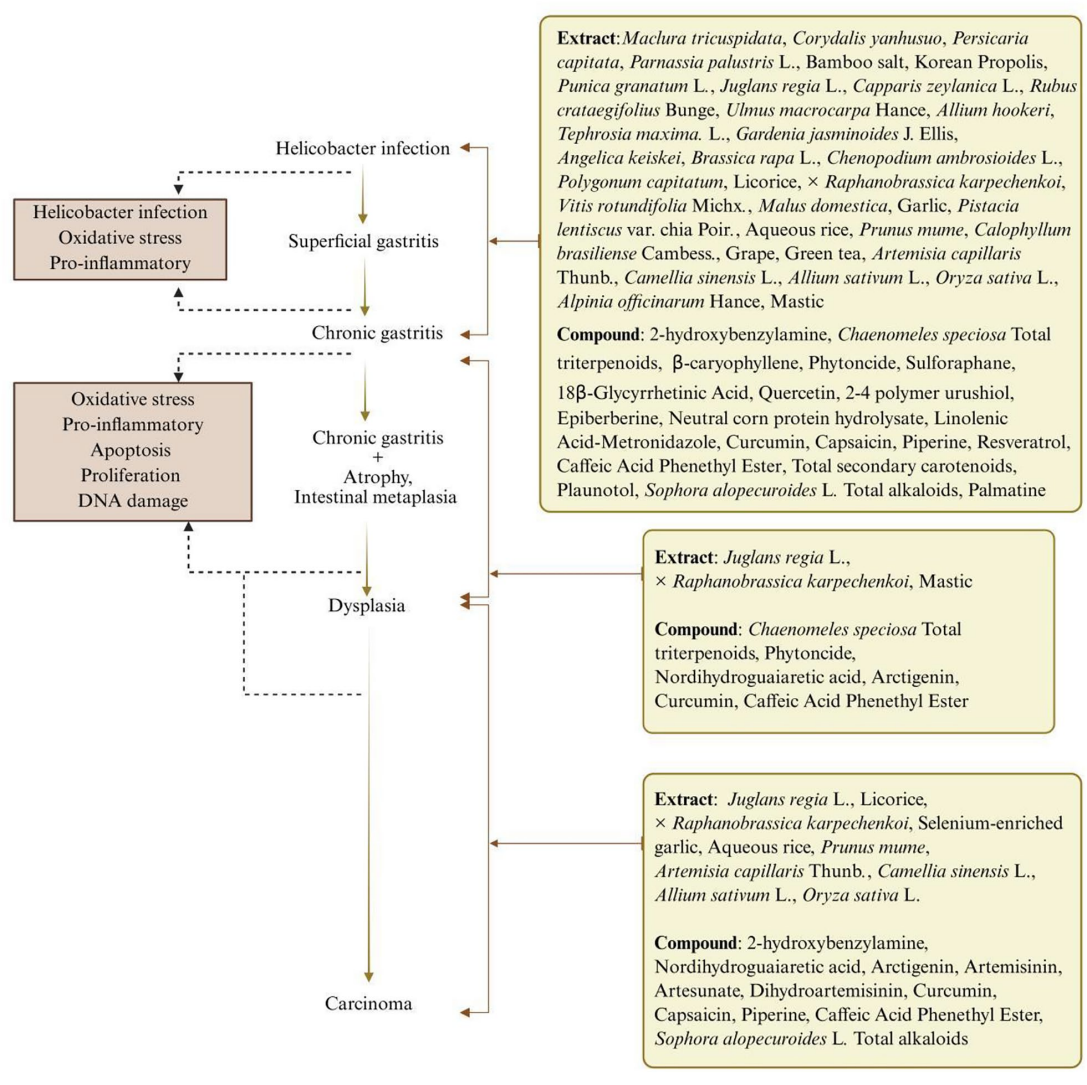
Phytomedicines act on Correa cascade. (Created in BioRender. https://BioRender.com/daegq0t. Agreement number: OK28AFSX59)

The incorrect Fig. 10 is:

**Figure:**
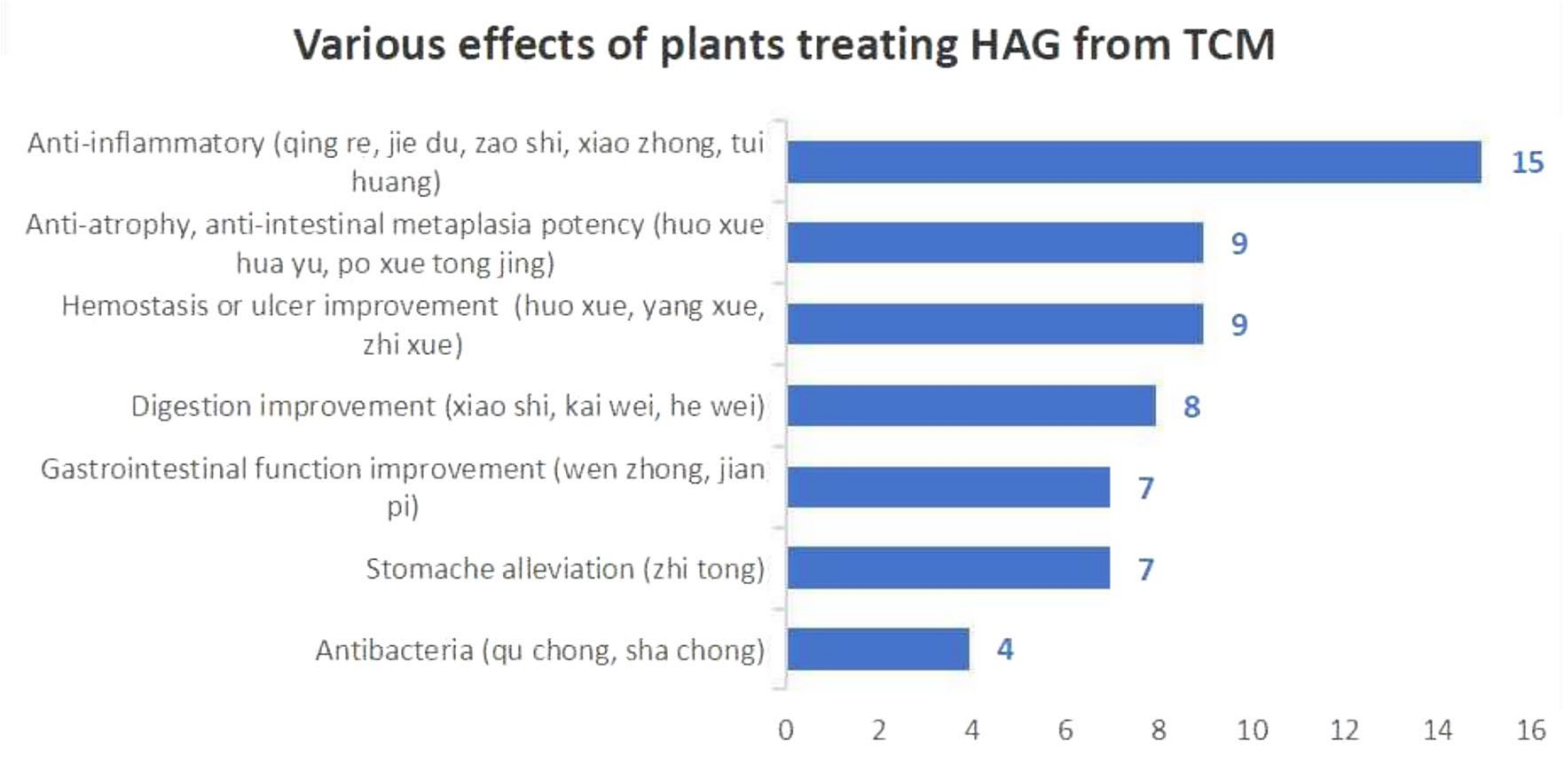
**Fig. 10.** Various effects of traditional Chinese medicine in treating HAG

The correct Fig. 10 is:

**Fig. 10:**
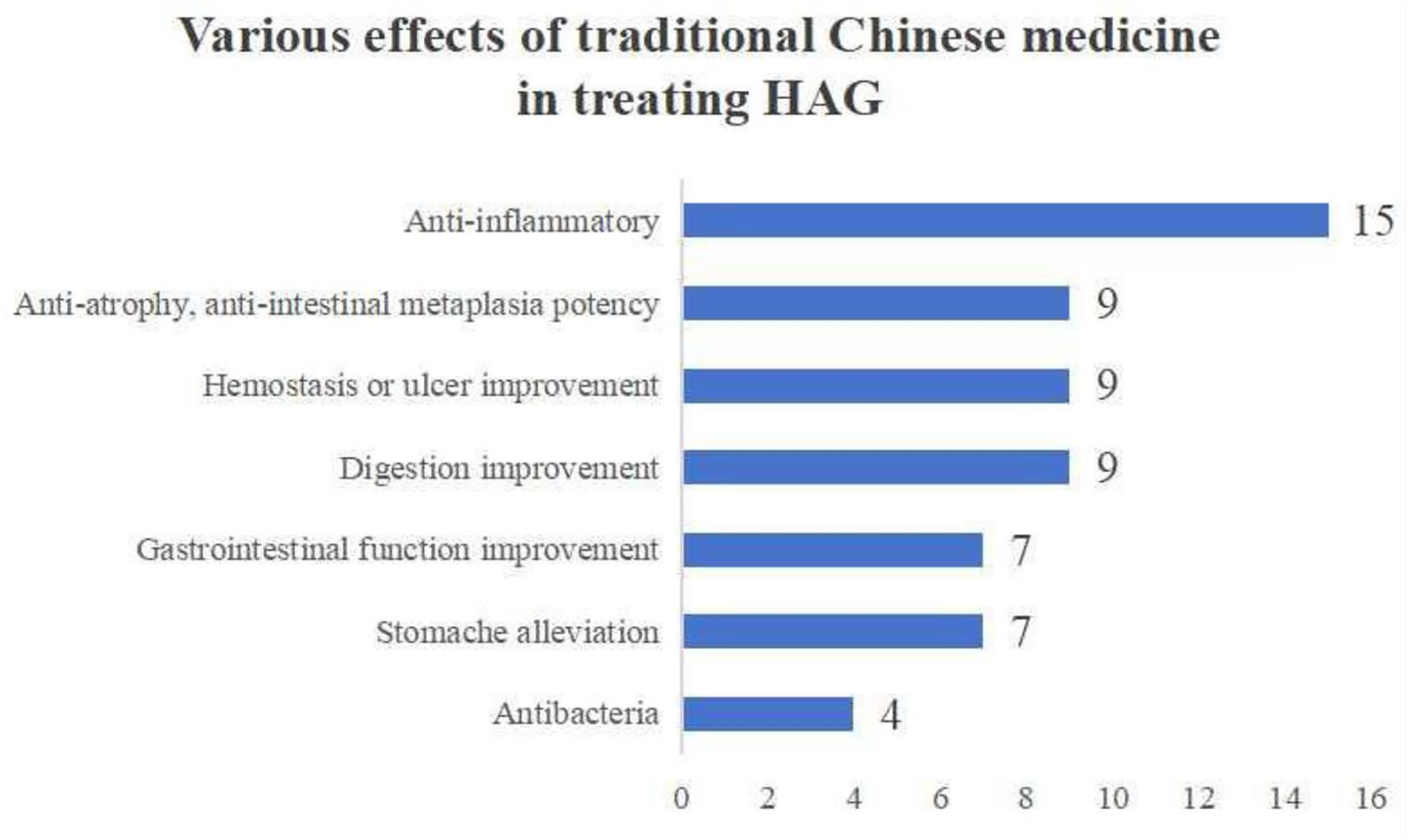
Various effects of traditional Chinese medicine in treating HAG

The incorrect Fig. 11 is:

**Figure:**
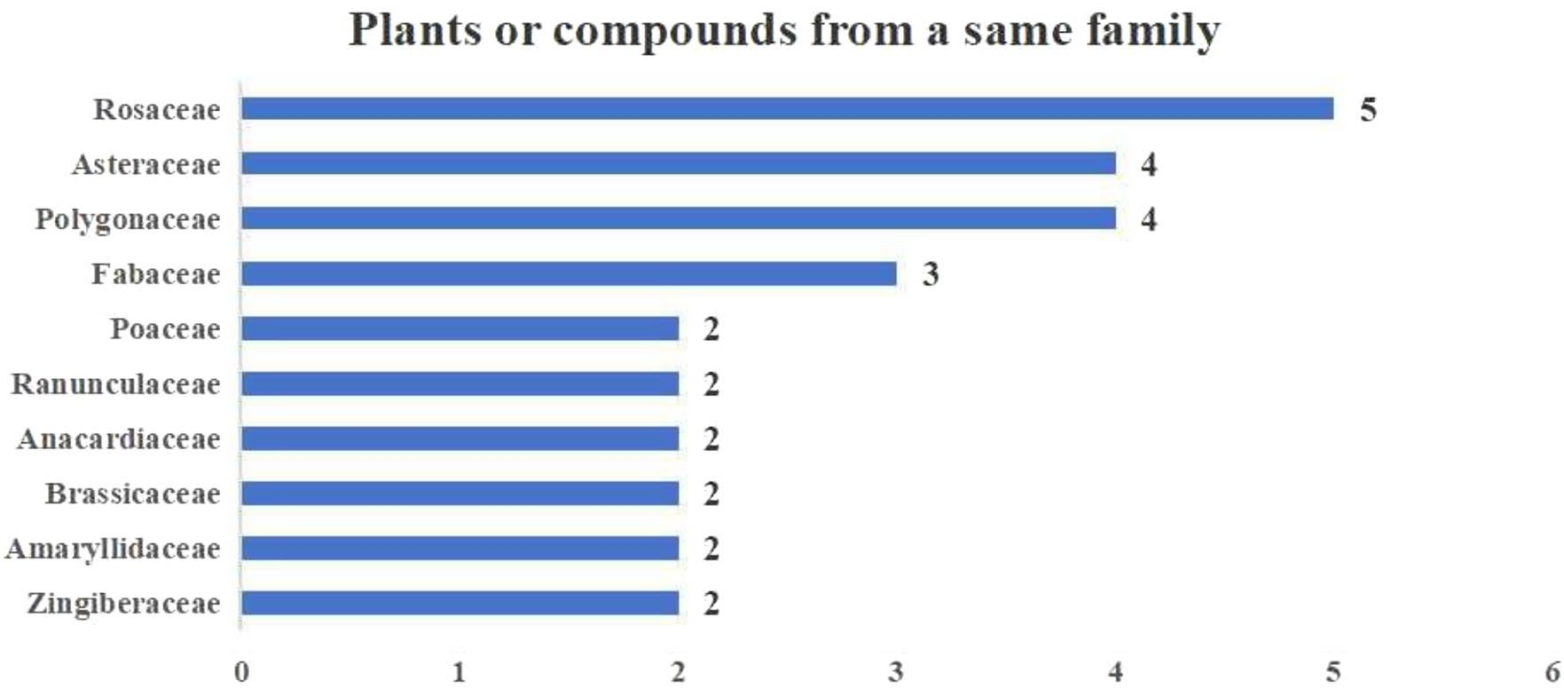
**Fig. 11.** Plants or compounds from a same family

The correct Fig. 11 is:

**Fig. 11:**
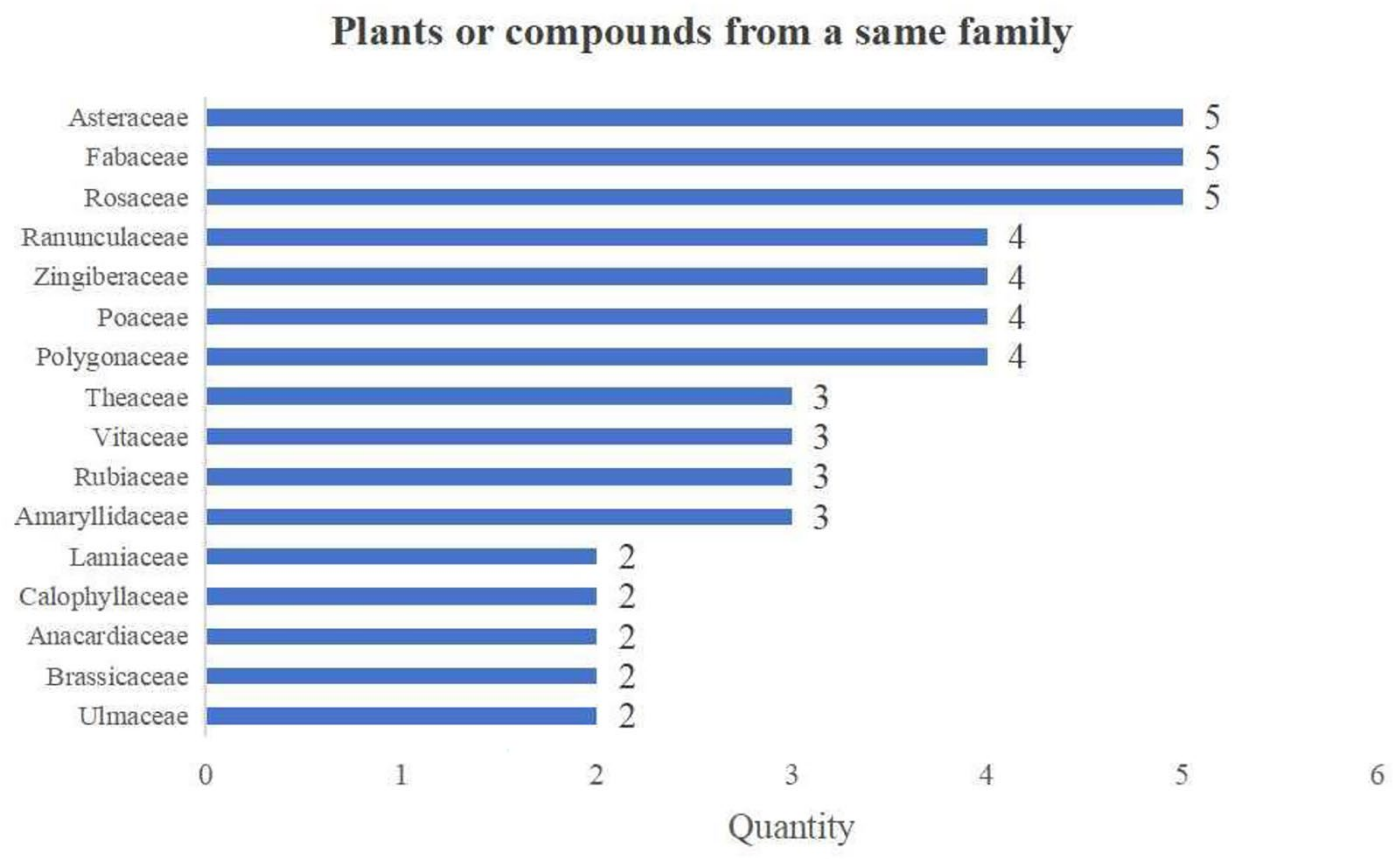
Plants or compounds from a same family

The incorrect Fig. 12 is:

**Figure:**
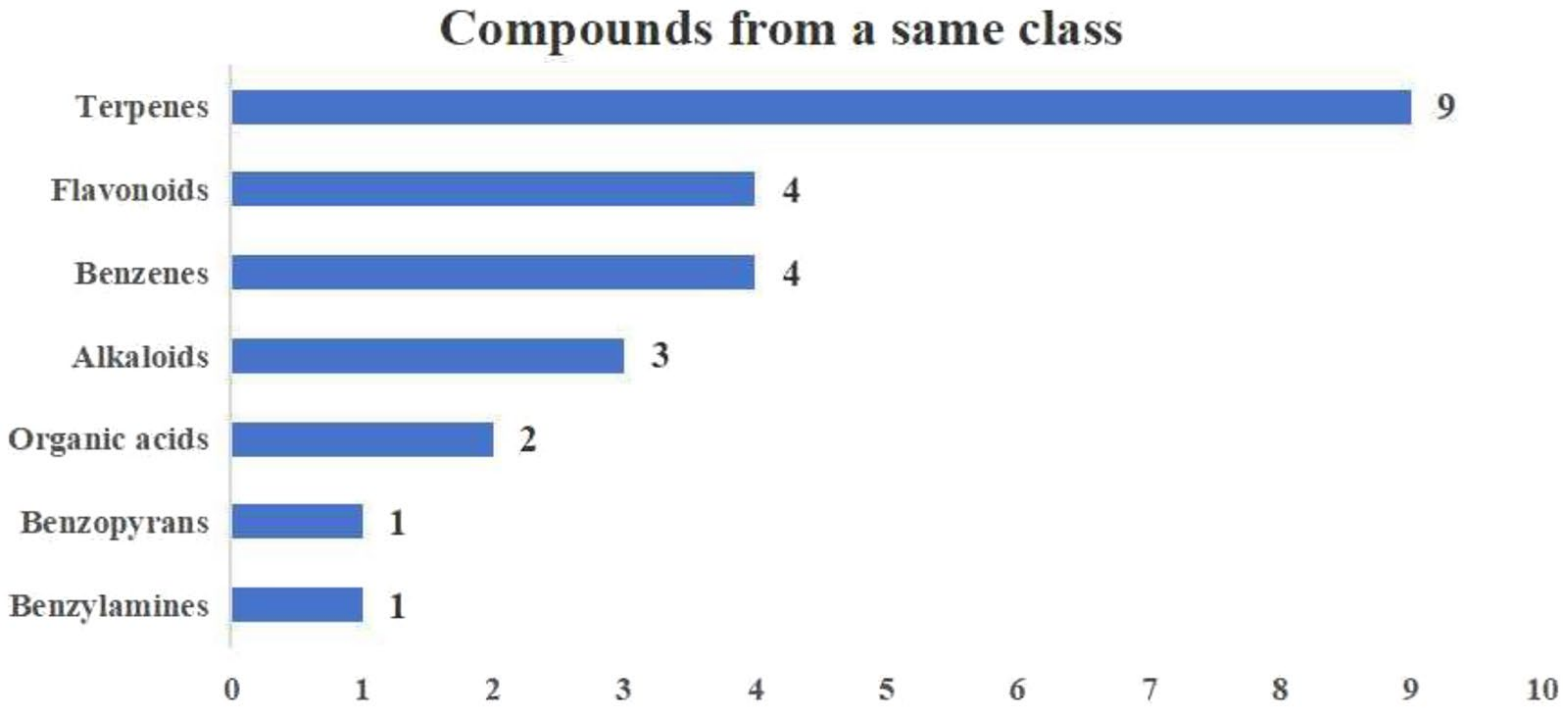
**Fig. 12** Compounds from a same class

The correct Fig. 12 is:

**Fig. 12:**
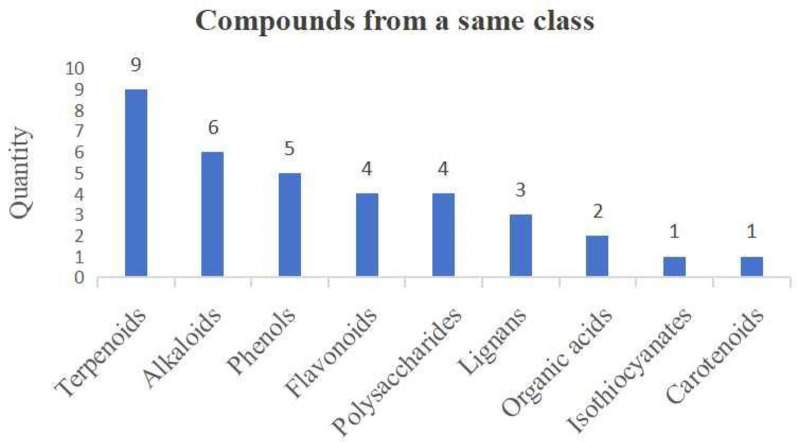
Compounds from a same class

The original article [1] has been corrected.
