# Supplementary material for: Plant-derived extracts or compounds for Helicobacter-associated gastritis: a systematic review of their anti-Helicobacter activity and anti-inflammatory effect in animal experiments
Source: Chin Med. 2025 Apr 22;20:53. doi: 10.1186/s13020-025-01093-2 (PMC12013188; doi:10.1186/s13020-025-01093-2)
Supplement: Supplementary file 1 — Supplementary Material 1 [file 13020_2025_1093_MOESM1_ESM.docx]

**Strategy of search**

## PudMed-372

P：

**"Helicobacter Infections"[Mesh]** OR “Infections,Helicobacter” OR “Helicobacter Infection” OR “Infection, Helicobacter”

I：

**"Plants"[Mesh] OR** “Plants” OR “Plant” OR **"Plants, Medicinal"[Mesh]** OR “Medicinal Plant” OR “Plant, Medicinal” OR “Medicinal Plants” OR “Medicinal Herbs” OR “Herb, Medicinal” OR “Medicinal Herb” OR “Herbs, Medicinal” OR “Pharmaceutical Plants” OR “Pharmaceutical Plant” OR “Plant, Pharmaceutical” OR “Plants, Pharmaceutical” OR “Healing Plants” OR “Healing Plant” OR “Plant, Healing” OR “Plants, Healing”

("Helicobacter Infections"[Mesh] OR "Infections, Helicobacter" OR "Helicobacter Infection" OR "Infection, Helicobacter") AND ("Plants"[Mesh] OR "Plants" OR "Plant" OR "Plants, Medicinal"[Mesh] OR "Medicinal Plant" OR "Plant, Medicinal" OR "Medicinal Plants" OR "Medicinal Herbs" OR "Herb, Medicinal" OR "Medicinal Herb" OR "Herbs, Medicinal" OR "Pharmaceutical Plants" OR "Pharmaceutical Plant" OR "Plant, Pharmaceutical" OR "Plants, Pharmaceutical" OR "Healing Plants" OR "Healing Plant" OR "Plant, Healing" OR "Plants, Healing")

## Scopus-526

P：

TITLE-ABS-KEY("**Helicobacter Infections**" OR “Infections,Helicobacter” OR “Helicobacter Infection” OR “Infection, Helicobacter”)

I：

TITLE-ABS-KEY(“Plants” OR “Plant” OR "Plants, Medicinal" OR “Medicinal Plant” OR “Plant, Medicinal” OR “Medicinal Plants” OR “Medicinal Herbs” OR “Herb, Medicinal” OR “Medicinal Herb” OR “Herbs, Medicinal” OR “Pharmaceutical Plants” OR “Pharmaceutical Plant” OR “Plant, Pharmaceutical” OR “Plants, Pharmaceutical” OR “Healing Plants” OR “Healing Plant” OR “Plant, Healing” OR “Plants, Healing”)

( TITLE-ABS-KEY ( "Helicobacter Infections" OR "Infections,Helicobacter" OR "Helicobacter Infection" OR "Infection, Helicobacter" ) ) AND ( TITLE-ABS-KEY ( "Plants" OR "Plant" OR "Plants, Medicinal" OR "Medicinal Plant" OR "Plant, Medicinal" OR "Medicinal Plants" OR "Medicinal Herbs" OR "Herb, Medicinal" OR "Medicinal Herb" OR "Herbs, Medicinal" OR "Pharmaceutical Plants" OR "Pharmaceutical Plant" OR "Plant, Pharmaceutical" OR "Plants, Pharmaceutical" OR "Healing Plants" OR "Healing Plant" OR "Plant, Healing" OR "Plants, Healing" ) )

## Web of science-621

P：

TS=("**Helicobacter Infections**" OR “Infections,Helicobacter” OR “Helicobacter Infection” OR “Infection, Helicobacter”)

I：

TS=(“Plants” OR “Plant” OR "Plants, Medicinal" OR “Medicinal Plant” OR “Plant, Medicinal” OR “Medicinal Plants” OR “Medicinal Herbs” OR “Herb, Medicinal” OR “Medicinal Herb” OR “Herbs, Medicinal” OR “Pharmaceutical Plants” OR “Pharmaceutical Plant” OR “Plant, Pharmaceutical” OR “Plants, Pharmaceutical” OR “Healing Plants” OR “Healing Plant” OR “Plant, Healing” OR “Plants, Healing”)

(TS=("Helicobacter Infections" OR “Infections,Helicobacter” OR “Helicobacter Infection” OR “Infection, Helicobacter”)) AND (TS=(“Plants” OR “Plant” OR "Plants, Medicinal" OR “Medicinal Plant” OR “Plant, Medicinal” OR “Medicinal Plants” OR “Medicinal Herbs” OR “Herb, Medicinal” OR “Medicinal Herb” OR “Herbs, Medicinal” OR “Pharmaceutical Plants” OR “Pharmaceutical Plant” OR “Plant, Pharmaceutical” OR “Plants, Pharmaceutical” OR “Healing Plants” OR “Healing Plant” OR “Plant, Healing” OR “Plants, Healing”))

## Embase-**975**

Embase——Session Results

| No. | Query Results | Results Date |
| --- | --- | --- |
| #13. | #5 AND #12 | 975 4 Nov 2023 |
| #12. | #6 OR #7 OR #8 OR #9 OR #10 OR #11 | 1,762,916 4 Nov 2023 |
| #11. | 'plants, medicinal' OR 'plant, medicinal' OR 'medicinal plants' OR 'medicinal herbs' OR 'herb, medicinal' OR 'medicinal herb' OR'herbs,medicinal'OR'pharmaceutical plants'OR'pharmaceuticalplant'OR'plant,pharmaceutical'OR 'plants, pharmaceutical' OR 'healing plants'OR 'healing plant' OR 'plant, healing' OR 'plants, healing' | 75,007 4 Nov 2023 |
| #10. | 'medicinal plant'/exp OR 'medicinal plant' | 331,751 4 Nov 2023 |
| #9. | 'medicinal plant'/exp | 317,919 4 Nov 2023 |
| #8. | 'plants' | 409,018 4 Nov 2023 |
| #7. | 'plant'/exp OR 'plant' | 1,695,611 4 Nov 2023 |
| #6. | 'plant'/exp | 1,253,003 4 Nov 2023 |
| #5. | #1 OR #2 OR #3 OR #4 | 50,360 4 Nov 2023 |
| #4. | 'helicobacter infections' OR 'infections, helicobacter' OR 'infection, helicobacter' | 390 4 Nov 2023 |
| #3. | 'gastritis due to h. pylori' OR 'gastritis due to helicobacter pylori' OR 'h. pylori gastritis' OR 'h. pylori infection' OR 'helicobacter gastritis' OR 'helicobacter infections' OR 'helicobacter pylori gastritis' OR 'helicobacter pylori infection' OR 'helicobacteriosis' OR 'infection by h. pylori' OR 'infection by helicobacter pylori' OR 'infection due to h. pylori' OR 'infection due to helicobacter pylori' OR 'infection of h. pylori' OR 'infection of helicobacter pylori' OR 'helicobacterinfection' | 50,331 4 Nov 2023 |
| #2. | 'helicobacter infection'/exp OR 'helicobacter infection' | 38,272 4 Nov 2023 |
| #1. | 'helicobacter infection'/exp | 38,078 4 Nov 2023 |

## CNKI-351

(主题=螺杆菌感染 + 幽门螺杆菌感染) AND (主题=植物 + 药用植物 + 中药 + 中草药)

## VIP-96

((题名或关键词=螺杆菌感染 OR 题名或关键词=幽门螺杆菌感染) AND (((题名或关键词=植物 OR 题名或关键词=药用植物) OR 题名或关键词=中药) OR 题名或关键词=中草药))

## Wanfang-152

题名或关键词:(螺杆菌感染 or 幽门螺杆菌感染) AND 题名或关键词:(植物 or 药用植物 or 中药 or 中草药)

## SinoMed/CBM-332

| 1 | "螺杆菌感染"[不加权:扩展] | [18870](javascript:historyLink('"%E8%9E%BA%E6%9D%86%E8%8F%8C%E6%84%9F%E6%9F%93"[%E4%B8%8D%E5%8A%A0%E6%9D%83:%E6%89%A9%E5%B1%95]')) |
| --- | --- | --- |
| 2 | "螺杆菌感染"[标题] OR "螺杆菌感染"[摘要] OR "幽门螺杆菌感染"[标题] OR "幽门螺杆菌感染"[摘要] | [12643](javascript:historyLink('"%E8%9E%BA%E6%9D%86%E8%8F%8C%E6%84%9F%E6%9F%93"[%E6%A0%87%E9%A2%98] OR "%E8%9E%BA%E6%9D%86%E8%8F%8C%E6%84%9F%E6%9F%93"[%E6%91%98%E8%A6%81] OR "%E5%B9%BD%E9%97%A8%E8%9E%BA%E6%9D%86%E8%8F%8C%E6%84%9F%E6%9F%93"[%E6%A0%87%E9%A2%98] OR "%E5%B9%BD%E9%97%A8%E8%9E%BA%E6%9D%86%E8%8F%8C%E6%84%9F%E6%9F%93"[%E6%91%98%E8%A6%81]')) |
| 3 | ("螺杆菌感染"[标题] OR "螺杆菌感染"[摘要] OR "幽门螺杆菌感染"[标题] OR "幽门螺杆菌感染"[摘要]) OR ("螺杆菌感染"[不加权:扩展]) | [22144](javascript:historyLink('("%E8%9E%BA%E6%9D%86%E8%8F%8C%E6%84%9F%E6%9F%93"[%E6%A0%87%E9%A2%98] OR "%E8%9E%BA%E6%9D%86%E8%8F%8C%E6%84%9F%E6%9F%93"[%E6%91%98%E8%A6%81] OR "%E5%B9%BD%E9%97%A8%E8%9E%BA%E6%9D%86%E8%8F%8C%E6%84%9F%E6%9F%93"[%E6%A0%87%E9%A2%98] OR "%E5%B9%BD%E9%97%A8%E8%9E%BA%E6%9D%86%E8%8F%8C%E6%84%9F%E6%9F%93"[%E6%91%98%E8%A6%81]) OR ("%E8%9E%BA%E6%9D%86%E8%8F%8C%E6%84%9F%E6%9F%93"[%E4%B8%8D%E5%8A%A0%E6%9D%83:%E6%89%A9%E5%B1%95])')) |
| 4 | ((("植物"[不加权:扩展]) OR "药用植物"[不加权:扩展]) OR "中药"[不加权:扩展]) OR "中草药"[不加权:扩展] | [506474](javascript:historyLink('((("%E6%A4%8D%E7%89%A9"[%E4%B8%8D%E5%8A%A0%E6%9D%83:%E6%89%A9%E5%B1%95]) OR "%E8%8D%AF%E7%94%A8%E6%A4%8D%E7%89%A9"[%E4%B8%8D%E5%8A%A0%E6%9D%83:%E6%89%A9%E5%B1%95]) OR "%E4%B8%AD%E8%8D%AF"[%E4%B8%8D%E5%8A%A0%E6%9D%83:%E6%89%A9%E5%B1%95]) OR "%E4%B8%AD%E8%8D%89%E8%8D%AF"[%E4%B8%8D%E5%8A%A0%E6%9D%83:%E6%89%A9%E5%B1%95]')) |
| 5 | (((("植物"[不加权:扩展]) OR "药用植物"[不加权:扩展]) OR "中药"[不加权:扩展]) OR "中草药"[不加权:扩展]) AND (("螺杆菌感染"[标题] OR "螺杆菌感染"[摘要] OR "幽门螺杆菌感染"[标题] OR "幽门螺杆菌感染"[摘要]) OR ("螺杆菌感染"[不加权:扩展])) | [332](javascript:historyLink('(((("%E6%A4%8D%E7%89%A9"[%E4%B8%8D%E5%8A%A0%E6%9D%83:%E6%89%A9%E5%B1%95]) OR "%E8%8D%AF%E7%94%A8%E6%A4%8D%E7%89%A9"[%E4%B8%8D%E5%8A%A0%E6%9D%83:%E6%89%A9%E5%B1%95]) OR "%E4%B8%AD%E8%8D%AF"[%E4%B8%8D%E5%8A%A0%E6%9D%83:%E6%89%A9%E5%B1%95]) OR "%E4%B8%AD%E8%8D%89%E8%8D%AF"[%E4%B8%8D%E5%8A%A0%E6%9D%83:%E6%89%A9%E5%B1%95]) AND (("%E8%9E%BA%E6%9D%86%E8%8F%8C%E6%84%9F%E6%9F%93"[%E6%A0%87%E9%A2%98] OR "%E8%9E%BA%E6%9D%86%E8%8F%8C%E6%84%9F%E6%9F%93"[%E6%91%98%E8%A6%81] OR "%E5%B9%BD%E9%97%A8%E8%9E%BA%E6%9D%86%E8%8F%8C%E6%84%9F%E6%9F%93"[%E6%A0%87%E9%A2%98] OR "%E5%B9%BD%E9%97%A8%E8%9E%BA%E6%9D%86%E8%8F%8C%E6%84%9F%E6%9F%93"[%E6%91%98%E8%A6%81]) OR ("%E8%9E%BA%E6%9D%86%E8%8F%8C%E6%84%9F%E6%9F%93"[%E4%B8%8D%E5%8A%A0%E6%9D%83:%E6%89%A9%E5%B1%95]))')) |
